# Exploring Phytochemicals of Traditional Medicinal Plants Exhibiting Inhibitory Activity Against Main Protease, Spike Glycoprotein, RNA-dependent RNA Polymerase and Non-Structural Proteins of SARS-CoV-2 Through Virtual Screening

**DOI:** 10.3389/fphar.2021.667704

**Published:** 2021-07-08

**Authors:** Saranya Nallusamy, Jayakanthan Mannu, Caroline Ravikumar, Kandavelmani Angamuthu, Bharathi Nathan, Kumaravadivel Nachimuthu, Gnanam Ramasamy, Raveendran Muthurajan, Mohankumar Subbarayalu, Kumar Neelakandan

**Affiliations:** ^1^ Department of Plant Molecular Biology and Bioinformatics, Tamil Nadu Agricultural University, Coimbatore, India; ^2^ Department of Plant Biotechnology, Tamil Nadu Agricultural University, Coimbatore, India; ^3^ Centre for Plant Molecular Biology and Biotechnology, Tamil Nadu Agricultural University, Coimbatore, India; ^4^ Tamil Nadu Agricultural University, Coimbatore, India

**Keywords:** nCOVID-19, SARS-CoV-2, molecular docking, cyanin, amentoflavone, agathisflavone

## Abstract

Severe Acute Respiratory Syndrome Corona Virus 2 (SARS-CoV-2) being a causative agent for global pandemic disease nCOVID’19, has acquired much scientific attention for the development of effective vaccines and drugs. Several attempts have been made to explore repurposing existing drugs known for their anti-viral activities, and test the traditional herbal medicines known for their health benefiting and immune-boosting activity against SARS-CoV-2. In this study, efforts were made to examine the potential of 605 phytochemicals from 37 plant species (of which 14 plants were endemic to India) and 139 antiviral molecules (Pubchem and Drug bank) in inhibiting SARS-CoV-2 multiple protein targets through a virtual screening approach. Results of our experiments revealed that SARS-CoV-2 M^Pro^ shared significant disimilarities against SARS-CoV M^Pro^ and MERS-CoV M^Pro^ indicating the need for discovering novel drugs. This study has screened the phytochemical cyanin (*Zingiber officinale*) which may exhibit broad-spectrum inhibitory activity against main proteases of SARS-CoV-2, SARS-CoV and MERS-CoV with binding energies of (−) 8.3 kcal/mol (−) 8.2 kcal/mol and (−) 7.7 kcal/mol respectively. Amentoflavone, agathisflavone, catechin-7-o-gallate and chlorogenin were shown to exhibit multi-target inhibitory activity. Further, *Mangifera indica, Anacardium occidentale, Vitex negundo, Solanum nigrum, Pedalium murex, Terminalia chebula, Azadirachta indica, Cissus quadrangularis, Clerodendrum serratum and Ocimum basilicum*aree reported as potential sources of phytochemicals for combating nCOVID’19. More interestingly, this study has highlighted the anti-viral properties of the traditional herbal formulation “Kabasura kudineer” recommended by AYUSH, a unit of Government of India. Short listed phytochemicals could be used as leads for future drug design and development. Genomic analysis of identified herbal plants will help in unraveling molecular complexity of therapeutic and anti-viral properties which proffer lot of chance in the pharmaceutical field for researchers to scout new drugs in drug discovery.

## Introduction

Novel Coronavirus disease (nCOVID’19) caused by SARS-CoV-2 virus has become a global threat and WHO has declared it as a pandemic. nCOVID’19 is the third life threatening virus in the SARS family of viruses after SARS-CoV occurred during 2002–03 and MERS-CoV during 2012 (Lee et al., 2003; Cheng et al., 2007; Zaki et al., 2012; De Groot et al., 2013). It is named as a novel Coronavirus as it shares significant dissimilarity against other members of the SARS family of viruses *viz.,* SARS-CoV (30%) and MERS-CoV (60%) (Xu et al., 2020). Its unique genetic makeup has made it not responsive to available medical treatments and necessitated search for novel targets for vaccine development and drugs for effective prevention and treatment of nCOVID’19.

Exploding increase in the nCOVID’19 affected cases has brought this globe to a halt. Scientific community is trying to unravel genome complexity of nCOVID’19 for identifying novel targets for development of vaccines, screen available anti-viral drugs for effective management and short listing effective botanicals for therapeutic interventions. This has resulted in the accumulation enormous genomic information of nCOVID’19 in the public domain (https://www.ncbi.nlm.nih.gov/genbank/sars-cov-2-seqs/). Genomic analysis of nCOVID’19 revealed that it is approximately 30 kb in size (NCBI Accession # NC_045512) and further investigations identified three key genes viz., 1) coronavirus main protease (3CL^pro^)/papain-like protease (PL^pro^); 2) RNA-dependent RNA polymerase (RdRp) and 3) spike glycoprotein (S protein) as potential targets for drug designing (Elfiky, 2020; Sampangi-Ramaiah et al., 2020).

Screening of existing antiviral drugs including interferon α (IFN-α), lopinavir/ritonavir, chloroquine phosphate, ribavirin, chloroquine, hydroxychloroquine and arbidol is in progress and many of these experiments require pre-clinical and clinical validation (Elfiky, 2020). Ineffectiveness of existing anti-viral drugs have made the doctors to resort using traditional medicines in nCOVID’19 treatments (Yang et al., 2014). Several attempts have been made to exploit the potential of several herbal products having potential to inhibit the main protease (Mpro)/chymotrypsin-like protease (3CLpro) using molecular modeling and docking studies (Colson et al., 2020; Dong et al., 2020; Touret and de Lamballerie, 2020). Elfiky (2020) made an attempt to screen 27 different ligands present in commonly used herbals of Indian cuisines against SARS-CoV-2 Main protease and identified 15 different ligands effective in binding the viral protease. Yang et al. (2014) made a systematic review of herbal drugs used in the effective treatment of SARS-CoV and MERS-CoV and emphasized the urgent need for evolving procedures involving complementary and alternative treatments in managing nCOVID’19. Studies conducted so far have made attempts by using limited number of ligands which may hinder discovery of effective viral inhibitor in the herbal gene pool. In this context, short listing potential herbal drugs effective against nCOVID’19 through *in silico* docking of globally available ligands and validating them through laboratory and clinical trials is one of the viable approaches in managing this pandemic. India is one of the richest biodiversity centers in the world and known for its vast repository of medicinal plants. Considering India’s richest biodiversity of herbal medicinal plants and regular use of such medicinal plants in Indian health care system, the present study was undertaken to screen about 744 ligands including small molecules and phytochemicals from 37 different Indian medicinal plants against seven different protein targets of nCOVID’19 through molecular docking. Protein-Ligand interactions were analyzed carefully to shortlist potential small molecules and phytochemicals for drug development.

## Materials and Methods

### Phylogenetic Analysis of Main Protease of nCOVID’19

Protein sequence of SARS-CoV-2 encoding for main protease (PDB ID: 5R81) was used for PSI BLAST (NCBI) (Altschul et al., 1997) search to identify its homologs for understanding the evolutionary relationship with main proteases of other viruses. Multiple sequence alignment and phylogenetic analysis of SARS-CoV-2 main protease with other viral proteins was done using MAFFT server (Katoh et al., 2019).

### Virtual Screening of Herbal Ligands Against Potential Targets of nCOVID’19

#### Protein Targets

Coronavirus genome was reported to encode for 29 proteins, out of which the main protease is considered to be an important drug target. ORF1ab of the coronavirus genome contains 15 polypeptide chains encoding for non-structural proteins (NS proteins). Another part of the genome encodes for envelope and coat proteins. Availability of X-ray crystal structures for most of the proteins in the coronavirus two genome facilitates virtual screening to search for potential inhibitors. We have performed molecular docking of 744 ligands against seven different target proteins of the nCOVID’19 genome (Table 1). In addition, virtual screening was also performed against M^Pro^ of SARS CoV (PDB ID: 2GZ9) and MERS CoV (PDB ID: 5C3N) for identifying inhibitors against main protease of all three viruses and inhibitors specific to nCOVID’19.

**TABLE 1:** Protein targets with their PDB ID and the binding site volume.

S. No	PDB ID	Protein name	Binding site volume (Å) units
1	2GZ9	SARS-CoV M^Pro^	328.045
2	5C3N	MERS-CoV M^Pro^	317.710
3	5R81	SARS-CoV2 M^Pro^	308.363
4	6M0J	Spike glycoprotein	8,071.179 (interface of ACE and spike protein)
5	6M71	RNA-dependent RNA polymerase	4,977.808
6	6W4H	NSP16 - NSP10	316.761
7	6W02	NSP3	242.332
8	6W01	NSP15	212.386
9	6W4B	NSP9	121.103

#### Ligand Library Preparation

Chemical structures of all the small molecules were retrieved from Dukes database (1992–2016), PubChem (Kim et al., 2019) and DrugBank (Wishart et al., 2018). From the DrugBank database, 99 chemical structures of drugs approved for the treatment of respiratory diseases and compounds exhibiting antiviral activity were collected. Forty chemical compounds with COVID 19 antiviral property retrieved from pubchem database were also included in the ligand dataset. Information regarding the origin, traditional use and protocol were obtained from literature, Indian medicinal plants database (http://www.medicinalplants.in/) and Indian Medicinal Plants, Phytochemistry and Therapeutics (https://cb.imsc.res.in/imppat/home) database. Structures of 605 phytochemicals belonging to 37 different herbals and spices used in South Indian Traditional Medicine were also used for virtual screening (Supplementary Table S1). Among the 37 herbals, 14 herbals were found native to India (Table 2) namely, *Abutilon indicum* (Thuthi)*, Ocimum tenuiflorum* (Tulsi)*, Pedalium murex* (Peru nerinji)*, Anacyclus pyrethrum* (Akara)*, Andrographis paniculata* (Nilavembu)*, Pepper nigrum* (Pepper)*, Azadirachta indica* (Neem)*, Phyllanthus niruri* (Keelanelli)*, Cissus_quadrangularis_L* (Perandai)*, Plectranthus amboinicus* (Karppuravalli)*, Clerodendrum serratum* (Kanduparangi)*, Solanum torvum* (sundakai)*, Solanum trilobatum* (Thoothuvalai) and *Terminalia chebula* (*Kadukai*)*.* Phytochemicals from different parts of the plant such as Root, Rhizome, pericarp leaves, Flower, Seed essential oil, Stem and Fruits was curated in order to find bioactive molecule that may have role in the inhibition of SARS CoV two targets.

**TABLE 2:** List of herbal plants used in the study for screening antiviral compounds and their distribution and medicinal uses.

S. No	Plant name/Tamil name	Geographical distribution of plants	Plant part	Medicinal use	References
1.	*Abutilon indicum (thuthi)*	Native to tropic and subtropical regions, found in Karnataka and Tamil nadu	Root, flower, leaf	Anti-oxidant, anti-bacterial, analgesic, anti-inflammatory, anti-cancer, hepato-protective, immuno-modulatory and larvicidal activities	Abat et al. (2017)
2.	*Acorus calamus (vasambu)*	Throughout asia	Leaves and rhizome	Anti-oxidant, anti-inflammatory, anti-ulcer, anti-diabetic, anti-microbial, wound-healing, antiraioprotective, pesticidal and insecticidal properties	Sharma et al. (2020)
3.	*Aegle marmelos (vilvam)*	Indian sub continent and southeast asia	Leaves and fruits	Antidiabetic, anticancerous, antifertility, antimicrobial, immunogenic, and insecticidal activities	Pathirana et al. (2020)
4.	*Anacyclus pyrethrum (akara)* *^a^*	Native to mediterranean europe and parts of north africa, but also naturalized in other parts of europe, India and Pakistan	Root	Antidiabetic, sialagogue, aphrodisiac, immunostimulant, antidepressant, antimicrobial, insecticide, anesthetic, anti-inflammatory Anticonvulsant, antioxidant	Pandey et al. (2018)
5.	*Andrographis paniculata (nilavembu)* *^a^*	Native to India and Sri Lanka	Plant	Anticancer, antidiarrheal, antihepatitis, anti-hiv, antihyperglycemic, anti-inflammatory, antimicrobial, antimalarial, antioxidant, cardiovascular, cytotoxic, hepatoprotective, immunostimulatory, and sexual dysfunctions	Hossain et al. (2014)
6.	*Azadirachta indica (neem)*	Indian sub continent and throughout south asia	Leaves	Anti-inflammatory, antiarthritic, antipyretic, hypoglycemic, antigastric ulcer, antifungal, antibacterial, and antitumour activities	Alzohairy, (2016)
7.	*Brassica oleracea (cabbage)*	Originated from europe and distributed in caribean countries, Indonesia, Malaysia and India	Root, shoot, stem, leaves, leaf buds, flower buds, florets, landraces, sprouts, inflorescence, seeds, seed oil, and callus	Antimicrobial, antibacterial, antidiabetic, antimalarial, antiaging, antiulcer, anti-hyperglycemic, anti-hyperlipidemic, anti-proliferative, neuroprotective, antidiabetic, anti-genotoxic and antioxidant activities	Chen et al. (2016)
8.	*Carica papaya (papaya)*	Originated from mesoamerica and distributed in other tropical and subtropical regions of the world	Leaves, root, fruit, seeds	Antiparasitic, antiseptic, antiparasitic, antimicrobial, antiinflammatory, antihyperlipidemic, antihypertensive and antidiabetic	Vij and Prashar, (2015)
9.	*Cissus_quadrangularis_l (perandai)*	India, Bangladesh and Srilanka	Stem	Anti-ulcer, anti-bacterial, anxiolytic, antipyretic, antidiabetic, bone healing, antioxidant and anti-inflammatory properties	Rex and Ravi, (2020)
10.	*Clerodendrum serratum (kanduparangi)* *^a^*	Bengal, Odisha and Peninsular India	Plant, root and leaves	*Anti-oxidant, anti-carcinogenic, hepatoprotective, wound healing, anti-allergic*	Kumar and Nishteswar, (2013)
11.	*Cocosnucifera (coocnut leaf extract)*	South East Asia	Leaves, root, coconut oil, shell, fruit	Antihelminthic, anti-inflammatory, antinociceptive, antioxidant, antifungal, antimicrobial, and antitumor activities	DebMandal and Mandal, (2011)
12.	*Coriandrum sativum (coriander)*	Wetern Asia and Southern Europe	Fruits, leaves, plant	Antibacterial, antifungal and anti-oxidative activities	Mandal and Mandal, (2015)
13.	*Curcuma longa (turmeric)*	Indan subcontinent and South East Asia	Root	Anti-infammatory, antioxidant, antimutagenic, antidiabetic, antibacterial, hepatoprotective, expectorant and anticancerous	Krup et al. (2013)
14.	*Cyprus rotundus (korai kilangu)* *^a^*	Southern Asia, Africa, Southern and Central Europe	Root, rhizome, essential oil and tuber	Antiandrogenic, antibacterial, anticancerous, anticonvulsant, antidiabetic, antidiarrheal, antigenotoxic, anti-inflammatory, antilipidemic, antimalarial, antimutagenic, antiobesity, antioxidant, anti-uropathogenic, hepatoprotective, cardioprotective, neuroprotective, and nootropic agent	Peerzada et al. (2015)
15.	*Glycyrrhiza glabra (liquo rice)*	Western Asia, Northern Africa and Eurasia	Liquorice extract, root	Antibacterial, antioxidant, antimalarial, antispasmodic, antiinflammatory and anti-hyper glycemic antiulcer, antiviral, anti-hepatotoxic, antifungal properties	Kaur et al. (2013a)
16.	*Hygrophila auriculata (mulli ver)* ^*a*^	Tropical asi and africa	Root, stem	Anti-nociceptive, antitumor, antioxidant, hepatoprotective, hypoglycemic, hematinic, diuretic, free radical scavenging, anthelmintic, anti-inflammatory, antipyretic, anabolic and androgenic activities	(Hussain et al., 2010)
17.	*Ipomea carnea (neiveli kaatamanakku)*	South America and found in Tamil nadu	Plant extract	Glycosidase inhibitory activities, antioxidant antiinflammatory, antidiabetic, wound healing antimicrobial, immunomodulatory, cardiovascular, abortifacient, antifungal, anticancer and hepatoprotective	(Saxena, Tyagi, kumar, singh)
18.	*Justicia adhatoda (aada thoda)*	Asia	Plant, flower, leaf	Antitussive, abortifacient, antimicrobial, cardiovascular protection, anticholinesterase, antiinflammatory	Kaur et al. (2013b)
19.	*Malus domestica(apple)*	Central Asia and Europe and cultivated world wide	Fruit	Antioxidant, antiproliferative, anti-depressant, antiinflammatory, anti-microbial	Lobo and Satish, (2019)
20.	*Nigella sativa (fennel)*	Southwest Asia, Southern Europe, North Africa	Seed	Antidiabetic, anticancer, immunomodulator, analgesic, antimicrobial, anti-inflammatory, spasmolytic, bronchodilator, hepato-protective, renal protective, gastro-protective, antioxidant properties	Ahmad et al. (2013)
21.	*Ocimum basilicum (thiruneetrupachai)*	South East Asia and Central Africa	Leaves	Anti-cancer, radioprotective, anti-microbial, anti-inflammatory effects, immunomodulatory, anti-stress, anti-diabetic, anti-pyretic, anti-arthritic, anti-oxidant, as a prophylactic agent and in cardiovascular disease	Shahrajabian et al. (2020)
22.	*Ocimum tenuiflorum (tulsi)*	India and South East Asia	Leaves, stem, flower, root, seeds and even whole plant	Antifertility, anticancer, antidiabetic, antifungal, antimicrobial, hepatoprotective, cardioprotective, antiemetic, antispasmodic, analgesic, adaptogenic and diaphoretic actions	Prakash and Gupta, (2005)
23.	*Pedalium murex (peru nerinji)*	Southern part and deccan region of India and Ceylon	Whole plant, root, seed, leaf	Antiulcerogenic, nephroprotective, hypolipidemic, aphrodisiac, antioxidant, antimicrobial and insecticidal activities	Patel et al. (2011a)
24.	*Pepper nigrum (pepper)*	South asia (India, southern Thailand, Malaysia)	Fruit, seed, seed essential oil	Antihypertensive and antiplatelets, antioxidant, antitumor, antiasthmatics, antipyretic, analgesic, anti-inflammatory, anti-diarrheal, antispasmodic, anxiolytic, antidepressants, hepato-protective, immuno-modulatory, antibacterial, antifungal, anti-thyroids, antiapoptotic, anti-metastatic, antimutagenic, anti-spermatogenic, anticolon toxin, insecticidal and larvicidal activities	Damanhouri and Ahmad, (2014)
25.	*Phyllanthus niruri (keelanelli)*	India tropical and coastal areas	Leaves, whole plant	Antiviral, antibacterial, antiplasmodial, anti-inflammatory, antimalarial, antimicrobial, anticancer, antidiabetic, hypolipidemic, antioxidant, hepatoprotective nephroprotective and diurectic properties	Patel et al. (2011b)
26.	*Piper betle (vetrilai)*	Cultivated in India, srilanka, Malaysia, Thailand, Taiwan and southeast asian countries	Leaves	Antimicrobial, gastroprotective, wound healing, hepatoprotective, antioxidant, anti-fertility on male rats, and antimotility effects on washed human spermatozoa	Alam et al. (2013)
27.	*Piper longum (thippili)* *^a^*	South Asia	Flower, root	Anti-diabetic and anti-hyperlipidemic, hepatoprotective, neuroprotective, cardioprotective, anti-bacterial, aphrodiasiac, relieves respiratory disorders	Srivastava, (2014)
28.	*Plectranthus amboinicus (karppuravalli)*	India, Sri Lanka	Leaves	Antimicrobial, antifungal, anti-inflammatory, antidiabetic, anxiolytic, antineoplastic, analgesic, antimalarial, antibiofilm efficacy, diuretic, wound healing, skincare, respiratory disorders, and antiplatelet aggregation	Kumar et al. (2020)
29.	*Salvia officinalis (sage)*	Middle east and naturalized throughout the world	Leaf	Anticancer, anti-inflammatory, antinociceptive, antioxidant, antimicrobial, antimutagenic, antidementia, hypoglycemic, and hypolipidemic effects	Ghorbani and Esmaeilizadeh, (2017)
30.	*Solanum nigrum (manathakkali)*	Eurasia	Leaves	Anti-oxidant, anti-cancer	Campisi et al. (2019)
31.	*Solanum torvum (sundakai)*	India	Fruit, leaves	Antihypertensive, antioxidant, cardiovascular, anti-platelet aggregation activities, anti-microbial, sedative, digestive, hemostatics and diuretic activities	Agrawal et al. (2010)
32.	*Solanum trilobatum (thoothuvalai)* *^a^*	Southern India	Leaf, stem, root, flowers and berries	Anti-microbial, anti-inflammatory, antioxidant, cytotoxic, anti-diabetic and immunomodulatory activities	Balakrishnan et al. (2015)
33.	*Syzygium aromaticum (lavangam)* *^a^*	Maluku islands (or moluccas) in Indonesia and available throughout the year owing to different harvest seasons in different countries	Flower	Analgesic, antioxidant, anticancer, antiseptic, anti-depressant, antispasmodic, anti-inflammatory, antiviral, antifungal, and antibacterial	Batiha et al. (2020)
34.	*Terminalia bellirica (thanthrikai)* *^a^*	Southeast Asia	Fruit	Antisecretory, analgesic, antihypertensive, antidiarrhoeal, antimicrobial antidiabetic, antioxidant, antiulcer, antipyretic, hepatoprotective, anticancer, angiogenesis, antidepressant-like and anti-urolithiatic	Kumar and Khurana, (2018)
35.	*Terminalia chebula (kadukai)* *^a^*	South asia from India and Nepal	Fruit	Antibacterial, antifungal, antiviral, antidiabetic, antimutagenic, antioxidant, antiulcer and wound healing	Kolla et al. (2017)
36.	*Vitex negundo (nochi)*	Asia, southern and Eastern africa	Leaves	Analgesic, anti-inflammatory, antimicrobial, antioxidant, hepatoprotective, antihistamine, and antiasthmatic	Meena et al. (2011)
37.	*Zingiber officinale (chukku)* *^a^*	Maritime Southeast Asia	Rhizome	Cardioprotective, antiinflammatory, antimicrobial, antioxidant, antiulcer, anticlotting and anticancer	Khan et al. (2018)

a Kabasura kudineer-plant compounds.

Known active ingredients of eight herbal plants included in the Tamil traditional medicine “Kabasura Kudineer” (meaning water capable of boosting immunity) were also included in the screening. Overall, a total of 744 small molecules/ligands (Supplementary Table S1: a-c) were used for virtual screening against seven different protein targets. Interactions of phytochemicals were compared with drugs such as hydroxycholoroquine (Pubchem ID: 3652), chloroquine (Pubchem ID: 2719) and ivermectin (Pubchem ID: 6427057) retrieved from pubchem database.

#### Virtual Screening

Virtual screening was performed using *Python* Prescription Virtual Screening tool (PyRx 0.8) containing AutoDock Vina module (Dallakyan and Olson, 2015). Protein structure was prepared using SWISS PDB Viewer by adding hydrogen atoms and energy minimization. Prepared protein structure was fed into the PyRx tool along with the structure of 744 ligands. Both the ligands and protein molecules were converted to pdbqt file using the AutoDock module of PyRx tool. Inhibitors are expected to bind in the active site/binding site of the protein to inhibit the function of the protein target. In the present study, binding sites were predicted using CASTP server (Tian et al., 2018) and the predicted sites were used for setting grid (XYZ dimensions: 25*25*25) in the AutoDock Vina for virtual screening experiment with the exhaustiveness value of 8. Predicted binding sites were also verified with protein-ligand binding sites of experimental structures for accuracy. Furthermore, phylogenetic analysis of SARS-CoV-2 M^Pro^ was carried out using PSI-BLAST (NCBI) (Altschul et al., 1997) and MAFFT server (Katoh et al., 2019). Top 10 ligand hits against each of the seven protein targets were taken for further analysis. 2D and 3D interactions between the protein-ligand were analyzed using Schrodinger Maestro visualizer and BIOVIA discovery studio visualizer 2020 (Accelrys Inc. San Diego, CA, United States) software. ADME properties of top 10 ligands screened against individual protein targets were predicted using SWISSADME server (http://www.swissadme.ch **)** (Supplementary Table S2).

## Results and Discussion

### Phylogenetic Analysis on Coronavirus Main Proteases

**FIGURE 1:**
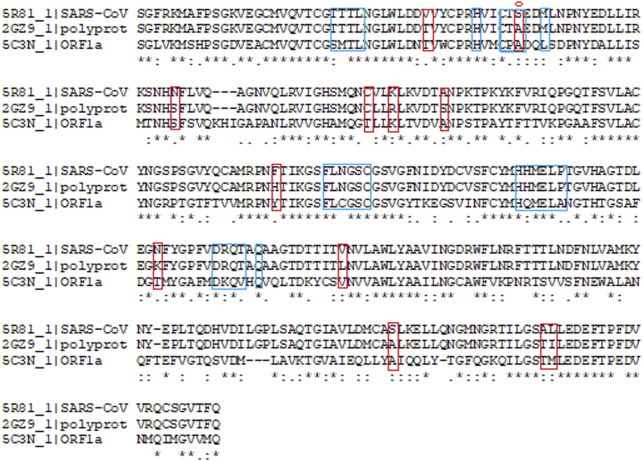
Multiple sequence alignment of SARS-CoV-2 (PDB ID: 5R81), SARS-CoV (PDB ID: 2GZ9) and MERS-CoV (PDB ID: 5C3N) main proteases. Binding site residues are marked with blue box. Red marked residues indicate the change observed between SARS-CoV-2 and SARS CoV. * denotes the conserved regions with identical residues. Serine to Alanine change at the 45th position observed in the binding site is marked with circle.

Main protease (M^pro^, also called 3CL^pro^) is considered as one of the important molecular targets for designing novel drugs against coronaviruses (Anand et al., 2003). With a view to design drugs/inhibitors specifically targeting main protease of nCoVID’19, *in-silico* analysis was performed using main protease sequences of SARS-CoV-2, SARS-CoV and MERS-CoV. Multiple sequence alignment identified 12 significant differences between main proteases of SARS-CoV and SARS-CoV-2 (Figure 1). Out of the 12 differences, S45 to A45 was found to reside within in the binding site of SARS-CoV-2 main protease. This may play a crucial role in determining differential binding affinity of the two proteases.

**FIGURE 2:**
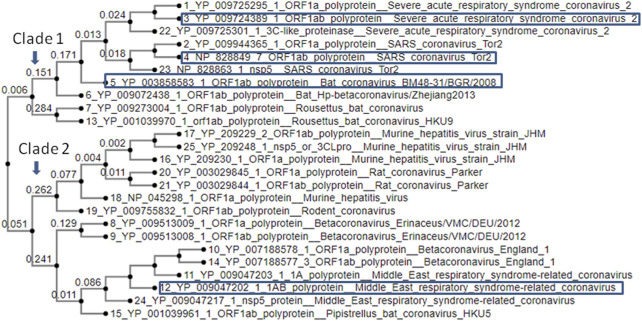
Phylogenetic analysis of SARS-CoV-2M^PRO^ with other CoV M^PRO^ proteins.

Phylogenetic analysis of SARS-CoV2M^Pro^ with other CoV M^Pro^ sequences sharing >50 percentage similarity revealed its significant genetic relatedness with SARS CoV (96.08% similarity) and bat CoV (76.84% similarity) (Figure 2). Also it shared significant similarity with ORF1ab of Rousettus bat coronavirus. Main protease of nCoVID’19 shared only 50.65% similarity against main proteases of MERS-CoV. Above results clearly indicated the need for a highly specific novel drug specifically inhibiting main proteases of SARS-CoV-2.

### Virtual Screening of Potential Herbal Ligands Against Major Protein Targets of SARS-CoV-2

Virtual screening of 744 ligands belonging to small molecules and active compounds from 37 medicinal herbs against seven major protein targets of nCOVID’19 predicted probable SARS- CoV-2 inhibitors. Information regarding the binding site residues predicted using CASTp server is provided in Table 3. Binding affinity of drugs such as hydroxychloroquine, chloroquine and ivermectin are provided in Table 4 which was used as reference for comparison of phytochemicals. Top 10 hits reported with higher binding affinity for each target protein is considered for downstream analysis (Table 5). Seven molecular targets of SARS-CoV-2 include, Main protease (PDB ID: 5R81), RNA-dependent RNA polymerase (RdRp) (PDB ID: 6M71), NSP3 (PDB ID: 6W02), NSP9 (PDB ID: 6W4B), NSP10-NSP16 (PDB ID: 6W4H), NSP15 (PDB ID: 6W01) and Spike protein (PDB ID: 6M0J).

**TABLE 3:** Binding site residues of CoV protein targets used for virtual screening.

Protein name	Binding site residues
Main protease (SARS-CoV)	25 THR, 26 THR, 27 LEU, 41 HIS, 44 CYS, 45 THR, 46 ALA, 49 MET, 140 PHE, 141 LEU, 142 ASN, 143 GLY, 144 SER, 145 CYS, 163 HIS, 164 HIS, 165 MET, 166 GLU, 167 LEU, 168, PRO, 172 HIS, 188 ARG, 189 GLN, 190 THR, 192 GLN
Main protease (MERS-CoV)	1 SER, 25 MET, 26 THR, 27 LEU, 41 HIS, 42 VAL, 44 CYS, 46 ALA, 49 LEU, 143 PHE, 144 LEU, 145 CYS, 146 GLY, 148 CYS, 166 HIS, 167 GLN, 168 MET, 169 GLU, 170 LEU, 171 ALA, 175 HIS, 190 ASP, 191 LYS, 192 GLN, 193 VAL, 194 HIS, 195 GLN, 196 VAL
Main protease (SARS-CoV-2)	24 THR, 25 THR, 26 THR, 27 LEU, 41 HIS, 44 CYS, 45 THR, 46 SER, 49 MET, 140 PHE, 141 LEU, 142 ASN, 143 GLY, 144 SER, 145 CYS, 163 HIS, 164 HIS, 165 MET, 166 GLU, 167 LEU, 168 PRO, 187 ASP, 188 ARG, 189 GLN, 190THR, 192 GLN
Spike glycoprotein	**Chain E**–403 ARG, 405 ASP, 406 GLU, 408 ARG, 409 GLN, 415 THR, 416 GLY, 417 LYS, 420 ASP, 421 TYR, 449 TYR, 453 TYR, 456 PHE, 493 GLN, 494 SER, 495 TYR, 496 GLY, 497 PHE, 501 ASN, 502 GLY, 503 VAL, 504 GLY, 505 TYR
RNA-dependent RNA polymerase NSP 12	164 ASP, 166 VAL, 167 GLU, 429 PHE, 430 LYS, 431 GLU, 436 GLU, 437 LEU, 438 LYS, 439 HIS, 440 PHE, 441 PHE, 442 PHE, 452 ASP, 455 TYR, 456 TYR, 494 ILE, 496 ASN, 497 ASN, 499 ASP, 500 LYS, 66,501 SER, 503 GLY, 507 ASN, 511 LYS, 540 THR, 541 GLN, 542 MET, 543 ASN, 544 LEU, 545 LYS, 546 TYR, 547 ALA, 548 ILE, 549 SER, 550 ALA, 551 LYS, 553 ARG, 554 ALA, 555 ARG, 556 THR, 557 VAL, 558 ALA, 559 GLY, 565 THR, 568 ASN, 569 ARG, 572 HIS, 573 GLN, 576 LEU, 577 LYS, 580 ALA, 588 VAL, 589 ILE, 590 GLY, 591 THR, 592 SER, 593 LYS, 594 PHE, 598 TRP, 601 MET, 602 LEU, 616 GLY, 617 TRP, 618 ASP, 619 TYR, 620 PRO, 621 LYS, 622 CYS, 623 ASP, 624 ARG, 665 GLU, 667 VAL, 676 LYS, 680 THR, 681 SER, 682 SER, 683 GLY, 684 ASP, 685 ALA, 686 THR, 687 THR, 688 ALA, 689 TYR, 691 ASN, 756 MET, 758 LEU, 759 SER, 760 ASP, 761 ASP, 762 ALA, 763 VAL, 792 VAL, 793 PHE, 795 SER, 797 ALA, 798 LYS, 799 CYS, 800 TRP, 810 HIS, 811 GLU, 812 PHE, 813 CYS, 814 SER, 815 GLN, 816 HIS, 833 ASP, 835 SER, 836 ARG, 837 ILE, 840 ALA, 841 GLY, 843 PHE, 844 VAL, 845 ASP, 847 ILE, 848 VAL, 854 LEU, 855 MET, 857 GLU, 858 ARG, 859 PHE, 861 SER, 862 LEU, 864 ILE, 865 ASP **C chain** - 3MET, 4SER, 7LYS, 40LEU, 41LEU, 43LYS
NSP16 - NSP10	6841 ASN, 6844 LYS, 6845 TYR, 6867HIS, 6868PHE, 6869 GLY, 6870ALA, 6871GLY, 6872SER, 6873ASP, 6878PRO, 6879 GLY, 6896SER, 6897ASP, 6898LEU, 6899ASN, 6900 ASP, 6901PHE, 6911GLY, 6912ASP, 6913CYS, 6928ASP, 6929 MET, 6930 TYR, 6931 ASP, 6932 PRO, 6933LYS, 6947 PHE, 6968 LYS
NSP3	6 PHE, 7 SER, 8 GLY, 10 LEU, 11 LYS, 12 LEU, 18 ILE, 19 LYS, 20 ASN, 158 LYS, 161 TYR, 162 ASP, 165 VAL, 168 PHE
NSP15	69 GLU, 71 LYS, 90 LYS, 196 THR, 198 SER, 199 ARG, 200 ASN, 201 LEU, 202 GLN, 252 LEU, 255 LEU, 259 PHE, 266LEU, 268 ASP, 272 MET, 273 ASP. 274 SER, 275 THR, 277 LYS, 279 TYR, 295 VAL, 296 ILE, 297 ASP
NSP9	13 MET, 33 TYR, 38 GLY, 39 GLY, 40 ARG, 42 VAL, 57PHE, 58 PRO, 59 LYS, 60 SER, 66 ILE, 68 THR

**TABLE 4:** Binding affinity value of Hydroxychloroquine, Cholroquine and Ivermectin against SARS-COV-2 targets.

SARS-CoV-2Protein target	Hydroxychloroquine (pubchem ID: 3652) binding affinity (kcal/mol)	Chloroquine (pubchem ID: 2719). Binding affinity (kcal/mol)	Ivermectin (pubchem ID: 6427057). Binding affinity (kcal/mol)
SARS-CoV-2M^Pro^	−5.5	−4.9	−7.3
RdRp	−5.6	−5.4	−9.4
NSP3	−4.5	−4.2	−6.7
NSP9	−5.7	−5.2	−7.5
NSP16 - NSP10	−5.7	−5.3	−1.9
NSP15	−5.5	−5.4	−6.4
Spike glycoprotein	−5.3	−5.2	−8.2

**TABLE 5:** Details of shortlisted top 10 natural compounds/small molecules screened against various targets of coronaviruses.

Protein name	Binding affinity kcal/mol	Compound name	Puchem ID	Chemical structure	Bioavailability score	Solubility score/Class	Plant name
SARS COV main protease (PDB ID: 2GZ9)	−9.0	Rutin (ZINC4096846)	5,280,805	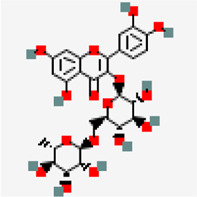	0.17	0.29/Soluble	*Terminalia chebula, Azadirachta indica and Ocimum basilicum*
	−8.7	Quercetin 3 gentiobioside (ZINC49783852)	5,320,834	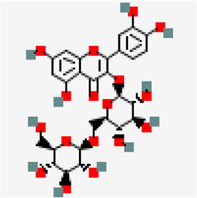	0.17	0.29/Soluble	*Solanum nigrum*
	−8.6	3-O-trans-caffeoyltormentic acid	44,584,640	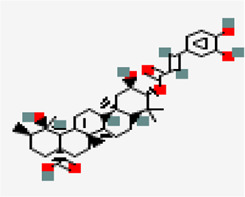	0.56	-5.99/moderately soluble	Antiviral compounds
	−8.5	(-)Epicatechin 3 o gallate (epicatechin)	107,905	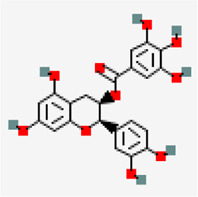	0.55	-3.09/soluble	*Camellia sinensis*
	−8.5	Corilagin (ZINC4098612)	73,568	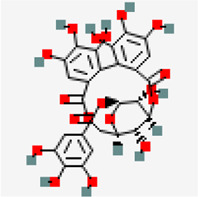	0.17	-0.51/soluble	*Terminalia bellirica*, *Terminalia chebula*
	−8.4	Quercetin galactoside (ZINC3973253)	5,281,643	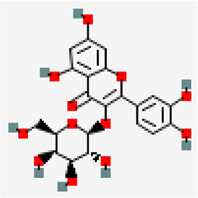	0.17	-1.51/soluble	Antiviral compounds
	−8.3	Quercetrin (ZINC4175638)	5,280,459	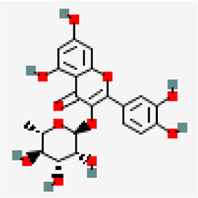	0.17	-2.08/soluble	*Azadirachta indica*
	−8.3	Isoquercetin (ZINC4096845)	5,280,804	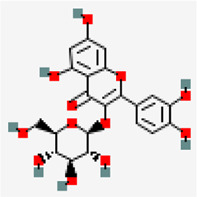	0.17	-1.51/soluble	*Ocimum basilicum, Terminalia chebula*
	−8.3	Acetoside (ZINC8234351)	5,281,800	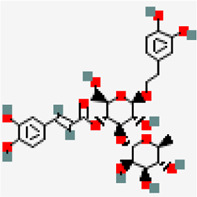	0.17	-0.22/soluble	*Clerodendrum serratum*
	−8.2	Cyanin (ZINC4097727)	441,688	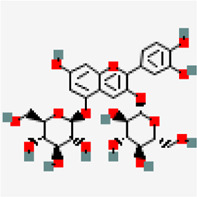	0.17	0.86/soluble	*Zingiber officinale*
SARS-COV-2 main protease (PDB ID: 5R81)	−8.2	Agathisflavone (ZINC4098505)	5,281,599	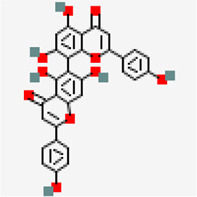	0.17	-8.7/Poorly soluble	*Anacardium occidentale*
	−8.1	Rubusic acid	101,297,651	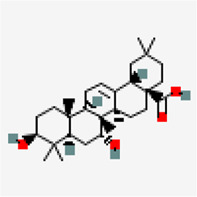	0.56	-5.3/moderately soluble	*Pedalium murex*
	−7.9	Solanocapsine	73,419	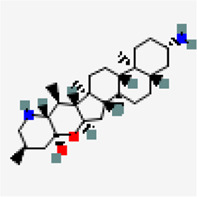	0.55	-4.2/moderately soluble	*Solanum nigrum*
	−7.7	Chlorogenin (ZINC84668707)	12,303,065	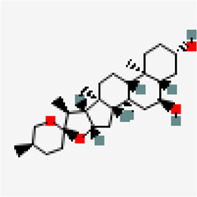	0.55	-3.69/soluble	*Solanum torvum*
	−7.7	Lupeol (ZINC4081455)	259,846	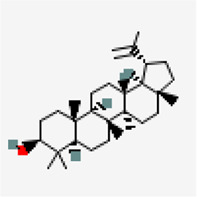	0.55	-6.74/poorly soluble	*Carica papaya* and *Azadirachta indica*
	−7.7	Cyanin (ZINC4097727)	441,688	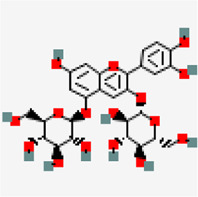	0.17	0.86/soluble	*Zingiber officinale*
	−7.7	3-O-trans-caffeoyltormentic acid	44,584,640	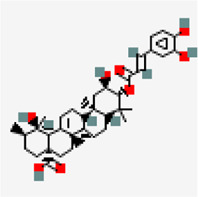	0.56	-5.99/moderately soluble	Antiviral compound
	−7.7	Luteolin 7-O-(6''-malonylglucoside)	5,281,669	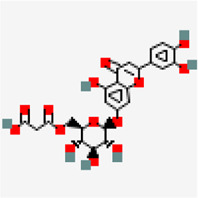	0.11	-2.05/soluble	*Vitex negundo*
	−7.6	Agnuside (ZINC4098330)	442,416	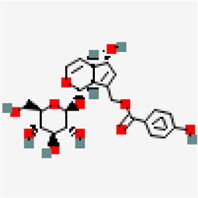	0.17	0.66/soluble	*Vitex negundo*
	−7.6	Luteolin 7-O-beta-D-glucoside (ZINC4096258)	5,280,637	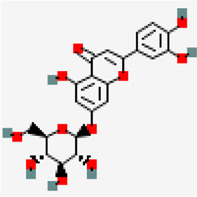	0.17	-2.1/soluble	*Vitex negundo*
MERS main protease (MERS) (PDB ID: 5C3N)	−8.6	Amentoflavone (ZINC3984030)	5,281,600	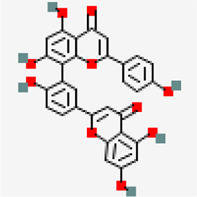	0.17	-8./poorly soluble	*Mangifera indica*
	−8.4	Corilagin (ZINC4098612)	73,568	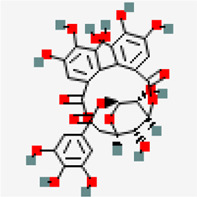	0.17	-0.51/soluble	*Terminalia bellirica, Terminalia chebula*
	−8.3	Cyanin (ZINC4097727)	441,688	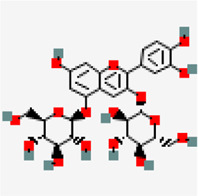	0.17	0.86/soluble	*Zingiber officinale*
	−8.2	Rutin (ZINC4096846)	5,280,805	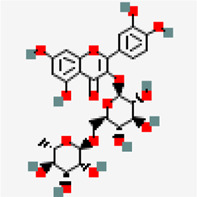	0.17	-0.29/soluble	*Terminalia chebula, Azadirachta indica and Ocimum basilicum*
	−8.2	Betulinic acid (ZINC4097714)	64,971	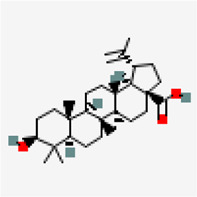	0.85	-5.7/moderately soluble	*Vitex negundo*
	−8.1	3.8'-biapigenin (ZINC17545581)	10,414,856	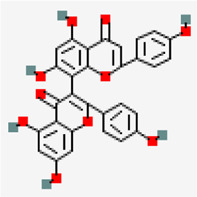	0.17	-8.7/poorly soluble	*antiviral compound*
	−8.0	(-)-Epicatechin-3-o-gallate (ZINC3978503)	107,905	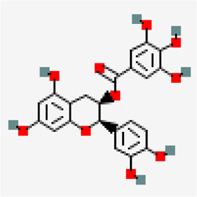	0.55	-3.09/soluble	*Camellia sinensis*
	−8.0	Chrysanthemin (ZINC4097706)	441,667	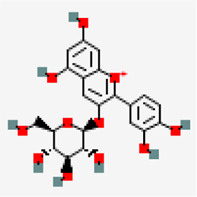	0.17	-0.93/soluble	*Zingiber officinale*
	−8.0	Vicenin-2 (ZINC4098604)	442,664	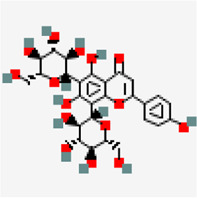	0.17	-0.27/soluble	*Ocimum basilicum*
	−8.0	Quercetin 3 gentiobioside (ZINC49783852)	5,320,834	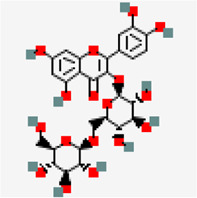	0.17	0.29/soluble	*Solanum nigrum*
RNA-dependent RNA polymerase (PDB ID: 6M71)	−9.4	Ivermectin (ZINC238808778) (DB00602)	6,427,057	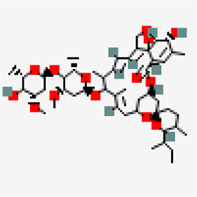	0.17	-3.89/soluble	FDA approved drug
	−9.3	Amentoflavone (ZINC3984030)	5,281,600	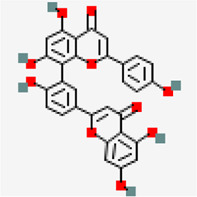	0.17	-8.7/poorly soluble	*Mangiferaindica*
	−9.0	Corilagin (ZINC4098612)	73,568	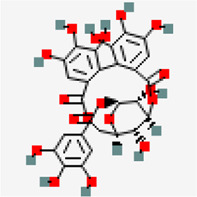	0.17	-0.51/soluble	*Terminalia bellirica, Terminalia chebula*
	−8.9	Agathisflavone (ZINC4098505)	5,281,599	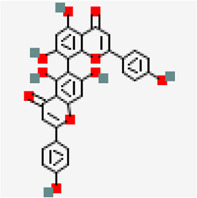	0.17	-8.7/poorly soluble	*Anacardium occidentale*
	−8.6	3-O-trans-caffeoyltormentic acid	44,584,640	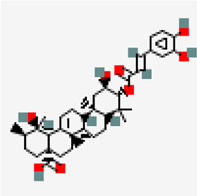	0.56	-5.99/moderately soluble	Antiviral compound
	−8.2	Arjungenin (ZINC38143755)	6,321,424	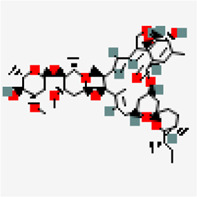	0.17	-3.89/soluble	*Terminalia chebula*
	−8.2	Crateogolic acid (ZINC4273446)	12,444,386	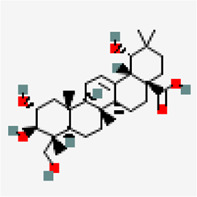	0.56	-3.9/soluble	*Syzygium aromaticum*
	−8.1	Maslinic acid (ZINC4273446)	73,659	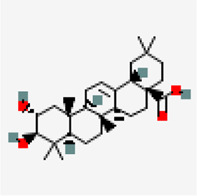	0.56	-5.3/moderately soluble	Antiviral compound
	−8.0	3.8'-biapigenin (ZINC17545,581))	10,414,856	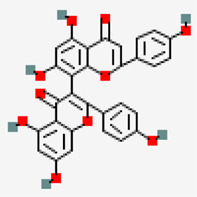	0.17	-8.7/Poorly soluble	*Zingiber officinale*
	−7.9	Cyanin	441,688	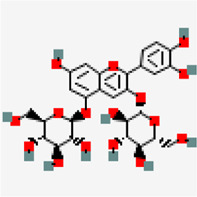	0.17	0.86/soluble	*Camellia sinensis*
NSP9 (PDB ID: 6W4B)	−9.6	Friedelin (ZINC4097720)	91,472	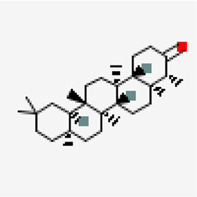	0.55	-7.88/poorly soluble	*Vitex negundo, Acoruscalamus*
	−9.3	N-methylsolasodine	21,573,751	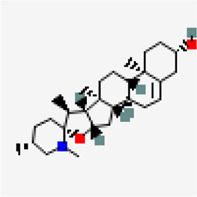	0.55	-4.46/moderately soluble	*Solanum nigrum*
	−8.9	Solasodine (ZINC8214722)	442,985	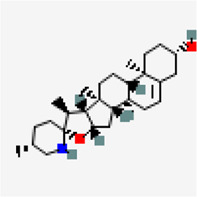	0.55	-4.8/moderately soluble	*Solanum nigrum, Solanum torvum*
	−8.9	Diosgenin (ZINC6857614)	99,474	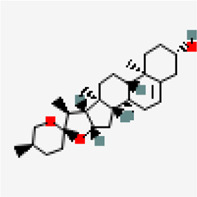	0.55	-4.49/moderately soluble	*Solanum nigrum, Pedalium murex*
	−8.6	Spirostan-3-ol (ZINC253504500)	3,035,446	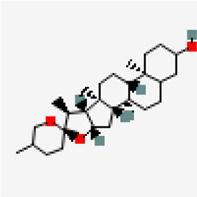	0.55	-4.51/Moderately soluble	*Solanum nigrum*
	−8.6	Solanocapsine	73,419	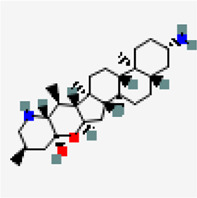	0.55	-4.22/moderately soluble	*Solanum nigrum*
	−8.5	Taraxerol (ZINC4082498)	92,097	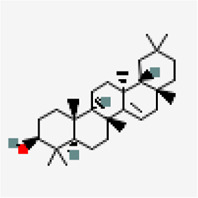	0.55	-7.16/Poorly soluble	*Cissus quadrangularis*
	−8.3	Amentoflavone (ZINC3984030)	5,281,600	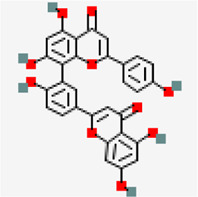	0.17	-8.7/Poorly soluble	*Mangifera indica*
	−8.2	Chlorogenin (ZINC84668707)	12,303,065	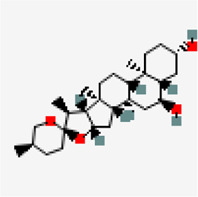	0.55	-3.69/soluble	*Solanum torvum*
	−8.1	Agathisflavone (ZINC4098505)	5,281,599	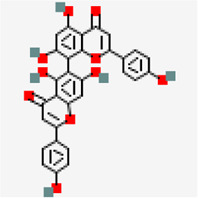	0.17	-8.7/poorly soluble	*Anacardium occidentale*
NSP15 (PDB ID: 6W01)	−9.2	Oleanolic acid (ZINC3785416)	10,494	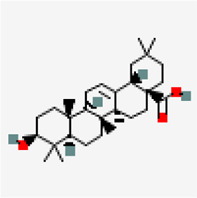	0.85	-6.12/poorly soluble	*Ocimum basilicum, Cyprus rotundus, Clerodendrum serratum, Ocimum tenuiflorum*
	−9.2	Ursolic-acid (ZINC3978827)	64,945	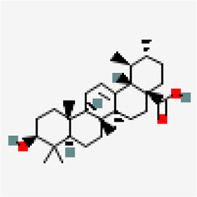	0.85	-5.67/moderately soluble	*Ocimum basilicum, Pedalium murex, Malus domestica, Ocimum tenuiflorum*
	−8.9	Crategolic acid (ZINC4273446)	73,659	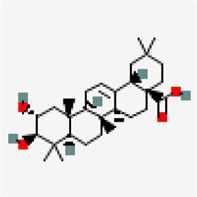	0.56	-5.3/Moderately soluble	*Syzygium aromaticum*
	−8.8	Arjungenin (ZINC38143755)	12,444,386	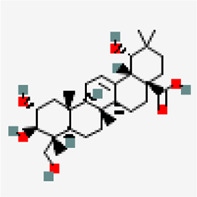	0.56	-3.9/soluble	*Terminalia chebula*
	−8.7	Hederagenin (ZINC3946009)	73,299	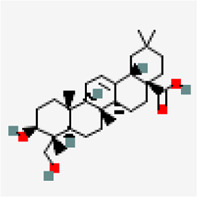	0.56	-5.55/moderately soluble	*Nigella sativa*
	−8.6	Triterpenoid (ZINC40164454)	451,674	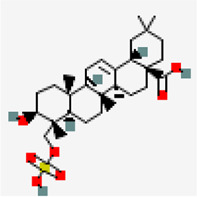	0.56	-5.12/moderately solule	*Abutilon indicum*
	−8.6	Beta-amyrin (ZINC3978270)	73,145	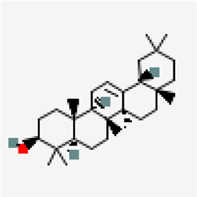	0.55	-7.16/poorly solube	*Cissus quadrangularis*
	−8.6	Friedelin (ZINC4097720)	91,472	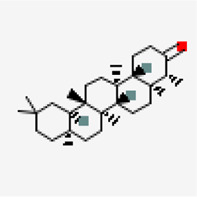	0.55	-7.88/poorly soluble	*Vitex negundo, Acorus calamus*
	−8.5	Catechin 7-O-gallate	471,393	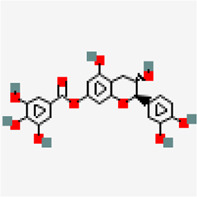	0.55	-3.09/soluble	*Camellia sinensis*
	−8.5	Arjunolic acid (ZINC31158038)	73,641	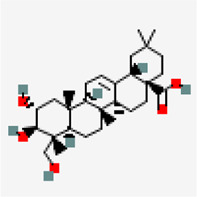	0.56	-4.72/moderately soluble	*Terminalia chebula*
NSP3 (PDB ID: 6W02)	−7.4	Amentoflavone (ZINC3984030)	5,281,600	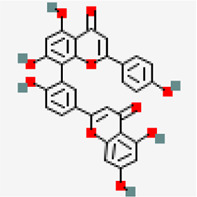	0.17	-8.7/poorly soluble	*Mangifera indica*
	−7.1	Luteolin 7-O-(6''-malonylglucoside)	5,281,669	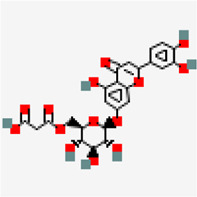	0.11	-2.05/soluble	*Vitex negundo*
	−6.8	Rubusic acid	101,297,651	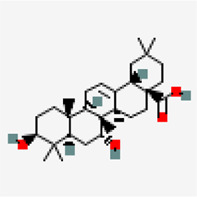	0.56	-5.3/moderately soluble	*Pedalium murex*
	−6.7	Acteoside (ZINC8234351)	5,281,800	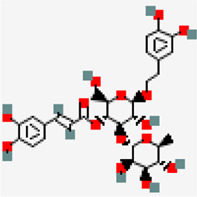	0.17	-0.22/soluble	*Clerodendrum serratum*
	−6.7	Ivermectin (ZINC238808778) (DB00602)	6,427,057	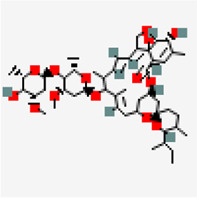	0.17	-3.89/soluble	FDA approved drug
	−6.7	Taraxerol acetate (ZINC31334747)	94,225	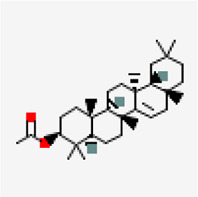	0.55	-7.77/poorly soluble	*Cissus quadrangularis*
	−6.6	Catechin 7-O-gallate	471,393	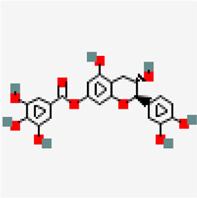	0.55	-3.09/soluble	Antiviral compound
	−6.6	Luteolin 7-O-beta-D-glucoside (ZINC4096258)	5,280,637	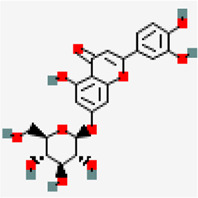	0.17	-2.1/soluble	*Vitex negundo*
	−6.6	Agathisflavone (ZINC4098505)	5,281,599	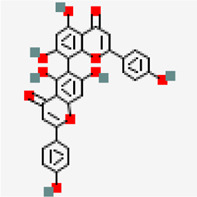	0.17	-8.7/poorly soluble	*Anacardium occidentale*
	−6.6	Luteolin-7-o-beta-d-glucopyranoside	5,291,488	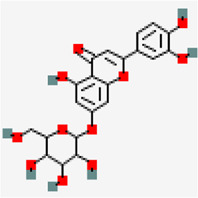	0.17	-2.1/soluble	*Vitex negundo*
NSP10-NSP16 (PDB ID: 6W4H)	−8.5	Amentoflavone (ZINC3984030)	5,281,600	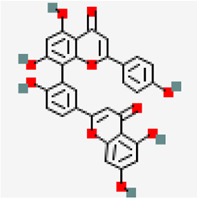	0.17	-8.7/poorly solbule	*Mangifera indica*
	−8.3	10-Methoxycamptothecin (ZINC4823972)	97,283	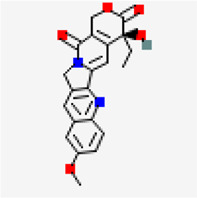	0.55	-5.93/moderately soluble	Antiviral compound
	−8.2	3.8'-biapigenin (ZINC17545581)	10,414,856	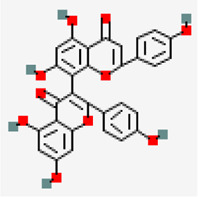	0.17	-8.7/poorly soluble	Antiviral compound
	−8.0	Taraxerol acetate (ZINC31334747)	94,225	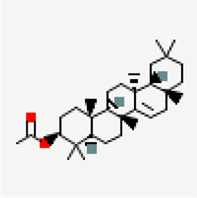	0.55	-7.77/poorly soluble	*Cissus quadrangularis*
	−7.8	Corilagin (ZINC4098612)	73,568	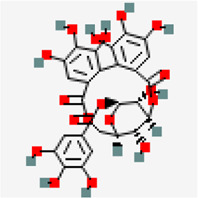	0.17	-0.15/soluble	*Terminalia bellirica, Terminalia chebula*
	−7.8	Lupeol acetate (ZINC4097722)	92,157	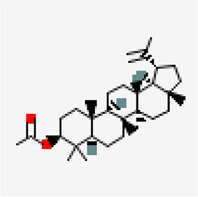	0.55	-7.35/poorly soluble	*Pedalium murex*
	−7.6	Emetine (ZINC000003830747) (DB13393)	10,219	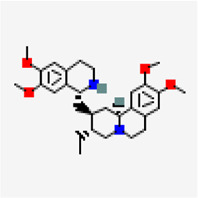	0.55	-7.7/poorly soluble	FDA approved drug
	−7.6	Chlorogenin (ZINC84668707)	12,303,065	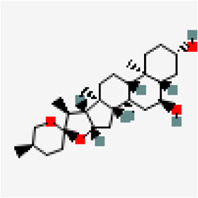	0.55	-3.69/soluble	*Solanum torvum*
	−7.6	Spirostan-3-ol (ZINC253504500)	14,492,795	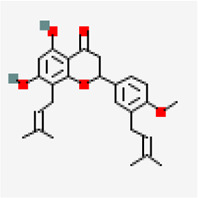	0.55	-6.56/poorly soluble	*Solanum nigrum*
	−7.6	5,7-Dihydroxy-4'-methoxy-8.3'-di-C-prenylflavanone	3,035,446	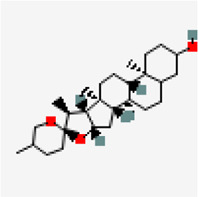	0.55	-4.51/moderately soluble	*Azadirachta indica*
Spike glycoprotein (PDB ID: 6M0J)	−8.2	1,8-Dichloro-9,10-diphenylanthracene-9,10-diol (ZINC5438671)	320,066	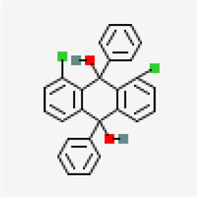	0.55	-10.45/insoluble	*Carica papaya*
	−8.2	Agathisflavone (ZINC4098505)	5,281,599	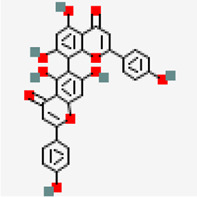	0.17	-8.7/poorly soluble	*Anacardium occidentale*
	−8.2	Amentoflavone (ZINC3984030)	5,281,600	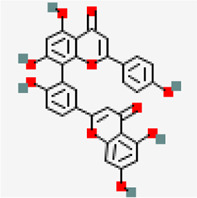	0.17	-8.7/poorly soluble	*Mangifera indica*
	−8.2	Ivermectin (ZINC238808778) (DB00602)	6,427,057	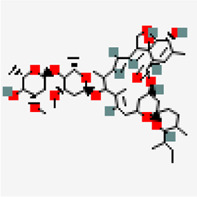	0.17	-3.89/soluble	FDA approved drug
	−8.1	3 o caffeoyltormentic acid	44,584,640	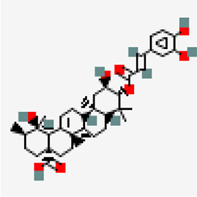	0.56	-5.99/moderately soluble	Antiviral compound
	−7.5	Agnuside (ZINC4098330)	442,416	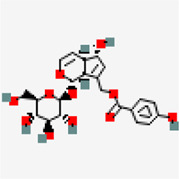	0.17	0.66/soluble	*Vitex negundo*
	−7.4	1,8-Dichloro-9,10-diphenylanthracene-9,10-diol (ZINC5438671)	320,066	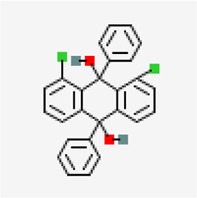	0.55	-10.45/insoluble	*Cissus quadrangularis*
	−7.3	Taraxerol (ZINC4082498)	92,097	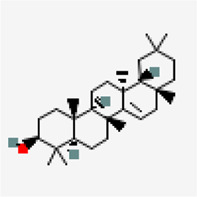	0.55	-7.16/poorly soluble	*Azadirachta indica*
	−7.3	Nimbinene	44,715,635	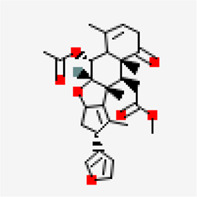	0.55	-5.66/moderately soluble	Antiviral compound
	−7.3	β-amyrin (ZINC13298265)	92,156	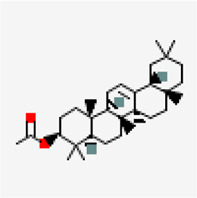	0.55	-7.77/poorly soluble	*Cissus quadrangularis*

#### Small Molecules/Herbal Compounds Exhibiting Significant Binding Affinity Against Main Protease

Despite of significant structural (RMSD: 0.71 Å) and binding site volume similarity of M^Pro^ between SARS-CoV (PDB ID: 2GZ9) and SARS-CoV-2 (PDB ID: 5R81), they showed differential binding affinity against different inhibitors (Table 1). Virtual screening of small molecules against M^Pro^ identified agathisflavone as the best hit exhibiting the binding affinity value of −8.2 kcal/mol.

Out of 744 ligands screened against SARS-CoV main protease, a ligand namely rutin abundantly found in *Terminalia chebula*, *Azadirachta indica* and *Ocimum basilicum* exhibited highest binding affinity value of −9.0 kcal/mol. In case of MERS-CoV main protease (PDB ID: 5C3N), amentoflavone predominantly found in *Mangifera indica* and *Garcinia species* showed the maximum binding affinity value of -8.6 kcal/mol. Interestingly, a cytotoxic biflavonoid agathisflavone found in cashew nut (*Anacardium occidentale*) was shown to exhibit significant binding affinity with −8.0 kcal/mol against the SARS-CoV-2 M^Pro^ (Supplementary Table S3). Agathisflavone is a biflavanoid derived from plant source and has been found to possess several biological activities (De Amorim et al., 2018). Various studies have found that agathisflavone possesses antioxidant, anti-inflammatory, antiviral, antiparasitic, cytotoxic, neuroprotective, and hepatoprotective activities. It has also been suggested that agathisflavone could be used in the treatment of oxidative stress, inflammatory diseases, microbial infection, hepatic and neurological diseases and cancer (Islam et al., 2019). Also, Agathisflavones have been reported for their cytotoxicity against malignant cell lines in earlier studies (Konan et al., 2012). This compound was found to involve in the formation of three hydrogen bonds with residues such as ASP 187, PRO 52 and ARG 40. This was followed by Rubusic acid (−8.1 kcal/mol) (*Pedalium murex*), solanocapsine (−7.9 kcal/mol)) (*Solanum nigrum*), chlorogenin (−7.7 kcal/mol) (*Solanum torvum*), Lupeol (−7.7 kcal/mol) (*Carica papaya* and *Azadirachta indica*), Cyanin (−7.7 kcal/mol) (*Zingiber officinale*), 3-O-trans-cafeoyltormentic acid (−7.7 kcal/mol) (antiviral compound), Luteolin-7-O-(6″-malonylglucoside) (−7.7 kcal/mol) (*Vitex negundo*), Agnuside (−7.6 kcal/mol) (*Vitex negundo*) and Luteolin 7-O-beta-D-glucoside (−7.6 kcal/mol) (*Vitex negundo*) exhibiting significant binding affinity in the decreasing order against M^Pro^.

**FIGURE 3:**
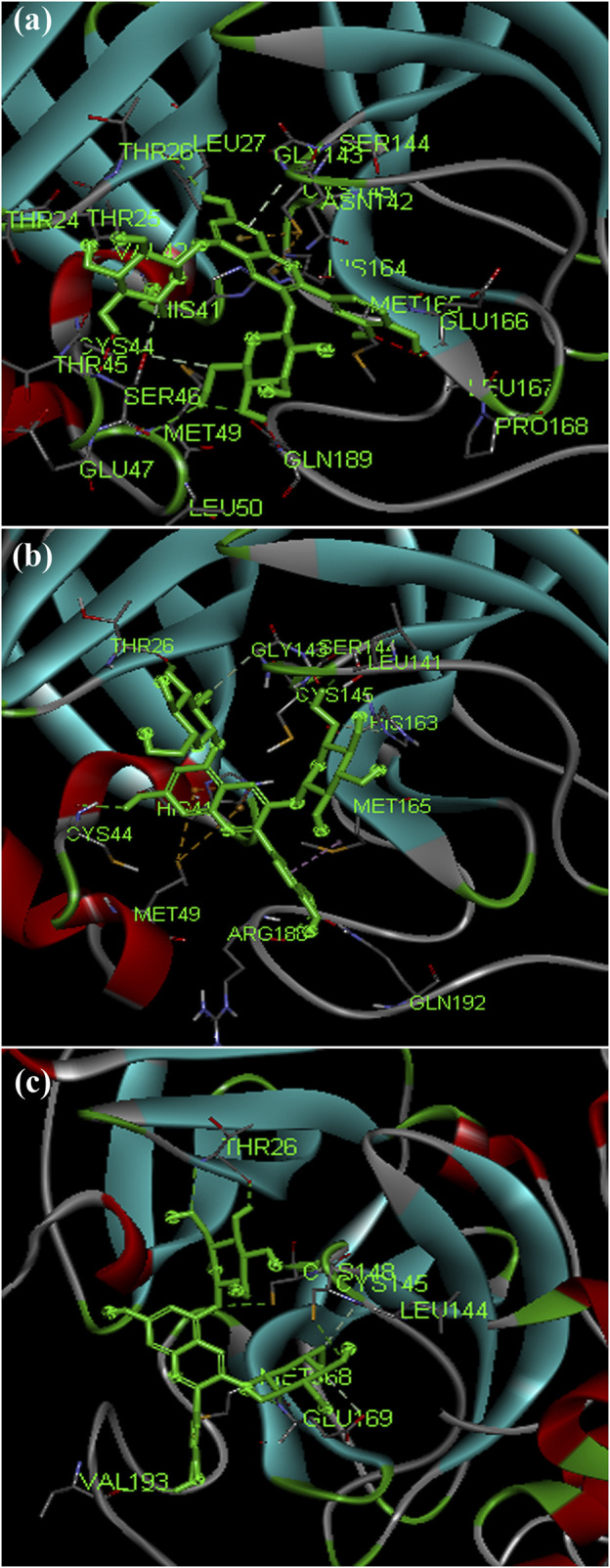
Complex structure of Cyanin with **(A)** SARS-CoV-2 M^Pro^ (PDB ID: 5R81) **(B)** SARS-CoV M^Pro^ (PDB ID: 2GZ9) **(C)** MERS-CoVM^Pro^ (PDB ID: 5C3N).

The phytochemical cyanin found in *Zingiber officinale* was found to have higher binding affinity with the main proteases of all three coronaviruses (Figures 3A–C) and placed in the top 10 hits list. It showed a binding affinity value of −8.3 kcal/mol, −8.2 kcal/mol and −7.7 kcal/mol against SARS-CoV-2(PDB ID: 5R81), SARS CoV (PDB ID: 2GZ9) and MERS CoV (PDB ID: 5C3N) main proteases respectively. Hydrogen bond interactions for SARS-CoV-2 (THR 26, SER 46 and GLU 166), SARS CoV (THR 26, LEU 141 and CYS 145) and MERS CoV (THR 26, CYS 149 and GLU 169) were observed in the binding sites respectively.

#### Effect of Suggested FDA Drugs on SARS-CoV-2 Protein Targets

**FIGURE 4:**
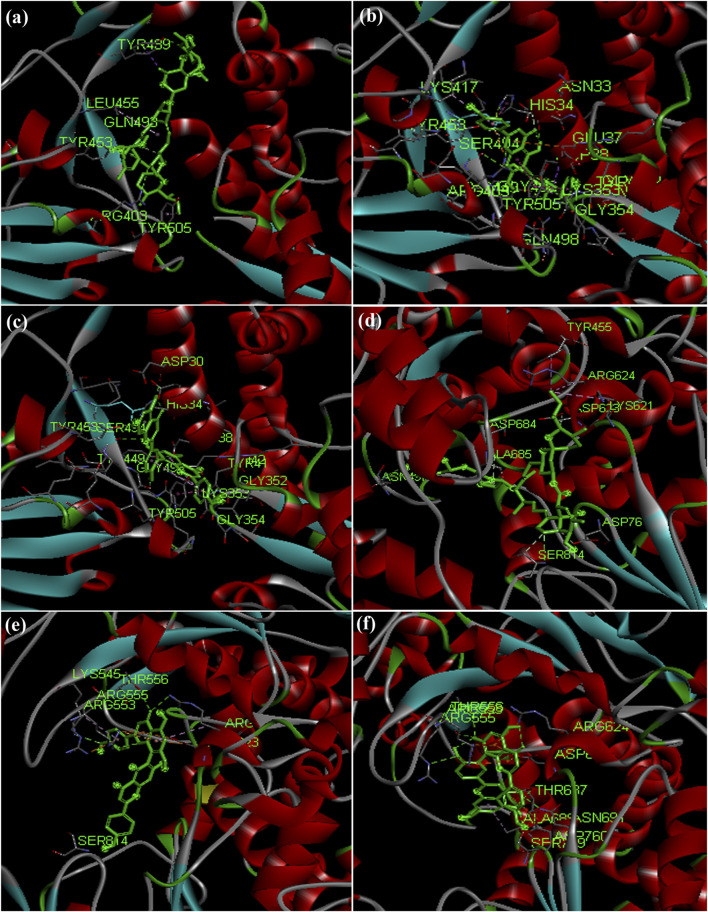
Docked complex structures of spike protein with **(A)** ivermectin, **(B)** agasthisflavone, **(C)** amentoflavone, and RdRp with **(D)** ivermectin **(E)** agasthisflavone **(F)** amentoflavone.

**FIGURE 5:**
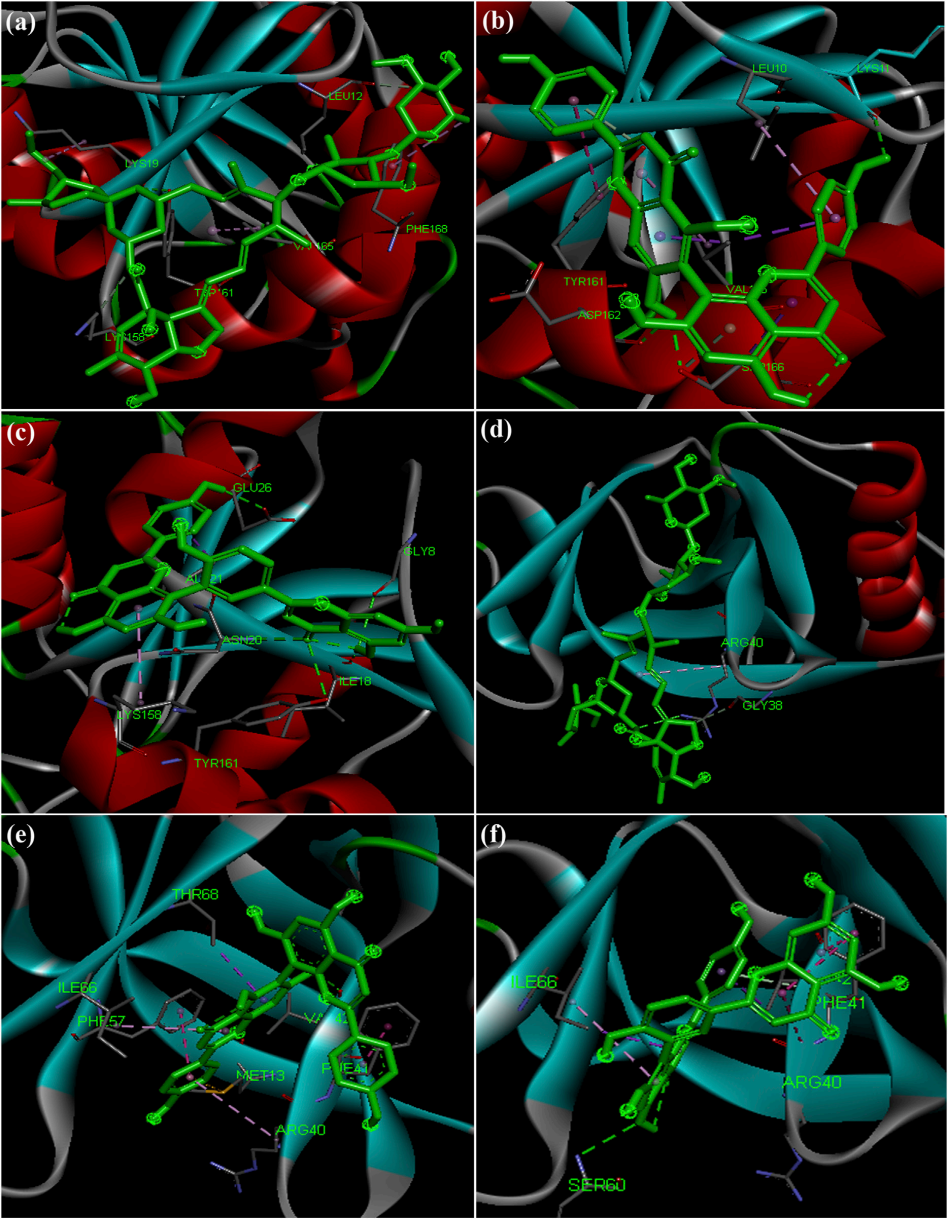
Docked complex structures of NSP3 protein with **(A)** ivermectin **(B)** agasthisflavone **(C)** amentoflavone, and NSP9 with **(D)** ivermectin **(E)** agasthisflavone **(F)** amentoflavone.

Hydroxycholoroquine, chloroquine and ivermectin drugs were selected as positive controls to compare the binding interaction of phytochemicals for the assessment of anti-viral activity (Caly et al., 2020; Principi and Esposito, 2020). Hydroxychloroquine was reported to show promising inhibitory activity against nCOVID’19 spike protein (Gautret et al., 2020; Liu et al., 2020). Our results revealed that hydroxycholorquine and chloroquine showed less binding affinity against all the seven targets of nCOVID’19 compared to ivermectin (Table 4). Ivermectin exhibited significant binding affinity value of −9.4 kcal/mol and −8.2 kcal/mol against RNA - dependent RNA polymerase (RdRp) (PDB ID: 6M71) and spike protein (PDB ID: 6M0J) respectively (Figures 4A,D). Ivermection also exhibited significant binding affinity against NSP3 (PDB ID: 6W02) (-6.7 kcal/mol) and NSP9 (PDB ID: 6W4B) (-7.5 kcal/mol) (Figures 5A–D) (Supplementary Table S4). Analysis of hydrogen bond interaction with ivermectin, showed three hydrogen bonds for RdRp target (ARG 403, TYR 453 and TYR 489) and two hydrogen bonds for spike protein (ASN 497 and SER 814), each one hydrogen bond for NSP3 (TYR 161) and NSP9 (ARG 40) respectively. It is also important to note that the ivermectin was reported as best hits for the above mentioned targets compared to hydroxychloroquine and chloroquine against SARS-CoV-2 targets. In the following section, binding of ivermectin was compared with amentoflavone and agasthisflavone phytochemicals as they were predicted to inhibit multi-target SARS-CoV-2 proteins based on the docking score.

#### Spike Protein

**FIGURE 6:**
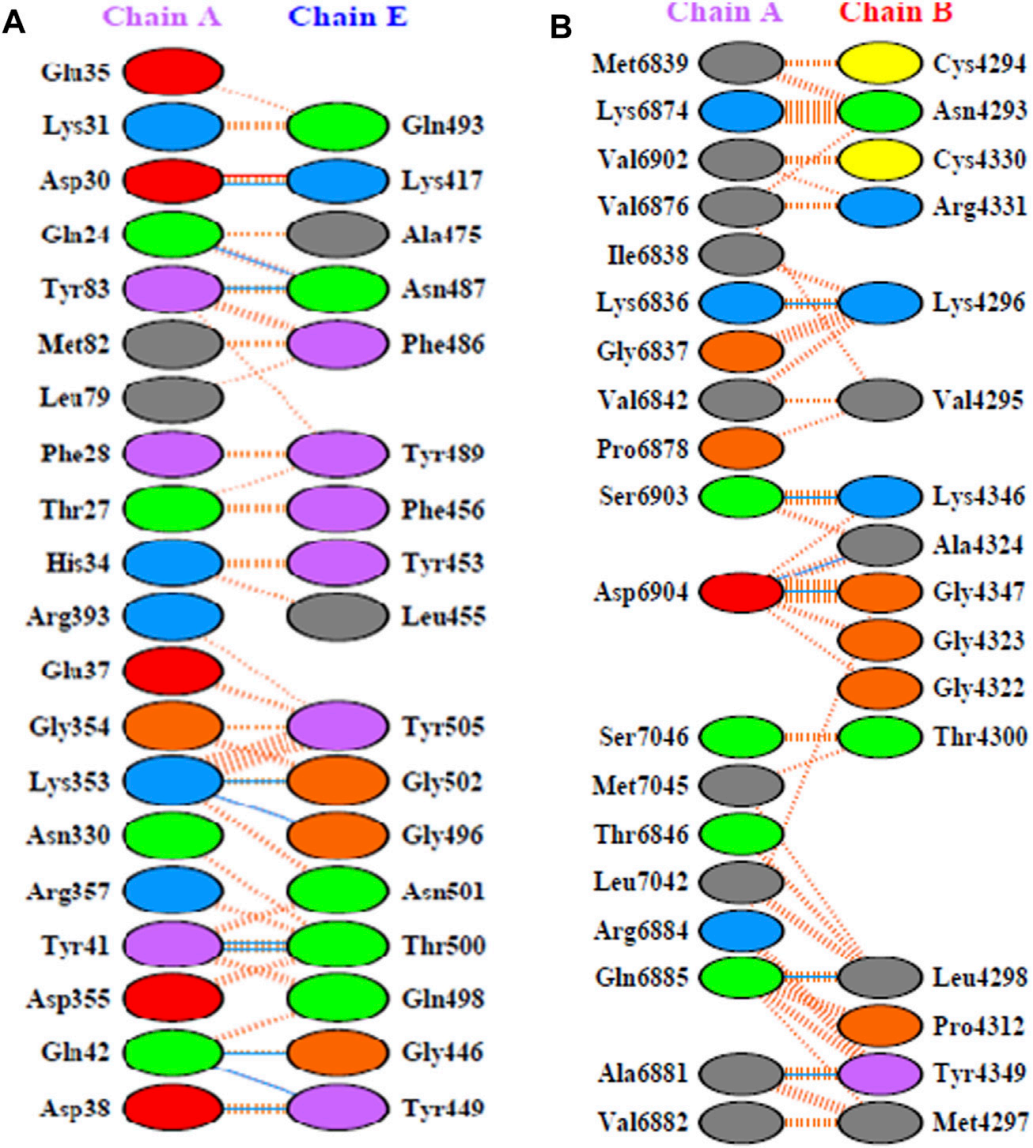
**(A)** PDB ID: 6M0J - Residue interaction between ACE-2 (chain A) and spike glycoprotein (chain E). **(B)** PDB ID: 6W4H - Interface of NSP10-NSP16 complex.

**FIGURE 7:**
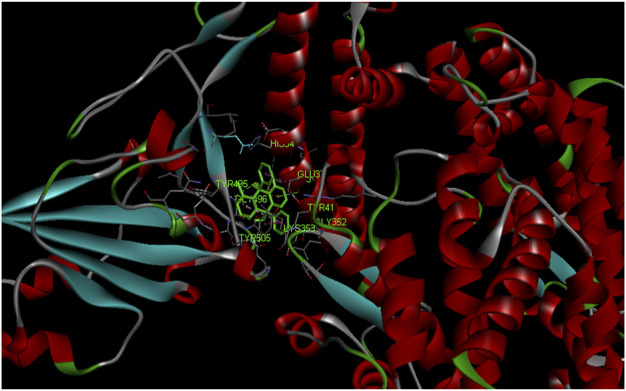
Spike protein (PDB ID: 6M0J) complex with 1,8-Dichloro-9,10-diphenylanthracene-9,10-diol (binding affinity: 8.2 kcal/mol).

X-ray crystal structure of spike glycoprotein (PDB ID: 6M0J) was chosen for performing virtual screening. Virtual screening was performed by choosing ACE interacting region as the binding site (Figure 6A). 1,8-Dichloro-9,10-diphenylanthracene-9,10-diol from *Carica papaya* was found to exhibit significant binding affinity against spike glycoprotein (−8.2 kcal/mol). GLY 496 residue was found to be involved in the formation of hydrogen bond with the 1, 8-Dichloro-9, 10-diphenylanthracene-9,10-diol. Earlier, leaf extracts of *Carica papaya* was reported to have significant effect in combating dengue virus infection (Rajapakse et al., 2019) and its exact role in increasing platelet counts is not clear. 1,8-Dichloro-9,10-diphenylanthracene-9,10-diol was found buried in the binding site of spike glycoprotein exhibiting hydrophobic interactions with residues such as LEU 39, TYR 41, TYR 449, TYR 453, TYR 495, PHE 497 and TYR 505 (Figure 7). This was followed by other small molecules *viz*., agasthisflavone (−8.2 kcal/mol) (Figure 4B), amentoflavone (−8.4 kcal/mol) (Figure 4C), ivermectin (−8.2 kcal/mol), three o caffeoyltormentic acid (−8.1 kcal/mol), agnuside (−7.5 kcal/mol) (*Vitex negundo*), taraxerol (−7.3 kcal/mol) (*Cissus quadrangularis*), nimbinene (−7.3 kcal/mol) (*Azadirachta indica*) and β-amyrin (−7.3 kcal/mol) (*Cissus quadrangularis*) exhibiting significantly higher level of binding affinity toward spike protein of nCOVID’19. Comparison of hydrogen bond interactions at the binding site for ivermectin (3 hydrogen bonds), amentoflavone (4 hydrogen bonds) and agasthisflavone (3 hydrogen bonds) showed higher number of interactions for amentoflavone involving residues TYR 449, SER 494, GLY 496 and ASP 30 respectively.

Residues are colored based on their physiochemical properties.

#### Ribonucleic Acid-dependent Ribonucleic Acid Polymerase (RdRp)

RdRp (PDB ID: 6M71) is responsible for replication of COVID-19 genome inside the host. Among the ligands tested, ivermectin (Figure 4D) showed the higher binding affinity value of −9.4 kcal/mol compared to the 744 small molecules screened in the present study. Amentoflavone stood at second position with the binding affinity value of −9.3 kcal/mol (Figure 4F). This was found to have interaction with three amino acid residues namely ARG 553, THR 556 and ASN 691. Ligands *viz*., Corilagin (−9.0 kcal/mol) (*Terminalia bellirica* and *Terminalia chebula*), Agasthisflavone (−8.9 kcal/mol) (*Anacardium occidentale*) (Figure 4E), 3-O-trans-caffeoyltormentic acid (−8.6 kcal/mol), Arjungenin (−8.2 kcal/mol) (*Terminalia chebula*), Crateogolic acid (−8.2 kcal/mol) (*Syzygium aromaticum*), 3.8’-biapigenin (−8.0 kcal/mol), cyanin (−7.9 kcal/mol) (*Zingiber officinale*) were found to exhibit significant binding affinity. Interaction analysis showed higher number of vander waals interactions for ivermectin compared to hydrogen bond interactions. Comparison of hydrogen bond interactions exhibited toward ivermectin (3 hydrogen bonds), agasthisflavone (4 hydrogen bond interactions) and amentoflavone (6 hydrogen bond interactions–ARG 553, ARG 555, THR 556, SER 759, ASN 691 and THR 687).

#### Non-structural Proteins

Apart from the four major structural proteins (S, E, M, and N proteins), non-structural proteins namely NSP3 (cleavage of N-terminal replicase poly protein) (PDB ID: 6W02), NSP9 (ssRNA binding) (PDB ID: 6W4B), NSP10-NSP16 (co-factor in activating replicating enzyme) (PDB ID: 6W4H) and NSP15 (PDB ID: 6W01) involved in the transcription and replication of nCoVID’19 can also serve as potential targets for containing the virus using inhibitory herbal molecules (Prajapat et al., 2020).

Virtual screening of small molecules against NSP3 identified amentoflavone (*Mangifera indica*) as the top scored ligand with binding affinity of −7.5 kcal/mol (Figure 5C). In the decreasing order of binding affinity, luteolin 7-O-(6''-malonylglucoside) (−7.1 kcal/mol) (*Vitex negundo*), rubusic acid (−6.8 kcal/mol) (*Pedalium murex*), acteoside (−6.7 kcal/mol) (*Clerodendrum serratum*), ivermectin (−6.7 kcal/mol), taraxerol acetate (−6.7 kcal/mol) (*Cissus_quadrangularis*), catechin 7-O-gallate (-6.6 kcal/mol), luteolin 7-O-beta-D-glucoside (−6.6 kcal/mol) (*Vitex negundo*), agathisflavone (−6.6 kcal/mol) (*Anacardium occidentale*) (Figure 5B) and luteolin-7-o-beta-d-glucopyranoside (−6.6 kcal/mol) (*Vitex negundo*) were found to have higher binding affinity next to amentoflavone*.* It was observed that three inhibitors from *Vitex negundo* were reported with the highest binding scores*. Vitex negundo* belongs to Verbenaceae family known for its effects for ailments like ophthalmia, deafness, indigestion, piles and jaundice (Venkateswarlu, 2012). Results of earlier experiments conducted by Wu et al. (2020) also revealed similar findings of vitexin from *Vitex negundo* exhibiting significant binding affinity toward NSP3.

Another small molecule, friedelin from *Vitex negundo* and *Acorus calamus* was also found to exhibit significant binding affinity of −9.6 kcal/mol against NSP9 (PDB ID: 6W4B) (Table 5). Even though, many hydrophobic interactions were observed, no hydrogen bond interaction was found in the binding site of NSP9. It is very interesting to observe that five out of the top ten inhibitors are from a single plant source *Solanum nigrum.* As evidenced from other studies, *Solanum nigrum* is one of the traditionally known medicinal plants known for its use in treatment ofseizure, pain, ulcer, inflammation, diarrhea, eye infections, jaundice and oxidative stresses (Jain et al., 2011; Javed et al., 2011; Wang et al., 2013; Zaidi et al., 2014).

Virtual screening against NSP15 (PDB ID:6W01) identified oleonolic acid and urosolic acid as molecules exhibiting highest binding affinity values of −9.2 kcal/mol. Oleonolic acid and urosolic acid are known for their anti-cancerous and anti-inflammatory activities (Arshad Qamar et al., 2010; Kashyap et al., 2016; Yu et al., 2017). Both the phytochemicals are reported to be enriched in *Ocimum basilicum* and *Ocimum tenuiflorum* (Table 5). This was followed by crategolic acid (−8.9 kcal/mol) (*Syzygium aromaticum*), arjungenin (−8.8 kcal/mol) (*Terminalia chebula*), hederagenin (−8.7 kcal/mol) (*Nigella sativa*), triterpenoid (−8.6 kcal/mol) (*Abutilon indicum*)*,* beta-amyrin (−8.6 kcal/mol) (*Cissus quadrangularis*)*,* friedelin (−8.6 kcal/mol) (*Vitex negundo, Acorus calamus*)*,* catechin 7-O-gallate (−8.5 kcal/mol) (*Camellia sinensis*) and arjunolic acid (−8.5 kcal/mol) (*Terminalia chebula*)*.*

In the case of NSP10-NSP16 (PDB ID: 6W4H) protein complex, interface of the complex (Figure 6B) was chosen as the binding site for performing virtual screening. Compounds such as amentoflavone (−8.5 kcal/mol), 10-methoxycamptothecin (−8.3 kcal/mol), 3.8'-biapigenin (−8.2 kcal/mol), taraxerol acetate (−8.0 kcal/mol) (*Cissus_quadrangularis*), corilagin (−7.8 kcal/mol) (*Terminalia bellirica* and *Terminalia chebula*), lupeol acetate (−7.8 kcal/mol) (*Pedalium murex*), emetine (−7.6 kcal/mol), chlorogenin (−7.6 kcal/mol) (*Solanum torvum*), spirostan-3-ol (−7.6 kcal/mol) (*Solanum torvum*) and 5,7-Dihydroxy-4'-methoxy-8.3'-di-C-prenylflavanone (−7.6 kcal/mol) (*Azadirachta indica*) were ranked among the top 10 molecules exhibiting highest binding affinity.

### Molecules Exhibiting Inhibitory Activity Against Multiple Protein Targets of nCOVID’19

Phytochemicals showing strong interactions against multiple targets of viruses are expected to confer durable protection to the patients. This will be more beneficial in situations where the virus is developing mutations in one of the targets. Small molecules namely, amentoflavone, agathisflavone, catechin-o-gallate and chlorogenin exhibited significant binding affinity toward multiple targets of nCOVID’19.

Amentoflavone showed docking score of RdRp (−9.3 kcal/mol) (Figure 4C), NSP9 (−8.3 kcal/mol) (Figure 5F), NSP3 (−7.4 kcal/mol) (Figure 5C), NSP10-NSP16 (−8.5 kcal/mol) and spike glycoprotein (−8.2 kcal/mol). In all the docked complexes, the target - ligand binding affinity was greater than (−8.0 kcal/mol) except NSP3. 3D and 2D Protein-ligand interactions exhibited by the small molecules amentoflavone and agathisflavone are shown in Figure 4, Figure 5 and Supplementary Table S3. Molecular interactions such as hydrogen bond, vander waals interaction, pi interaction were observed to be higher for amentoflavone incomparison with the drug ivermectin. Amentoflavone is a naturally occurring biflavonoid reported to be found in more than 120 plants (Yu et al., 2017). Many of these plants have been used in traditional medicine for several thousand years in different parts of the world. Several studies have reported that amentoflavone possess anti-inflammatory, anti-oxidative, anti-diabetic, anti-tumor, anti-viral and anti-fungal activities (Yu et al., 2017). Evidences have been reported for amentoflavone exhibiting anti-senescence activity in the cardiovascular and central nervous system (Park et al., 2011). Further, Amentoflavone isolated from *Torreya nucifera* was demonstrated to possess inhibitory activity against SARS-CoV3CL^Pro^ (Ryu et al., 2010).

Similarly, agathisflavone was found to exhibit significant interaction against four different protein targets viz., RNA-dependent RNA polymerase (−8.9 kcal/mol), SARS-CoV-2 main protease (−8.2 kcal/mol), spike glycoprotein (−8.2 kcal/mol) and NSP 3 (−6.6 kcal/mol) (Supplementary Table. S3). Catechin-o-gallate was also found to possess significant binding affinity toward spike glycoprotein (−7.3 kcal/mol), NSP3 (−6.6 kcal/mol), RNA-dependent RNA polymerase (−7.9 kcal/mol) and NSP15 (−8.5 kcal/mol). Role of catechins for antiviral activity against the number of viruses due to the increased affinity for cellular membranes has been reported in many articles (Kaihatsu et al., 2018). Chlorogenin from *Solanum torvum* exhibited binding affinity value of −8.2 kcal/mol (NSP9), −7.7 kcal/mol (M^Pro^) and −7.6 kcal mol (NSP16-NSP10) of SARS-CoV-2. *Solanum torvum* is well known for its pharmacological properties for the treatment of human ailments and its phytoconstituent chlorogenin also has antiviral effect on influenza viruses (Agrawal et al., 2010; Lim, 2013; Shi et al., 2020).

**FIGURE 8:**
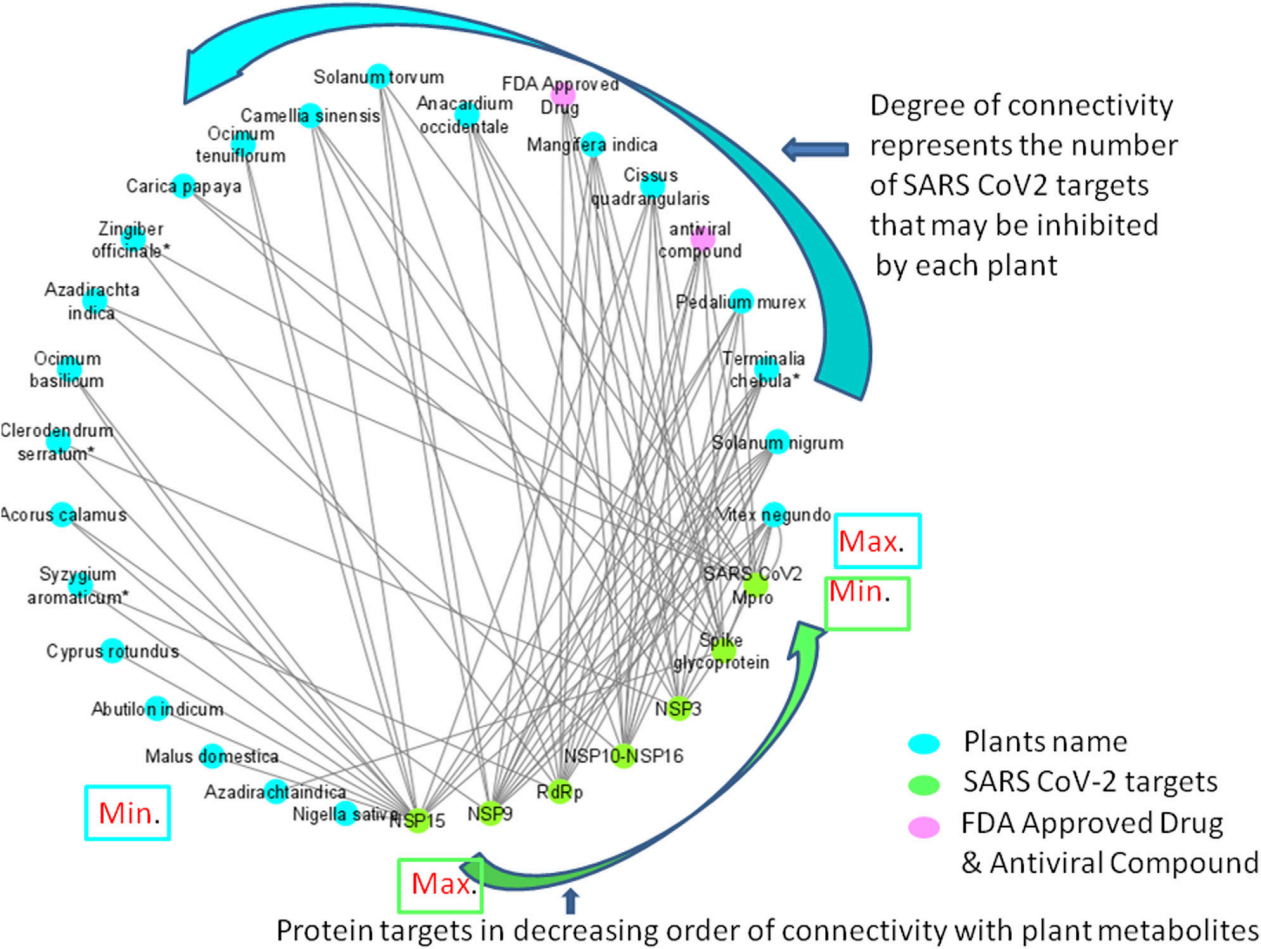
Network diagram showing the interaction of plant phytochemcials with SARS-CoV-2 protein targets. Degree of connectivity represents number of SARS-CoV-2 that may be inhibited by each plant. In this network, NSP 15 stands first in the order where many plants connected (degree of connectivity) which indicates that metabolites from the connected plants has shown highest binding affinity (top ten screened compounds). Likewise, *Vitex negundo* was predicted to inhibit highest number of SARS-CoV-2 targets in the virtual screening. Plants name with * indicates their presence in Kabasura kudineer.

Fifteen different compounds *viz*., 3.8'-biapigenin, 3-O-trans-caffeoyltormentic acid, agnuside, arjungenin, corilagin, crategolic acid, cyanin, friedelin, luteolin 7-O-(6''-, malonylglucoside), N-methylsolasodine, rubusic acid, solanocapsine, spirostan-3-ol, taraxerol and taraxerol acetate were found to possess significant interactions with at least two protein targets of SARS-CoV-2 (Table 6) (Figure 8). Among the above reported 15 compounds, cyanin targeted all the three Coronavirus MP^ro^. 2D representation of MP^ro^-cyanin interaction of all the coronaviruses is provided in the Supplementary Table S5.

**TABLE 6:** Phytochemicals having strong interaction based on the top 10 hits screened for SARS-CoV-2targets.

S. No	Compound name	Plant name	Protein targets
	Acetoside	*Clerodendrum serratum* *^a^*	NSP3
	Agathisflavone	*Anacardium occidentale*	SARS-CoV-2M^pro^
			RdRp
			NSP3
			Spike glycoprotein
	Agnuside	*Vitex negundo*	SARS-CoV-2M^pro^
			Spike glycoprotein
	Amentoflavone	*Mangifera indica*	RdRp
			NSP9
			NSP3
			NSP10-NSP16
			Spike glycoprotein
	Arjungenin	*Terminalia chebula* *^a^*	RdRp
			NSP15
	Arjunolic acid	*Terminalia chebula* *^a^*	NSP15
	Beta-amyrin	*Cissus quadrangularis*	NSP15, spike glycoprotein
	Catechin 7-O-gallate	*Camellia sinensis*	RdRp
			NSP15
			NSP3
			Spike glycoprotein
	Chlorogenin	*Solanum torvum*	SARS-CoV-2M^pro^
			NSP9
			NSP10-NSP16
	Corilagin	*Terminalia bellirica* *^a^*, *Terminalia chebula* *^a^*	RdRp
			NSP10-NSP16
	Crategolic acid	*Syzygium aromaticum* *^a^*	NSP15
			RdRp
	Cyanin	*Zingiber officinale* *^a^*	SARS-CoV-2M^pro^
			RdRp
	Diosgenin	*Solanum nigrum, Pedalium murex*	NSP9
	Emetine	FDA approved drug	NSP10-NSP16
	Friedelin	*Vitex negundo, Acorus calamus*	NSP9
			NSP15
	Hederagenin	*Nigella sativa*	NSP15
	Ivermectin	FDA approved drug	RdRp
			NSP3
			Spike glycoprotein
	Lupeol	*Carica papaya *and *Azadirachta indica*	SARS-CoV-2M^pro^
	Lupeol acetate	*Pedalium murex*	NSP10-NSP16
	Luteolin 7-O-(6''-malonylglucoside)	*Vitex negundo*	SARS-CoV-2M^pro^
			NSP3
	Luteolin 7-O-beta-D-glucoside	*Vitex negundo*	SARS-CoV-2M^pro^
			NSP3
	Luteolin-7-o-beta-d-glucopyranoside	*Vitex negundo*	NSP3
	Nimbinene	*Azadirachtaindica*	Spike glycoprotein
	N-methylsolasodine	*Solanum nigrum*	NSP9
	Oleanolic acid	*Ocimum basilicum, Cyprus rotundus, Clerodendrum serratum* *^a^* *, Ocimum tenuiflorum*	NSP15
	Rubusic acid	*Pedalium murex*	SARS-CoV-2M^pro^
			NSP3
	Solanocapsine	*Solanum nigrum*	SARS-CoV-2M^pro^
			NSP9
	Solasodine	*Solanum nigrum, Solanum torvum*	NSP9
	Spirostan-3-ol	*Solanum nigrum*	NSP9
			NSP10-NSP16
	Taraxerol	*Cissus quadrangularis*	NSP9
			Spike glycoprotein
	Taraxerol acetate	*Cissus quadrangularis*	NSP3
			NSP10-NSP16
	Triterpenoid	*Abutilon indicum*	NSP15
	Ursolic-acid	*Ocimum basilicum, Pedalium murex, Malus domestica, Ocimum tenuiflorum*	NSP15
	5,7-Dihydroxy-4'-methoxy-8.3'-di-C-prenylflavanone	*Azadirachta indica*	NSP10-NSP16
	1,8-Dichloro-9,10-diphenylanthracene-9,10-diol	*Carica papaya*	Spike glycoprotein
	3 O caffeoyltormentic acid	Antiviral compound	Spike glycoprotein
	10-Methoxycamptothecin	Antiviral compound	NSP10-NSP16
	3.8'-biapigenin	Antiviral compounds	RdRp
			NSP10-NSP16
	3-O-trans-caffeoyltormentic acid	Antiviral compound	SARSCoV-2 M^pro^
			RdRp

a Kabasura kudineer-plant compounds.

## Discussion

Among the 605 phytochemicals originating from 37 plant species, 33 (6% approx.) phytochemicals from 22 plants (Figure 8) were found to be the best hits with higher binding affinities against all the seven targets (Table 5). Among those 22 plants, four plants were found to be the ingredients of a traditional siddha herbal formulation namely “Kabasura kudineer” recommended by AYUSH Board of Government of India for boosting immunity. *Vitex negundo* was reported to possess 32 different phytochemicals that were included in this study. Out of the 33, five different compounds namely, luteolin 7-O-beta-D-glucoside, luteolin 7-O-(6'-malonylglucoside), agnuside, luteolin-7-o-beta-d-glucopyranoside and friedelin were found to exhibit significant binding affinity against five different protein targets of SARS-CoV-2 namely, spike glycoprotein, SARS-CoV-2 main protease, NSP3, NSP9, NSP15 in the SARS-CoV-2.

Solanocapsine, Spirostan-3-ol, N-methylsolasodine, Diosgenin and Solasodine are the phytochemicals reported in *Solanum nigrum* which effectively inhibited three SARS-CoV-2 targets (SARS-CoV-2M^Pro^, NSP9 and NSP16-NSP10).

*Pedalium murex* was reported to have Diosgenin (NSP9), Lupeol acetate (NSP16-NSP10), Urosolic acid (NSP15) and Rubusic acid (SARS-CoV-2 M^pro^, NSP3) which might be probable SARS-CoV-2inhibitors. It is noteworthy that maximum of five targets were predicted to be inhibited by the compounds from *Pedalium murex.* In spite of its role as anti-ulcerogenic, nephroprotective, hypolipidemic, aphrodisiac, anti-oxidant, anti-microbial and insecticidal activities, *Pedalium murex* has been traditionally used in treating ailments like cough and cold as a regular practice (Patel et al., 2011a).

Medicinal plants namely, *Azadirachta indica, Terminalia chebula, Cissus quadrangularis, Clerodendrum serratum* and *Ocimum basilicum* reported more than two phytochemicals that might possibly inhibit SARS-CoV-2 targets. These herbal plants might be the potential targets for future research toward developing herbal formulations against SARS-CoV-2. Intensive genomics and proteomics research may lead to identification of novel drugs against this pandemic disease.

Knowledge of the phytochemical composition in the reported plants is important to understand the role of bioactive molecule in the traditional medicine. Phytochemical composition of the some of the screened plants quantified using various analytical techniques are given as follows: Cyanin (*Zingiber officinale*) (20%) (Ghasemzadeh et al., 2010)*,* amentoflavone (*Mangifera indica*) (90.92 ± 4.91 mg/100 g) (Ma et al., 2011)*,* agathisflavone (*Anacardium occidentale*) (4.39 ± 0.01 (mg/g)) (Onuh et al., 2017), Corilagin (*Terminalia chebula*) (13.05 mg/g) (Juang et al., 2004) luteolin derivatives (*Vitex negundo*) (123.776 – 127.774 mg/g) (Koirala et al., 2020), Solanocapsine (*Solanum nigrum*) (>50%) (Almoulah et al., 2017), Solasodine (*Solanum nigrum*) (75.89%) (Jasim et al., 2015), Diosgenin (*Pedalium murex*) (0.06%) (Patel et al., 2011a), Quercetrin (*Azadirachta indica*) (0.016%) (Sarkar et al., 2020), Rutin (*Azadirachta indica*) (0.14%) (Sarkar et al., 2020), Nimbinene (*Azadirachta indica*) (1.2%) (Sadeghian and Mortazaienezhad, 2007), Rutin (*Terminalia chebula*) *(0.17%)* (Kumar et al., 2010)*,* Rutin (*Ocimum basilicum*) (425.71 ± 2.15 μg/g) (Vlase et al., 2014) and Isoquercetin (*Ocimum basilicum*) (179.19 ± 1.93 μg/g) (Vlase et al., 2014).

Antiviral activity of the plants highlighted in this study, whose phytochemicals are predicted to have inhibitory effect on SARS-CoV-2 targets have proven antiviral role on many other human pathogenic viruses. Antiviral activity of *Vitex negundo L.* against Chikungunya virus and HIV-1 Reverse transcriptase (RT) have been previously reported by Kothandan and Swaminathan (2014) and Nair and Ramachandran (2012) (Nair, 2012; Kothandan and Swaminathan, 2014). *Solanum nigrum* was reported to have antiviral effect against Hepatitis C Virus chronic infection (Javed et al., 2011). Similarily, the antiviral and virucidal effect of neem leaves was studied regarding its activity and possible mechanism of action against Coxsackie B group of viruses (Badam et al., 1999). Sivagami and Sailaja 2021 (Sivagami and Sailaja, 2021) have reported the antiviral activity of *Clerodendrum serratum* against yellow fever virus. Likewise, antiviral effect of *Terminalia chebula* on swine influenza A virus infection has also been proved (Ma et al., 2010). Phytochemicals like apigenin and ursolic acid from *Ocimum basilicum* has shown broad-spectrum of antiviral activity against many of the DNA and RNA viruses (Chiang et al., 2005). *Zingiber officinale* has been proved to have antiviral activity against human respiratory syncytial virus (Chang et al., 2013). Also, the plants like *Mangifera indica* and *Anacardium occidentale* L. inhibits Herpes simplex virus two and influenza virus neuraminidase respectively (Parvez, 2016; de Freitas et al., 2020).

ADME properties are important to understand the pharmacokinetics and physio-chemical properties of phytochemicals which further provides insight on drug design and formulation research. Analysis on solubility and bioavailability based on the SWISSADME prediction for the screened inhibitors showed different level of classifications as given in Supplementary Table S2. According to the Silicos-IT LogSw score, 41% of the screened ligands were classified soluble, 31% (moderately soluble), 27% (poorly soluble) and one molecule (1,8-Dichloro-9,10-diphenylanthracene-9,10-diol) alone as insoluble. Bioavailability score predicted on the probability of a compound to have at least 10% oral bioavailability (Martin, 2005) in rat has been reported in which one compound (uteolin 7-O-(6''-malonylglucoside)) has less score (0.11 score), 17 compounds have (0.17 score), 27 compounds have (0.55 score) and three compounds (ursolic acid, betulinic acid and oleanolic acid) with 0.85 score respectively.

## Conclusion

Generation of improved knowledge and understanding biochemical and molecular basis of herbals used in traditional Ayurveda and siddha medicine will accelerate development of effective drugs in controlling emerging diseases. In this study, comparative analysis of main proteases of MERS-CoV (PDB ID: 5C3N), SARS-CoV (PDB ID: 2GCZ9) and SARS-CoV-2 (PDB ID: 5R81) revealed significant differences between the three homologs which were confirmed by differential binding affinity exhibited by 744 phytochemicals/small molecules against the three main proteases. Cyanin from *Zingiber officinale* was screened as the best phytochemical with highest binding energy against main proteases of all the three viruses. Popular fruit tree, mango (*Mangifera indica*) and, cashew nut (*Anacardium occidentale*) rich in amentoflavone and agathisflavone were showing possible inhibitory activity against multiple targets of SARS-CoV-2. *Vitex negundo, Solanum nigrum, Pedalium Murex, Terminalia chebula, Azadirachta indica, Cissus quadrangularis, Clerodendrum serratum* and *Ocimum basilicum* were also found to contain phytochemicals that may have possible inhibitory activity against SARS-CoV-2 proteins. More interestingly, this study has picked up *Carica Papaya* that may possess inhibitory activity against spike glycoprotein and M^Pro^ of SARS-CoV-2 which was known for its protective role against dengue virus in humans (Rajapakse et al., 2019). Overall, this study has shortlisted potential phytochemicals that may have inhibitory activity against SARS-CoV-2 which could be taken for further testing, formulation and discovery of novel drugs.

## Data Availability

The datasets presented in this study can be found in online repositories. The names of the repository/repositories and accession number(s) can be found in the article/Supplementary Material.
