# Supplementary material for: Exploring Phytochemicals of Traditional Medicinal Plants Exhibiting Inhibitory Activity Against Main Protease, Spike Glycoprotein, RNA-dependent RNA Polymerase and Non-Structural Proteins of SARS-CoV-2 Through Virtual Screening
Source: Front Pharmacol. 2021 Jul 8;12:667704. doi: 10.3389/fphar.2021.667704 (PMC8295902; doi:10.3389/fphar.2021.667704)
Supplement: Supplementary file 2 [file Table1.DOCX]

**Supplementary Information:**

**Table S1: a) List of phytochemical Compounds**

| **S.no** | **Ligand Name** | **Plant Part** | **Plant Name** |  | **Pubchem ID** |
| --- | --- | --- | --- | --- | --- |
|  | (+)-alpha-phellandrene | Root | *Curcuma longa (Turmeric)* |  | *443160* |
|  | (+)-sabinene | Root | *Curcuma longa (Turmeric)* |  | *10887911* |
|  | (1S,5S)-1-isopropyl-4-methylenebicyclo[3.1.0]hexane | Flower, Root | *Piper longum (Thippili)* |  | 11051711 |
|  | (2R,4aR,8aR)-2-methyldecalin | Flower, Root | *Piper longum (Thippili)* |  | 12816526 |
|  | (9R,10R)-1,5-Dichloro-9,10-diphenylanthracene-9,10-diol | leaves, fruit | *carica papaya (Papaya)* |  | 40736904 |
|  | (E)-2-[[4-(2-Imidazol-1-ylethoxy)phenyl]methyl]-3-propan-2-ylbut-2-enedioic acid | leaves, fruit | *carica papaya (Papaya)* |  | 90184972 |
|  | (E)-5-(4-hydroxy-3-methoxy-phenyl)-1-piperidino-pent-2-en-1-one | Flower, Root | *Piper longum (Thippili)* |  | 11630663 |
|  | (E,E,E)-11-(1,3-Benzodioxol-5-yl)-N-(2-methylpropyl)-2,4,10-undecatrienenamide | Flower, Root | *Piper longum (Thippili)* |  | 6453083 |
|  | (R)-N-(1'-methoxycarbonyl-2'-phenylethyl)-4-hydroxybenzamide | Root | *Abutilon indicum (Thuthi)* |  | 73941365 |
|  | (Z)-caryophyllene | Flower, Root | *Piper longum (Thippili)* |  | 6429301 |
|  | 1-(2,4-decadienoyl)-pyrrolidine | fruit | *Pepper nigrum (Pepper)* |  | *46935132* |
|  | 1-(2,4-dodecadienoyl)-pyrrolidine | fruit | *Pepper nigrum (Pepper)* |  | *10999431* |
|  | 1,4-cadinadiene | Flower, Root | *Piper longum (Thippili)* |  | 50986185 |
|  | 1-(4-hydroxy-3-methoxyphenyl)-3,5-diacetoxyoctane | Rhizome | *Zingiber officinale (Chukku)* |  | 53380863 |
|  | 1(7),2-p-menthadien-6-ol | fruit | *Pepper nigrum (Pepper)* |  | *5319361* |
|  | 1,4,7,-Cycloundecatriene, 1,5,9,9-tetramethyl-, Z,Z,Z- | Flower, Root | *Piper longum (Thippili)* |  | 5368784 |
|  | 1,8-cineole | Leaf | *Ocimum basilicum (Thiruneetrupachai)* |  | 2758 |
|  |  | Rhizome | *Acorus calamus ( Vasambu)* |  | [2758](https://pubchem.ncbi.nlm.nih.gov/compound/2758) |
|  |  | Rhizome | *Zingiber officinale (Chukku)* |  | 2758 |
|  |  | Root | *Cyprus rotundus (Korai kilangu)* |  | 2758 |
|  |  | Root | *Curcuma longa (Turmeric)* |  | 2758 |
|  | (1R,5R,7S)-4,7-dimethyl-7-(4-methylpent-3-enyl)bicyclo[3.1.1]hept-3-ene | Flower, Root | *Piper longum (Thippili)* |  | 13889654 |
|  | 1-[(2E,4E)-1-oxo-2,4-hexadecadienyl]-piperidine | Flower, Root | *Piper longum (Thippili)* |  | 10980124 |
|  | (3S)-3,7-dimethylocta-1,6-dien-3-yl] propanoate | Flower, root | *Piper longum (Thippili)* |  | 1616358 |
|  | 10-dehydrogingerdione | Rhizome | *Zingiber officinale (Chukku)* |  | 51675187 |
|  | 10-gingediol | Rhizome | *Zingiber officinale (Chukku)* |  | 53702863 |
|  | 10-gingerdione | Rhizome | *Zingiber officinale (Chukku)* |  | 14440539 |
|  | 10-gingerol | Rhizome | *Zingiber officinale (Chukku)* |  | 168115 |
|  | 10-shogaol | Rhizome | *Zingiber officinale (Chukku)* |  | 6442612 |
|  | 14-deoxy-11-oxoandrographolide | plant | *Andrographis paniculata (Nilavembu)* |  | 101593061 |
|  | 1-alpha-phellandrene | fruit | *Pepper nigrum (Pepper)* |  | *7460* |
|  | 1-terpinen-4-ol | fruit | *Pepper nigrum (Pepper)* |  | *11230* |
|  | 2,2-Dimethoxybutane | Flower, Root | *Piper longum (Thippili)* |  | 137941 |
|  | 2,4,5-trimethoxybenzaldehyde | Rhizome | *Acorus calamus ( Vasambu)* |  | [20525](https://pubchem.ncbi.nlm.nih.gov/compound/20525) |
|  | 2',4-dihydroxychalcone-4-glucoside | Flower | *Justicia adhatoda (Aada thoda) (Aada thoda)* |  | 6857762 |
|  | 2,4-Dihydroxycinnamic acid | Plant | *Solanum nigrum ( Manathakkali)* |  | *446611* |
|  | 2,4-Dimethoxytoluene | Flower, Root | *Piper longum (Thippili)* |  | 96403 |
|  | 2,5-dimethoxybenzoquinone | Rhizome | *Acorus calamus ( Vasambu)* |  | [101405](https://pubchem.ncbi.nlm.nih.gov/compound/101405) |
|  | 2,8-p-menthadien-1-ol | fruit | *Pepper nigrum (Pepper)* |  | *155626* |
|  | 2-[(4R)-6-Fluoro-2,2,4-trimethyl-3,4-dihydroquinolin-1-yl]acetohydrazide | leaves, fruit | *carica papaya (Papaya)* |  | 100207507 |
|  | 2-[(E)-4(4-Chlorophenyl)-2Butenyl]malonic aciddiethyl ester | leaves, fruit | *carica papaya (Papaya)* |  | 4594935 |
|  | 2-6-dimethyl-hept-5-en-1-al | Rhizome | *Zingiber officinale (Chukku)* |  | 61016 |
|  | 2-6-dimethyl-octa-2-6-diene-1-8-diol | Rhizome | *Zingiber officinale (Chukku)* |  | 5363397 |
|  | 2-Aminohexanedioic acid | Plant | *Solanum nigrum ( Manathakkali)* |  | *469* |
|  | 2-heptyl-acetate | Flower | *Syzygium aromaticum (Lavangam)* |  | 71410716 |
|  | 2-Hydroxy myristic acid | Flower, Root | *Piper longum (Thippili)* |  | 1563 |
|  | 2-nonyl-acetate | Flower | *Syzygium aromaticum (Lavangam)* |  | 85788 |
|  | 2-Nonynoic acid | Flower, Root | *Piper longum (Thippili)* |  | 61451 |
|  | 2-Phenylethanol | Flower, Root | *Piper longum (Thippili)* |  | 6054 |
|  | 3-O-caffeoyl-D-quinic acid | Plant | *Solanum nigrum ( Manathakkali)* |  | *1794426* |
|  | 3-octanone | Leaf | *Ocimum basilicum (Thiruneetrupachai)* |  | 246728 |
|  | 3-phenyl-benzaldehyde | Rhizome | *Zingiber officinale (Chukku)* |  | 121053 |
|  | 3-phenylundecane | Flower, Root | *Piper longum (Thippili)* |  | 20655 |
|  | 4,4ʹʹ- dimethoxy-trans-stilbene | Leaves | *Vitex negundo (Nochi)* |  | 641296 |
|  | 4-[(1-Carboxy-2-methylbutyl)amino]-2(1H)-pyrimidinone | Flower, Root | *Piper longum (Thippili)* |  | 591989 |
|  | 4-gingerol | Rhizome | *Zingiber officinale (Chukku)* |  | 46901319 |
|  | 4-phenyl-benzaldehyde | Rhizome | *Zingiber officinale (Chukku)* |  | 76689 |
|  | 4-terpineol | Rhizome | *Zingiber officinale (Chukku)* |  | 11230 |
|  | 5beta-Pregnane | Plant | *Solanum nigrum ( Manathakkali)* |  | *439513* |
|  | 5-Caffeoylquinic acid | Plant | *Solanum nigrum ( Manathakkali)* |  | *1794427* |
|  | 5-Hydroxy-2',3',7,8- tetramethoxyflavone | plant | *Andrographis paniculata (Nilavembu)* |  | 5319878 |
|  | 6-dehydrogingerdione | Rhizome | *Zingiber officinale (Chukku)* |  | 22321203 |
|  | 6-gingediol | Rhizome | *Zingiber officinale (Chukku)* |  | 101660275 |
|  | 6-gingerdiol | Rhizome | *Zingiber officinale (Chukku)* |  | 11369949 |
|  | 6-gingerdione | Rhizome | *Zingiber officinale (Chukku)* |  | 162952 |
|  | 6-gingerol | Rhizome | *Zingiber officinale (Chukku)* |  | 442793 |
|  | 6-gingesulfonic-acid | Rhizome | *Zingiber officinale (Chukku)* |  | 126890 |
|  | 6-methylgingediacetate | Rhizome | *Zingiber officinale (Chukku)* |  | 53145002 |
|  | 6-methylgingediol | Rhizome | *Zingiber officinale (Chukku)* |  | 80179394 |
|  | 6-methyl-hept-5-en-2-ol | Rhizome | *Zingiber officinale (Chukku)* |  | 20745 |
|  | 6-methyl-hept-5-en-2-one | Rhizome | *Zingiber officinale (Chukku)* |  | 9862 |
|  | 6-paradol | Rhizome | *Zingiber officinale (Chukku)* |  | 94378 |
|  | 6-shogaol | Rhizome | *Zingiber officinale (Chukku)* |  | 5281794 |
|  | 7-gingerol | Rhizome | *Zingiber officinale (Chukku)* |  | 11472344 |
|  | 7-hydroxy-3',4'- (methylenedioxy)-flavan | Pericarp | *Terminalia bellirica (Thanthrikai)* |  | [139592630](https://pubchem.ncbi.nlm.nih.gov/compound/139592630) |
|  | 8-gingediol | Rhizome | *Zingiber officinale (Chukku)* |  | 101941698 |
|  | 8-gingerol | Rhizome | *Zingiber officinale (Chukku)* |  | 168114 |
|  | 8-Heptadecene | Flower, Root | *Piper longum (Thippili)* |  | 5364555 |
|  | 8-shogaol | Rhizome | *Zingiber officinale (Chukku)* |  | 6442560 |
|  | 9,17-Octadecadienal (Z) | Flower, Root | *Piper longum (Thippili)* |  | 5365667 |
|  | 9b-(3-Methylphenyl)-2,3-dihydrothiazolo[2,3-a]isoindol-5(9bH)-one | leaves, fruit | *carica papaya (Papaya)* |  | 2413 |
|  | 9-gingerol | Rhizome | *Zingiber officinale (Chukku)* |  | 102510965 |
|  | abieta-7 | Leaves | *Vitex negundo (Nochi)* |  | 443470 |
|  | acetaldehyde | Rhizome | *Zingiber officinale (Chukku)* |  | 177 |
|  | aceteugenol | Rhizome | *Acorus calamus ( Vasambu)* |  | [7136](https://pubchem.ncbi.nlm.nih.gov/compound/7136) |
|  | Acetic Acid | Rhizome | *Zingiber officinale (Chukku)* |  | 176 |
|  | acetone | Rhizome | *Zingiber officinale (Chukku)* |  | 180 |
|  | Acetoside | plant | *Clerodendrum serratum (Kanduparangi)* |  | 5281800 |
|  | acetosyringone | Leaf | *Cocos nucifera (Coconut Leaf Extract)* |  | [17198](https://pubchem.ncbi.nlm.nih.gov/compound/17198) |
|  | acetovanillone | Leaf | *Cocos nucifera (Coconut Leaf Extract)* |  | [2214](https://pubchem.ncbi.nlm.nih.gov/compound/2214) |
|  | acetyl-choline | seed | *Pepper nigrum (Pepper)* |  | *187* |
|  | acolamone | Rhizome | *Acorus calamus ( Vasambu)* |  | [71587142](https://pubchem.ncbi.nlm.nih.gov/compound/71587142) |
|  | acoradin | Rhizome | *Acorus calamus ( Vasambu)* |  | [126324](https://pubchem.ncbi.nlm.nih.gov/compound/126324) |
|  | acoramone | Rhizome | *Acorus calamus ( Vasambu)* |  | [3083746](https://pubchem.ncbi.nlm.nih.gov/compound/3083746) |
|  | [acorenone](https://phytochem.nal.usda.gov/phytochem/chemicals/show/3110) | Rhizome | *Acorus calamus ( Vasambu)* |  | [12480741](https://pubchem.ncbi.nlm.nih.gov/compound/12480741) |
|  | adhatodine | plant | *Justicia adhatoda (Aada thoda)* |  | 52908915 |
|  | aegelin | Leaf | *Aegle marmelos (Vilvam)* |  | *1558419* |
|  | aesculetin | Leaf | *Ocimum basilicum (Thiruneetrupachai)* |  | 5281416 |
|  | aesculin | Leaf | *Ocimum basilicum (Thiruneetrupachai)* |  | 5281417 |
|  | agnuside | Leaves | *Vitex negundo (Nochi)* |  | 442416 |
|  | alanine | Leaf | *Cocos nucifera (Coconut Leaf Extract)* |  | [5950](https://pubchem.ncbi.nlm.nih.gov/compound/5950) |
|  | allyl-catechol | Leaf | *Piper betle ( Vetrilai)* |  | *70775* |
|  | alpha amyrin | leaves | *Azadirachta indica (Neem)* |  | 73170 |
|  | alpha-amorphene | Flower | *Syzygium aromaticum (Lavangam)* |  | 12306052 |
|  | alpha-asarone | Rhizome | *Acorus calamus ( Vasambu)* |  | [636822](https://pubchem.ncbi.nlm.nih.gov/compound/636822) |
|  | alpha-atlantone | Root | *Curcuma longa (Turmeric)* |  | *12299867* |
|  | alpha-cadinene | Rhizome | *Zingiber officinale (Chukku)* |  | 12306048 |
|  | alpha-cadinol | Rhizome | *Zingiber officinale (Chukku)* |  | 10398656 |
|  | alpha-cedrene | Flower, Root | *Piper longum (Thippili)* |  | 6431015 |
|  | Alpha-Cubebene | Flower, Root | *Piper longum (Thippili)* |  | 42608159 |
|  | alpha-cubebene | Flower | *Syzygium aromaticum (Lavangam)* |  | 442359 |
|  | alpha-curcumene | Rhizome | *Zingiber officinale (Chukku)* |  | 92139 |
|  | alpha-Cyperone | Root | *Cyprus rotundus (Korai kilangu)* |  | 6452086 |
|  | alpha-farnesene | Rhizome | *Zingiber officinale (Chukku)* |  | 5281516 |
|  | alpha-humulene | Rhizome | *Acorus calamus ( Vasambu)* |  | [5281520](https://pubchem.ncbi.nlm.nih.gov/compound/5281520) |
|  | alpha-linolenic-acid | Rhizome | *Zingiber officinale (Chukku)* |  | 12306047 |
|  | alpha-phellandrene | Rhizome | *Zingiber officinale (Chukku)* |  | 7460 |
|  | alpha-pinene | Rhizome | *Acorus calamus ( Vasambu)* |  | [6654](https://pubchem.ncbi.nlm.nih.gov/compound/6654) |
|  | alpha-Rotunol | Root | *Cyprus rotundus (Korai kilangu)* |  | 11651583 |
|  | alpha-selinene | Rhizome | *Zingiber officinale (Chukku)* |  | 10856614 |
|  | alpha-terpinene | Rhizome | *Zingiber officinale (Chukku)* |  | 7462 |
|  | alpha-terpineol | Rhizome | *Acorus calamus ( Vasambu)* |  | [17100](https://pubchem.ncbi.nlm.nih.gov/compound/17100) |
|  | alpha-tocopherol | Root | *Curcuma longa (Turmeric)* |  | *14985* |
|  | alpha-zingiberene | Rhizome | *Zingiber officinale (Chukku)* |  | 11127403 |
|  | Amyrinacetate | leaves | *Azadirachta indica (Neem)* |  | 92156 |
|  | Andrographolide | plant | *Andrographis paniculata (Nilavembu)* |  | 5318517 |
|  | angelicoidenol | Rhizome | *Zingiber officinale (Chukku)* |  | 101254152 |
|  | anisotine | plant | *Justicia adhatoda (Aada thoda)* |  | 442884 |
|  | anthocyanin | Leaf | *Ocimum basilicum (Thiruneetrupachai)* |  | 145858 |
|  | aphanamol I | Flower, Root | *Piper longum (Thippili)* |  | 11031884 |
|  | Apigenin | Flowers | *Abutilon indicum (Thuthi)* |  | 5280443 |
|  |  | plant | *Clerodendrum serratum (Kanduparangi)* |  | 5280443 |
|  | arecaidine | Leaf | *Piper betle ( Vetrilai)* |  | [10355](https://pubchem.ncbi.nlm.nih.gov/compound/10355) |
|  | aristolene | Rhizome | *Acorus calamus ( Vasambu)* |  | [530421](https://pubchem.ncbi.nlm.nih.gov/compound/530421) |
|  | arjungenin | Fruit and pericarp | *Terminalia chebula (Kadukai)* |  | 12444386 |
|  | arjunolic-acid | Fruit and pericarp | *Terminalia chebula (Kadukai)* |  | 73641 |
|  | artemetin | Leaves | *Vitex negundo (Nochi)* |  | 5320351 |
|  | ar-turmerone | Root | *Curcuma longa (Turmeric)* |  | *160512* |
|  | asaronaldehyde | Rhizome | *Acorus calamus ( Vasambu)* |  | [20525](https://pubchem.ncbi.nlm.nih.gov/compound/20525) |
|  | Ascorbic acid | Fruits | *Pedalium murex ( Peru nerinji)* |  | 54670067 |
|  |  | Leaf | *Ocimum basilicum (Thiruneetrupachai)* |  | 54670067 |
|  |  | Root | *Cyprus rotundus (Korai kilangu)* |  | 54670067 |
|  |  | Fruit and pericarp | *Terminalia chebula (Kadukai)* |  | 54670067 |
|  |  | Leaf | *Plectranthus amboinicus (Karppuravalli)* |  | 54670067 |
|  |  | Leaf | *Solanum nigrum ( Manathakkali)* |  | 54670067 |
|  |  | Fruit | *Solanum torvum (sundakai)* |  | 54670067 |
|  | aucubin | Leaves | *Vitex negundo (Nochi)* |  | 91458 |
|  | Azadirachtanin | Leaf | *Azadirachta indica (Neem)* |  | [102146586](https://pubchem.ncbi.nlm.nih.gov/compound/102146586) |
|  | azulene | Rhizome | *Acorus calamus ( Vasambu)* |  | [9231](https://pubchem.ncbi.nlm.nih.gov/compound/9231) |
|  | benzaldehyde | Rhizome | *Zingiber officinale (Chukku)* |  | 240 |
|  | benzyl-acetate | Leaf | *Ocimum basilicum (Thiruneetrupachai)* |  | 8785 |
|  | Beta- asarone | Rhizome | *Acorus calamus ( Vasambu)* |  | 5281758 |
|  | beta-bisabolene | Rhizome | *Zingiber officinale (Chukku)* |  | 10104370 |
|  | beta-bisabolol | Rhizome | *Zingiber officinale (Chukku)* |  | 27208 |
|  | beta-carotene | Leaf | *Ocimum basilicum (Thiruneetrupachai)* |  | 5280489 |
|  |  | Leaf | *Plectranthus amboinicus (Karppuravalli)* |  | 5280489 |
|  |  | Leaf | *Solanum nigrum ( Manathakkali)* |  | 5280489 |
|  |  | Fruit | *Solanum torvum (sundakai)* |  | 5280489 |
|  | beta-caryophyllene (2) | seed | *Pepper nigrum (Pepper)* |  | *5281515* |
|  | beta-Cubebene | Flower, Root | *Piper longum (Thippili)* |  | 93081 |
|  | beta-cyperone | Rhizome | *Cyprus rotundus (Korai kilangu)* |  | 10889388 |
|  | beta-elemene | Rhizome | *Acorus calamus ( Vasambu)* |  | [6918391](https://pubchem.ncbi.nlm.nih.gov/compound/6918391) |
|  | Beta-glucogallin | Fruit | *Terminalia bellirica (Thanthrikai)* |  | 124021 |
|  |  | Fruit and pericarp | *Terminalia chebula (Kadukai)* |  | 124021 |
|  | beta-gurjunene | Rhizome | *Acorus calamus ( Vasambu)* |  | [6450812](https://pubchem.ncbi.nlm.nih.gov/compound/6450812) |
|  | beta-himachalene | Rhizome | *Zingiber officinale (Chukku)* |  | 11586487 |
|  | betaine | Flower | *Justicia adhatoda (Aada thoda)* |  | 247 |
|  | beta-ionone | Rhizome | *Zingiber officinale (Chukku)* |  | 638014 |
|  | beta-phellandrene | Rhizome | *Zingiber officinale (Chukku)* |  | 11142 |
|  | beta-pinene | Rhizome | *Acorus calamus ( Vasambu)* |  | [14896](https://pubchem.ncbi.nlm.nih.gov/compound/14896) |
|  |  | Rhizome | *Zingiber officinale (Chukku)* |  | 14896 |
|  |  | Root | *Cyprus rotundus (Korai kilangu)* |  | 14896 |
|  | beta-Rotunol | Root | *Cyprus rotundus (Korai kilangu)* |  | *5321005* |
|  | beta-selinene | Rhizome | *Zingiber officinale (Chukku)* |  | 442393 |
|  |  | Root | *Cyprus rotundus (Korai kilangu)* |  | *442393* |
|  | beta-sesquiphellandrene | Rhizome | *Zingiber officinale (Chukku)* |  | 12315492 |
|  | beta-sesquiphellandrol | Rhizome | *Zingiber officinale (Chukku)* |  | 91748291 |
|  | beta-sitosterol-beta-d-glucoside | plant | *Justicia adhatoda (Aada thoda)* |  | 12309055 |
|  | beta-thujone | Rhizome | *Zingiber officinale (Chukku)* |  | 91456 |
|  | beta-zingiberene | Rhizome | *Zingiber officinale (Chukku)* |  | 12315491 |
|  | Betulin | Root | *Hygrophila auriculata ( Mulli ver)* |  | 72326 |
|  | betulinic acid | Leaves | *Vitex negundo (Nochi)* |  | 64971 |
|  | bisabolene | Rhizome | *Zingiber officinale (Chukku)* |  | 3033866 |
|  |  | Root | *Curcuma longa (Turmeric)* |  | 3033866 |
|  | Bisdemethoxycurcumin | Flower, Root | *Piper longum (Thippili)* |  | 5315472 |
|  |  | Root | *Curcuma longa (Turmeric)* |  | 5315472 |
|  | borneol | Rhizome | *Zingiber officinale (Chukku)* |  | 64685 |
|  |  | Root | *Curcuma longa (Turmeric)* |  | 64685 |
|  | bornyl acetate | Leaves | *Vitex negundo (Nochi)* |  | 6448 |
|  | Brachyamide B | Flower, Root | *Piper longum (Thippili)* |  | 14162526 |
|  | cadinene | Leaf | *Piper betle ( Vetrilai)* |  | [3032853](https://pubchem.ncbi.nlm.nih.gov/compound/3032853) |
|  | Caffeic acid | Leaf | *Pedalium murex ( Peru nerinji)* |  | 689043 |
|  |  | Leaf | *Ocimum basilicum (Thiruneetrupachai)* |  | 689043 |
|  | caffeic-acid-n-butyl-ester | Leaf | *Ocimum basilicum (Thiruneetrupachai)* |  | 9991705 |
|  | calacorene | Flower | *Syzygium aromaticum (Lavangam)* |  | 12302243 |
|  | calamene | Rhizome | *Acorus calamus ( Vasambu)* |  | [518975](https://pubchem.ncbi.nlm.nih.gov/compound/518975) |
|  | calamenen | Rhizome | *Zingiber officinale (Chukku)* |  | 10798883 |
|  | calamenene | Rhizome | *Acorus calamus ( Vasambu)* |  | [6429077](https://pubchem.ncbi.nlm.nih.gov/compound/6429077) |
|  | calamenone | Rhizome | *Acorus calamus ( Vasambu)* |  | [101418274](https://pubchem.ncbi.nlm.nih.gov/compound/101418274) |
|  | calameone | Rhizome | *Acorus calamus ( Vasambu)* |  | [181982](https://pubchem.ncbi.nlm.nih.gov/compound/181982) |
|  | Calarene | Flower, root | *Piper longum (Thippili)* |  | 15560279 |
|  | Calcium oxalate | Stem | *Cissus_quadrangularis_L (Perandai)* |  | *33005* |
|  | Campesterol | Leaf | *Ocimum basilicum (Thiruneetrupachai)* |  | 173183 |
|  |  | Leaf | *Solanum trilobatum (Thoothuvalai)* |  | 173183 |
|  |  | plant | *Clerodendrum serratum (Kanduparangi)* |  | 173183 |
|  |  | Root | *Curcuma longa (Turmeric)* |  | 173183 |
|  | camphene | Rhizome | *Acorus calamus ( Vasambu)* |  | [6616](https://pubchem.ncbi.nlm.nih.gov/compound/6616) |
|  |  | Root | *Cyprus rotundus (Korai kilangu)* |  | 6616 |
|  | camphene-hydrate | Rhizome | *Zingiber officinale (Chukku)* |  | 101680 |
|  | camphor | Rhizome | *Acorus calamus ( Vasambu)* |  | [2537](https://pubchem.ncbi.nlm.nih.gov/compound/2537) |
|  | Capric acid | Root | *Abutilon indicum (Thuthi)* |  | 454075 |
|  | capric-acid | Rhizome | *Zingiber officinale (Chukku)* |  | 454065 |
|  | Caprylic acid | Root | *Abutilon indicum (Thuthi)* |  | 53874415 |
|  | caprylic-acid | Rhizome | *Zingiber officinale (Chukku)* |  | 454067 |
|  | capsaicin | Rhizome | *Zingiber officinale (Chukku)* |  | 1548943 |
|  | CAROTENE | Stem | *Cissus_quadrangularis_L (Perandai)* |  | *6419725* |
|  | carotene | Fruit | *Solanum nigrum ( Manathakkali)* |  | 5315263 |
|  |  | leaves | *Vitex negundo (Nochi)* |  | 5315263 |
|  | carvacrol | Leaf | *Plectranthus amboinicus (Karppuravalli)* |  | *10364* |
|  |  | Leaf | *Piper betle ( Vetrilai)* |  | *10364* |
|  | caryophyllene epoxide | Leaves | *Vitex negundo (Nochi)* |  | 14350 |
|  | Caryophyllene oxide | Flower, Root | *Piper longum (Thippili)* |  | 1742210 |
|  |  | Leaf | *Piper betle ( Vetrilai)* |  | 1742210 |
|  | caryophyllenol | Leaves | *Vitex negundo (Nochi)* |  | 61125 |
|  | casticin | Leaves | *Vitex negundo (Nochi)* |  | 5315263 |
|  | cedrol | Leaves | *Vitex negundo (Nochi)* |  | 65575 |
|  | Cedryl acetate | Flower, Root | *Piper longum (Thippili)* |  | 9838172 |
|  | chavicol | Rhizome | *Zingiber officinale (Chukku)* |  | 68148 |
|  |  | Leaf | *Piper betle ( Vetrilai)* |  | 68148 |
|  | Chebulic acid | Fruit | *Terminalia bellirica (Thanthrikai)* |  | [71308174](https://pubchem.ncbi.nlm.nih.gov/compound/71308174) |
|  | [chebulic-acid](https://phytochem.nal.usda.gov/phytochem/chemicals/show/5619) | Fruit and pericarp | *Terminalia chebula (Kadukai)* |  | *12302892* |
|  | chebulinic acid | Fruit and pericarp | *Terminalia chebula (Kadukai)* |  | 72284 |
|  | chlorogenin | Fruit | *Solanum torvum (sundakai)* |  | *12303065* |
|  | Cholestanol | plant | *Clerodendrum serratum (Kanduparangi)* |  | 6665 |
|  | choline | Rhizome | *Acorus calamus ( Vasambu)* |  | [305](https://pubchem.ncbi.nlm.nih.gov/compound/305) |
|  |  | seed | *Pepper nigrum (Pepper)* |  | [305](https://pubchem.ncbi.nlm.nih.gov/compound/305) |
|  | chrysanthemin | Rhizome | *Zingiber officinale (Chukku)* |  | 441667 |
|  | chrysoeriol | Leaf | *Plectranthus amboinicus (Karppuravalli)* |  | *5280666* |
|  | cineole | Leaf | *Piper betle ( Vetrilai)* |  | *2758* |
|  |  | Leaf | *Aegle marmelos (Vilvam)* |  | 2758 |
|  | cinnamaldehyde | Flower | *Syzygium aromaticum (Lavangam)* |  | 637511 |
|  | cinnamic-acid-methyl-ester | Leaf | *Ocimum basilicum (Thiruneetrupachai)* |  | 5314585 |
|  | cirsilineol | Leaf | *Ocimum tenuiflorum (Tulsi)* |  | *162464* |
|  | cis-.beta.-Elemenediastereomer | Flower, Root | *Piper longum (Thippili)* |  | 6431152 |
|  | Cis-2-Decalone | Flower, Root | *Piper longum (Thippili)* |  | 246289 |
|  | Cis-Decahydronaphthalene | Flower, Root | *Piper longum (Thippili)* |  | 7044 |
|  | cis-geranic-acid | Rhizome | *Zingiber officinale (Chukku)* |  | 5312583 |
|  | cis-isoasarone | Rhizome | *Acorus calamus ( Vasambu)* |  | [5281758](https://pubchem.ncbi.nlm.nih.gov/compound/5281758) |
|  | cis-rose-oxide | Rhizome | *Zingiber officinale (Chukku)* |  | 27866 |
|  | cis-sesquisabinene-hydrate | Rhizome | *Zingiber officinale (Chukku)* |  | 91666343 |
|  | citral | Leaf | *Aegle marmelos (Vilvam)* |  | 638011 |
|  | citronellal | Rhizome | *Zingiber officinale (Chukku)* |  | 7794 |
|  | citronellol | Rhizome | *Zingiber officinale (Chukku)* |  | 8842 |
|  | citronellol-acetate | Rhizome | *Zingiber officinale (Chukku)* |  | 6708682 |
|  | citronellyl-acetate | Rhizome | *Zingiber officinale (Chukku)* |  | 9017 |
|  | Clerodermic acid | plant | *Clerodendrum serratum (Kanduparangi)* |  | 16745295 |
|  | Clerosterol | plant | *Clerodendrum serratum (Kanduparangi)* |  | 5283638 |
|  | Copaene | Flower, Root | *Piper longum (Thippili)* |  | 25245021 |
|  | [copaene](https://phytochem.nal.usda.gov/phytochem/chemicals/show/6220) | Essential oil | *Cyprus rotundus (Korai kilangu)* |  | 12303902 |
|  | coriandrin | Leaf | *Coriandrum sativum (Coriander)* |  | 119586 |
|  | corilagin | Fruit | *Terminalia bellirica (Thanthrikai)* |  | 73568 |
|  |  | Fruit and pericarp | *Terminalia chebula (Kadukai)* |  | 73568 |
|  | corymbosin | Leaves | *Vitex negundo (Nochi)* |  | 10970376 |
|  | Coumaperine | Flower, Root | *Piper longum (Thippili)* |  | 10131321 |
|  | crataegolic-acid | Flower | *Syzygium aromaticum (Lavangam)* |  | 73659 |
|  | cumene | Rhizome | *Zingiber officinale (Chukku)* |  | 7406 |
|  | cuminaldehyde | Leaf | *Aegle marmelos (Vilvam)* |  | 326 |
|  | curcumin | Rhizome | *Zingiber officinale (Chukku)* |  | 969516 |
|  |  | Root | *Curcuma longa (Turmeric)* |  | 969516 |
|  | cyanin | Rhizome | *Zingiber officinale (Chukku)* |  | 441688 |
|  | Cyclodecene, 1-methyl- | Flower, Root | *Piper longum (Thippili)* |  | 5367581 |
|  | Cyclopentadecane | Flower, Root | *Piper longum (Thippili)* |  | 67525 |
|  | Cyperene | Essential oil | *Cyprus rotundus (Korai kilangu)* |  | 99856 |
|  | Cyperol | Essential oil | *Cyprus rotundus (Korai kilangu)* |  | 14076601 |
|  | cyperotundone | Essential oil | *Cyprus rotundus (Korai kilangu)* |  | 12308615 |
|  | daucosterol | Fruit and pericarp | *Terminalia chebula (Kadukai)* |  | 5742590 |
|  | d-borneol | Rhizome | *Zingiber officinale (Chukku)* |  | 6552009 |
|  | D-Camphor | Flower, Root | *Piper longum (Thippili)* |  | 159055 |
|  | decan-1-al | Rhizome | *Zingiber officinale (Chukku)* |  | 19898624 |
|  | decyl-aldehyde | Rhizome | *Zingiber officinale (Chukku)* |  | 8175 |
|  |  | Leaf | *Ocimum tenuiflorum (Tulsi)* |  | 8175 |
|  | Dehydropipernonaline | Flower, Root | *Piper longum (Thippili)* |  | 6439947 |
|  | delta-cadinene | Rhizome | *Acorus calamus (Vasambu)* |  | [441005](https://pubchem.ncbi.nlm.nih.gov/compound/441005) |
|  | delta-carene | Rhizome | *Acorus calamus (Vasambu)* |  | [26049](https://pubchem.ncbi.nlm.nih.gov/compound/26049) |
|  | Delta-elemene | Flower, Root | *Piper longum (Thippili)* |  | 12309449 |
|  | Demethoxycurcumin | Flower, Root | *Piper longum (Thippili)* |  | 5469424 |
|  |  | Root | *Curcuma longa (Turmeric)* |  | 5469424 |
|  | dextrin | Rhizome | *Acorus calamus ( Vasambu)* |  | [62698](https://pubchem.ncbi.nlm.nih.gov/compound/62698) |
|  | diethylsulfide | Rhizome | *Zingiber officinale (Chukku)* |  | 9609 |
|  | Dihydropiperlonguminine | Flower, Root | *Piper longum (Thippili)* |  | 12682184 |
|  | dimethoxyflavonone | Leaves | *Vitex negundo (Nochi)* |  | 378567 |
|  | Dinatin | Leaves, Stem & Flowers | *Pedalium murex ( Peru nerinji)* |  | 5281628 |
|  | Diosgenin | Fruits | *Pedalium murex ( Peru nerinji)* |  | 99474 |
|  |  | Fruit | *Solanum nigrum ( Manathakkali)* |  | 99474 |
|  |  | Plant | *Solanum nigrum ( Manathakkali)* |  | 99474 |
|  | Diosmetin | Leaves & Fruits | *Pedalium murex ( Peru nerinji)* |  | 5281612 |
|  | d-limonene | Leaf | *phyllanthus niruri ( Keelanelli)* |  | *440917* |
|  |  | Leaf | *Aegle marmelos (Vilvam)* |  | 440917 |
|  | Dodecanal | Flower, Root | *Piper longum (Thippili)* |  | 8194 |
|  | dodecanoic-acid | Rhizome | *Zingiber officinale (Chukku)* |  | 2762668 |
|  | dodecyl-p-coumarate | Plant extract | *Ipomea carnea (Neiveli Kaatamanakku)* |  | 42642759 |
|  | e-05a | Leaf | *Piper betle ( Vetrilai)* |  | *6438619* |
|  |  | Leaf | *Aegle marmelos (Vilvam)* |  | 6438619 |
|  | elemicin | Rhizome | *Acorus calamus ( Vasambu)* |  | [10248](https://pubchem.ncbi.nlm.nih.gov/compound/10248) |
|  | elemol | Rhizome | *Zingiber officinale (Chukku)* |  | 92138 |
|  | Ellagic acid | Fruit | *Terminalia bellirica (Thanthrikai)* |  | 5281855 |
|  |  | Fruit and pericarp | *Terminalia chebula (Kadukai)* |  | 5281855 |
|  | eo | Rhizome | *Acorus calamus (Vasambu)* |  | [6438619](https://pubchem.ncbi.nlm.nih.gov/compound/6438619) |
|  | Epieudesmin | Flower, Root | *Piper longum (Thippili)* |  | 7299790 |
|  | epishyobunone | Rhizome | *Acorus calamus (Vasambu)* |  | [591309](https://pubchem.ncbi.nlm.nih.gov/compound/591309) |
|  | epoxyisoacoragermacrone | Rhizome | *Acorus calamus (Vasambu)* |  | [101652306](https://pubchem.ncbi.nlm.nih.gov/compound/101652306) |
|  | eriodictyol | Leaf | *Ocimum basilicum (Thiruneetrupachai)* |  | 440735 |
|  | eriodictyol-7-o-beta-d-glucoside | Leaf | *Ocimum basilicum (Thiruneetrupachai)* |  | 5319853 |
|  | eriodictyol-7-o-glucoside | Leaf | *Ocimum basilicum (Thiruneetrupachai)* |  | 13254473 |
|  | escopoletin | Plant extract | *Ipomea carnea (Neiveli Kaatamanakku)* |  | 5280460 |
|  | esculetin | Leaf | *Ocimum basilicum (Thiruneetrupachai)* |  | 5281416 |
|  | esculin | Leaf | *Ocimum basilicum (Thiruneetrupachai)* |  | 5281417 |
|  | estragole | Leaf | *Ocimum basilicum (Thiruneetrupachai)* |  | 8815 |
|  |  | Leaf | *Piper betle ( Vetrilai)* |  | 8815 |
|  | ethanol | Rhizome | *Acorus calamus (Vasambu)* |  | [702](https://pubchem.ncbi.nlm.nih.gov/compound/702) |
|  | ethyl-acetate | Rhizome | *Zingiber officinale (Chukku)* |  | 8857 |
|  | ethyl-isopropyl-sulfide | Rhizome | *Zingiber officinale (Chukku)* |  | 21228 |
|  | ethyl-myristate | Rhizome | *Zingiber officinale (Chukku)* |  | 31283 |
|  | Etritinate | leaves, fruit | *carica papaya (Papaya)* |  | 5282375 |
|  | eugenol | Rhizome | *Acorus calamus (Vasambu)* |  | [3314](https://pubchem.ncbi.nlm.nih.gov/compound/3314) |
|  |  | Leaf | *Ocimum basilicum (Thiruneetrupachai)* |  | 3314 |
|  |  | Leaf | *Ocimum tenuiflorum (Tulsi)* |  | 3314 |
|  |  | Leaf | *Piper betle ( Vetrilai)* |  | 3314 |
|  | Eugenol methyl ether | Rhizome | *Acorus calamus (Vasambu)* |  | 7127 |
|  | Fargesin | Flower, Root | *Piper longum (Thippili)* |  | 10926754 |
|  | farnesal | Rhizome | *Zingiber officinale (Chukku)* |  | 5280598 |
|  | Ferulic acid | Leaf | *Cocos nucifera (Coconut Leaf Extract)* |  | [445858](https://pubchem.ncbi.nlm.nih.gov/compound/445858) |
|  |  | Leaves | *Pedalium murex ( Peru nerinji)* |  | 445858 |
|  | Flavylium perchlorate | Fruit | *Solanum nigrum ( Manathakkali)* |  | *145857* |
|  | Friedelin | Leaves | *Vitex negundo (Nochi)* |  | 91472 |
|  |  | Leaves | *Acorus calamus ( Vasambu)* |  | 91472 |
|  | furanogermenone | Rhizome | *Zingiber officinale (Chukku)* |  | 6439596 |
|  | furfural | Rhizome | *Acorus calamus (Vasambu)* |  | [7362](https://pubchem.ncbi.nlm.nih.gov/compound/7362) |
|  | furfuryl-alcohol | Flower | *Syzygium aromaticum (Lavangam)* |  | 7361 |
|  | galangin | Rhizome | *Acorus calamus ( Vasambu)* |  | [5281616](https://pubchem.ncbi.nlm.nih.gov/compound/5281616) |
|  | galanolactone | Rhizome | *Zingiber officinale (Chukku)* |  | 11141699 |
|  | gallic acid | Fruit and pericarp | *Terminalia chebula (Kadukai)* |  | 370 |
|  |  | Flower | *Syzygium aromaticum (Lavangam)* |  | 370 |
|  | gallic-acid-ethyl-ester | Fruit and pericarp | *Terminalia chebula (Kadukai)* |  | 13250 |
|  | gamma-aminobutyric-acid | Rhizome | *Zingiber officinale (Chukku)* |  | 119 |
|  | gamma-atlantone | Root | *Curcuma longa (Turmeric)* |  | *91698329* |
|  | gamma-eudesmol | Rhizome | *Zingiber officinale (Chukku)* |  | 6432005 |
|  | gamma-muurolene | Rhizome | *Zingiber officinale (Chukku)* |  | 12313020 |
|  | gamma-selinene | Rhizome | *Zingiber officinale (Chukku)* |  | 521334 |
|  |  | Leaf | *Ocimum tenuiflorum (Tulsi)* |  | 521334 |
|  | gamma-sitosterol | Leaf | *Aegle marmelos (Vilvam)* |  | 521334 |
|  | gentiobiose | Fruit and pericarp | *Terminalia chebula (Kadukai)* |  | 441422 |
|  | gentisic-acid | Leaf | *Cocos nucifera ( coconut Leaf Etract)* |  | [24101](https://pubchem.ncbi.nlm.nih.gov/compound/24101) |
|  | geranial | Leaves | *Vitex negundo (Nochi)* |  | 638011 |
|  | geraniol | Rhizome | *Zingiber officinale (Chukku)* |  | 637566 |
|  | geranyl-acetate | Leaf | *Ocimum basilicum (Thiruneetrupachai)* |  | 1549026 |
|  | gingediacetate | Rhizome | *Zingiber officinale (Chukku)* |  | 5317587 |
|  | gingerenone-a | Rhizome | *Zingiber officinale (Chukku)* |  | 5281775 |
|  | gingerenone-b | Rhizome | *Zingiber officinale (Chukku)* |  | 5317592 |
|  | gingerenone-c | Rhizome | *Zingiber officinale (Chukku)* |  | 5317593 |
|  | gingerone | Rhizome | *Zingiber officinale (Chukku)* |  | 31211 |
|  | Glabridin | root | *Glycyrrhiza glabra (Liquo rice)* |  | 124052 |
|  | globulol | Leaves | *Vitex negundo (Nochi)* |  | 12304985 |
|  | Glucobrassicin | Flower | *Brassica oleracea ( Cabbage)* |  | 656506 |
|  | Glucopyranoside | Flowers | *Abutilon indicum (Thuthi)* |  | 5793 |
|  | glutamic-acid | Leaf | *Cocosnucifera (Coocnut Leaf Extract)* |  | [33032](https://pubchem.ncbi.nlm.nih.gov/compound/33032) |
|  | glycine | Leaf | *Cocosnucifera (Coocnut Leaf Extract)* |  | 750 |
|  | guaiene | Rhizome | *Acorus calamus ( Vasambu)* |  | [6949](https://pubchem.ncbi.nlm.nih.gov/compound/6949) |
|  | Guineensine | Flower, Root | *Piper longum (Thippili)* |  | 6442405 |
|  | Hederagenin | seed | *Nigella sativa ( Fennel)* |  | 73299 |
|  | heptadecanoic-acid | Rhizome | *Zingiber officinale (Chukku)* |  | 10465 |
|  | Heptadecene | Flower, Root | *Piper longum (Thippili)* |  | 23217 |
|  | heptan-2-ol | Rhizome | *Zingiber officinale (Chukku)* |  | 10976 |
|  | heptan-2-one | Rhizome | *Zingiber officinale (Chukku)* |  | 8051 |
|  | hexahydrocurcumin | Rhizome | *Zingiber officinale (Chukku)* |  | 5318039 |
|  | Hexahydropyridine | Flower, Root | *Piper longum (Thippili)* |  | 8082 |
|  | hexan-1-al | Rhizome | *Zingiber officinale (Chukku)* |  | 6184 |
|  | hexan-1-ol | Rhizome | *Zingiber officinale (Chukku)* |  | 8103 |
|  | hispidulin | plant | *Clerodendrum serratum (Kanduparangi)* |  | 5281628 |
|  | histidine | Leaf | *Cocos nucifera (Coconut Leaf Extract)* |  | [6274](https://pubchem.ncbi.nlm.nih.gov/compound/6274) |
|  | humulene-epoxide | Flower | *Syzygium aromaticum (Lavangam)* |  | 5352470 |
|  | humulene-epoxide-2 | Rhizome | *Zingiber officinale (Chukku)* |  | 10704181 |
|  | Hydrocinnamic acid | Flower, Root | *Piper longum (Thippili)* |  | 107 |
|  | hydroxy-chavicol | Leaf | *Piper betle ( Vetrilai)* |  | *70775* |
|  | Hyperoside | Leaf | *Azadirachta indica (Neem)* |  | [5281643](https://pubchem.ncbi.nlm.nih.gov/compound/5281643) |
|  | Hypnon | Flower, Root | *Piper longum (Thippili)* |  | 7410 |
|  | Isatin | Fruits | *Pedalium murex ( Peru nerinji)* |  | 7054 |
|  | isoacolamone | Rhizome | *Acorus calamus (Vasambu)* |  | [71587143](https://pubchem.ncbi.nlm.nih.gov/compound/71587143) |
|  | isoacorone | Rhizome | *Acorus calamus (Vasambu)* |  | [98934](https://pubchem.ncbi.nlm.nih.gov/compound/98934) |
|  | Isoborneol | Flower, Root | *Piper longum (Thippili)* |  | 6321405 |
|  | isobutylamide | Root | *Anacyclus pyrethrum (Akara)* |  | 68424 |
|  | Isobutylisovalerate | Flower, Root | *Piper longum (Thippili)* |  | 11514 |
|  | Isocaryophyllene | Flower, Root | *Piper longum (Thippili)* |  | 5281522 |
|  | Isochlorogenic acid | Plant | *Solanum nigrum ( Manathakkali)* |  | *6436237* |
|  | Isodiprene | Flower, Root | *Piper longum (Thippili)* |  | 443156 |
|  | isoeugenol | Rhizome | *Acorus calamus (Vasambu)* |  | [853433](https://pubchem.ncbi.nlm.nih.gov/compound/853433) |
|  | isoeugenol-methyl-ether | Rhizome | *Zingiber officinale (Chukku)* |  | 637776 |
|  | isogingerenone-b | Rhizome | *Zingiber officinale (Chukku)* |  | 5318568 |
|  | isoleucine | Leaf | *Ocimum basilicum (Thiruneetrupachai)* |  | 6306 |
|  | Isolongifolene epoxide | Flower, Root | *Piper longum (Thippili)* |  | 107035 |
|  | isoquercitrin | Leaf | *Ocimum basilicum (Thiruneetrupachai)* |  | 5280804 |
|  |  | Fruit and pericarp | *Terminalia chebula (Kadukai)* |  | 5280804 |
|  | Isorientin | Leaves | *Vitex negundo (Nochi)* |  | 114776 |
|  | isoshyobunone | Rhizome | *Acorus calamus ( Vasambu)* |  | [5318673](https://pubchem.ncbi.nlm.nih.gov/compound/5318673) |
|  | isothymonin | Leaf | *Ocimum tenuiflorum (Tulsi)* |  | *11726019* |
|  | isothymusin | Leaf | *Ocimum tenuiflorum (Tulsi)* |  | *630253* |
|  | isovaleraldehyde | Rhizome | *Zingiber officinale (Chukku)* |  | 9569524 |
|  | kaempferol | Leaf | *Ocimum basilicum (Thiruneetrupachai)* |  | 5280863 |
|  | Lauric acid | Root | *Abutilon indicum (Thuthi)* |  | 3893 |
|  | Lawsone | Flower, Root | *Piper longum (Thippili)* |  | 6755 |
|  | leucine | Leaf | *Cocos nucifera (Coconut Leaf Extract)* |  | [6106](https://pubchem.ncbi.nlm.nih.gov/compound/6106) |
|  | limonene | Rhizome | *Acorus calamus ( Vasambu)* |  | [22311](https://pubchem.ncbi.nlm.nih.gov/compound/22311) |
|  |  | Seed essential oil | *Pepper nigrum (Pepper)* |  | [22311](https://pubchem.ncbi.nlm.nih.gov/compound/22311) |
|  | linalol | Rhizome | *Acorus calamus ( Vasambu)* |  | [6549](https://pubchem.ncbi.nlm.nih.gov/compound/6549) |
|  |  | Leaf | *Ocimum tenuiflorum (Tulsi)* |  | [6549](https://pubchem.ncbi.nlm.nih.gov/compound/6549) |
|  |  | Leaf | *Ocimum basilicum (Thiruneetrupachai)* |  | 6549 |
|  | linalool-oxide | Rhizome | *Zingiber officinale (Chukku)* |  | 6432254 |
|  | Linoleic acid | Root | *Abutilon indicum (Thuthi)* |  | 5280450 |
|  |  | Seed oil | *Terminalia chebula (Kadukai)* |  | 5280450 |
|  |  | seed | *Solanum nigrum ( Manathakkali)* |  | 5280450 |
|  | Liquiritigenin | root | *Glycyrrhiza glabra ( Liquo rice)* |  | 114829 |
|  | Lupeol | leaves | *Azadirachta indica (Neem)* |  | 259846 |
|  |  | Leaves, fruit | *Carica papaya (Papaya)* |  | 259846 |
|  | Lupeol acetate | Fruits | *Pedalium murex ( Peru nerinji)* |  | 92157 |
|  | Lutein | leaves | *Azadirachta indica (Neem)* |  | 5281243 |
|  | Luteolin | Flowers | *Abutilon indicum (Thuthi)* |  | 5280445 |
|  |  | Leaf | *Ocimum tenuiflorum (Tulsi)* |  | 5280445 |
|  | Luteolin 7-O-(6''-malonylglucoside) | Leaves | *Vitex negundo (Nochi)* |  | 5281669 |
|  | Luteolin- 7- o- beta-D-Glucoside | Leaves | *Vitex negundo (Nochi)* |  | 5280637 |
|  | luteolin-7-o-beta-d-glucopyranoside | Leaf | *Ocimum tenuiflorum (Tulsi)* |  | *5291488* |
|  | lysine | Leaf | *Cocos nucifera (Coconut Leaf Extract)* |  | [5962](https://pubchem.ncbi.nlm.nih.gov/compound/5962) |
|  | l-Zingiberene | Flower, Root | *Piper longum (Thippili)* |  | 521253 |
|  | Mannitol | plant | *Clerodendrum serratum (Kanduparangi)* |  | 6251 |
|  | MEDIORESINOL | Plant | *Solanum nigrum ( Manathakkali)* |  | *181681* |
|  | melatonin | Rhizome | *Zingiber officinale (Chukku)* |  | 896 |
|  | menthol | Rhizome | *Acorus calamus (Vasambu)* |  | [1254](https://pubchem.ncbi.nlm.nih.gov/compound/1254) |
|  | menthol-acetate | Rhizome | *Zingiber officinale (Chukku)* |  | 6432249 |
|  | menthone | Rhizome | *Acorus calamus (Vasambu)* |  | [26447](https://pubchem.ncbi.nlm.nih.gov/compound/26447) |
|  | methionine | Leaf | *Cocos nucifera (Coconut Leaf Extract)* |  | [876](https://pubchem.ncbi.nlm.nih.gov/compound/876) |
|  | Methyl hydrocinnamate | Flower, Root | *Piper longum (Thippili)* |  | 7643 |
|  | methyl-acetate | Rhizome | *Zingiber officinale (Chukku)* |  | 6584 |
|  | methyl-allyl-sulfide | Rhizome | *Zingiber officinale (Chukku)* |  | 14640451 |
|  | methyl-amine | Rhizome | *Acorus calamus ( Vasambu)* |  | [6329](https://pubchem.ncbi.nlm.nih.gov/compound/6329) |
|  | methyl-chavicol | Leaf | *Ocimum tenuiflorum (Tulsi)* |  | *8815* |
|  | methyl-eugenol | Rhizome | *Acorus calamus ( Vasambu)* |  | [45266909](https://pubchem.ncbi.nlm.nih.gov/compound/45266909) |
|  |  | Leaf | *Ocimum tenuiflorum (Tulsi)* |  | [45266909](https://pubchem.ncbi.nlm.nih.gov/compound/45266909) |
|  | methyl-isoeugenol | Rhizome | *Acorus calamus ( Vasambu)* |  | [7128](https://pubchem.ncbi.nlm.nih.gov/compound/7128) |
|  | methyl-nonyl-ketone | Rhizome | *Zingiber officinale (Chukku)* |  | 8163 |
|  | methyl-salicylate | Flower | *Syzygium aromaticum (Lavangam)* |  | 4133 |
|  | molludistin | Leaf | *Ocimum tenuiflorum (Tulsi)* |  | *44258315* |
|  | monodemethoxycurcumin | Root | *Curcuma longa (Turmeric)* |  | *5469424* |
|  | Mustakone | Root | *Cyprus rotundus (Korai kilangu)* |  | 24816380 |
|  | myrcene | Leaf | *Ocimum basilicum (Thiruneetrupachai)* |  | 31253 |
|  |  | Leaf | *Ocimum tenuiflorum (Tulsi)* |  | 31253 |
|  | Myristic acid | Root | *Abutilon indicum (Thuthi)* |  | 11005 |
|  | N-(2,5-dimethoxyphenyl)-4-methoxybenzamide | Flower, Root | *Piper longum (Thippili)* |  | 532276 |
|  | N-[(2E,4E)-Decadienoyl]-piperidine | Flower, Root | *Piper longum (Thippili)* |  | 11118018 |
|  | N-[(2E,4E)-Tetradecadienoyl]piperidine | Flower, Root | *Piper longum (Thippili)* |  | 11130083 |
|  | N-[(4R)-4-(3-Fluorophenyl)-6-oxo-4,5-dihydro-1H-pyrimidin-2-yl]-3-methoxybenzamide | leaves, fruit | *carica papaya (Papaya)* |  | 92655510 |
|  | n-butyraldehyde | Rhizome | *Zingiber officinale (Chukku)* |  | 261 |
|  | Naphthalene | Flower | *Syzygium aromaticum (Lavangam)* |  | 931 |
|  | Neoandrographolide | plant | *Andrographis paniculata (Nilavembu)* |  | 9848024 |
|  | neral | Leaves | *Vitex negundo (Nochi)* |  | 643779 |
|  | Nerol | Leaf | *Ocimum tenuiflorum (Tulsi)* |  | *643820* |
|  |  | Rhizome | *Zingiber officinale (Chukku)* |  | *643820* |
|  | nerolidol | Leaves | *Vitex negundo (Nochi)* |  | 5284507 |
|  | nerol-oxide | Rhizome | *Zingiber officinale (Chukku)* |  | 61275 |
|  | N-Heptadecane | Flower, Root | *Piper longum (Thippili)* |  | 12398 |
|  | n-heptane | Rhizome | *Zingiber officinale (Chukku)* |  | 8900 |
|  | n-heptylic-acid | Rhizome | *Acorus calamus ( Vasambu)* |  | [8094](https://pubchem.ncbi.nlm.nih.gov/compound/8094) |
|  | niacin | Leaf | *Ocimum basilicum (Thiruneetrupachai)* |  | 938 |
|  |  | Leaf | *Plectranthus amboinicus (Karppuravalli)* |  | 938 |
|  |  | Fruit | *Solanum torvum (sundakai)* |  | 938 |
|  | Nimbandiol | Leaf | *Azadirachta indica (Neem)* |  | [157277](https://pubchem.ncbi.nlm.nih.gov/compound/157277) |
|  | Nimbinene | Leaf | *Azadirachta indica (Neem)* |  | [44715635](https://pubchem.ncbi.nlm.nih.gov/compound/44715635) |
|  | Nimboflavone | Leaf | *Azadirachta indica (Neem)* |  | [14492795](https://pubchem.ncbi.nlm.nih.gov/compound/14492795) |
|  | niranthin | Leaf | *phyllanthus niruri ( Keelanelli)* |  | *13989915* |
|  | nirtetralin | Leaf | *phyllanthus niruri ( Keelanelli)* |  | *182644* |
|  | N-isobutyl-2E,4E-dodecadienamide | Flower, Root | *Piper longum (Thippili)* |  | 6443006 |
|  | N-Isobutyl-2E,4E-hexadecadienamide | Flower, Root | *Piper longum (Thippili)* |  | 6442402 |
|  | N-Isobutyl-2E,4E-undecadienamide | Flower, Root | *Piper longum (Thippili)* |  | 20157325 |
|  | N-Methylsolasodine | Plant | *Solanum nigrum ( Manathakkali)* |  | *21573751* |
|  | n-nonane | Rhizome | *Zingiber officinale (Chukku)* |  | 8141 |
|  | n-nonanol | Rhizome | *Zingiber officinale (Chukku)* |  | 8914 |
|  | n-octane | Rhizome | *Zingiber officinale (Chukku)* |  | 356 |
|  | n-octanol | Rhizome | *Zingiber officinale (Chukku)* |  | 957 |
|  | nonan-1-al | Rhizome | *Zingiber officinale (Chukku)* |  | 132275663 |
|  | nonan-2-ol | Rhizome | *Zingiber officinale (Chukku)* |  | 12367 |
|  | nonan-2-one | Rhizome | *Zingiber officinale (Chukku)* |  | 13187 |
|  | nonanal | Rhizome | *Zingiber officinale (Chukku)* |  | 31289 |
|  | n-propanol | Rhizome | *Zingiber officinale (Chukku)* |  | 1031 |
|  | o-(3,3-dimethylallyl)-halfordinol | Leaf | *Aegle marmelos (Vilvam)* |  | 617263 |
|  | ocimarin | Leaf | *Ocimum tenuiflorum (Tulsi)* |  | *5288573* |
|  | octadeca-9,12-dienoic acid | Plant | *Solanum nigrum ( Manathakkali)* |  | *3931* |
|  | octan-1-al | Rhizome | *Zingiber officinale (Chukku)* |  | 21437548 |
|  | octan-2-ol | Rhizome | *Zingiber officinale (Chukku)* |  | 20083 |
|  | Oleanolic acid | Tuber | *Cyprus rotundus (Korai kilangu)* |  | 10494 |
|  |  | plant | *Clerodendrum serratum (Kanduparangi)* |  | 10494 |
|  |  | Leaf | *Ocimum basilicum (Thiruneetrupachai)* |  | 10494 |
|  |  | Leaf | *Ocimum tenuiflorum (Tulsi)* |  | 10494 |
|  | oleic acid | Seed oil | *Terminalia chebula (Kadukai)* |  | 445639 |
|  |  | leaves, fruit | *carica papaya (Papaya)* |  | 445639 |
|  |  | Rhizome | *Zingiber officinale (Chukku)* |  | 445639 |
|  |  | seed | *Solanum nigrum ( Manathakkali)* |  | 445639 |
|  | ornithine | Leaf | *Piper betle ( Vetrilai)* |  | *6262* |
|  | oscine | plant | *Justicia adhatoda (Aada thoda)* |  | 5184 |
|  | oxalic-acid | Leaf | *Plectranthus amboinicus (Karppuravalli)* |  | *971* |
|  | Palmitic acid | Root | *Abutilon indicum (Thuthi)* |  | 985 |
|  |  | Fruit and pericarp | *Terminalia chebula (Kadukai)* |  | 985 |
|  |  | Seed oil | *Terminalia chebula (Kadukai)* |  | 985 |
|  |  | seed | *Solanum nigrum ( Manathakkali)* |  | 985 |
|  |  | Rhizome | *Acorus calamus ( Vasambu)* |  | 985 |
|  | palmitoleic-acid | Rhizome | *Zingiber officinale (Chukku)* |  | 445638 |
|  | p-Amino-o-cresol | Flower, Root | *Piper longum (Thippili)* |  | 76081 |
|  | Paniculide B | plant | *Andrographis paniculata (Nilavembu)* |  | 101289823 |
|  | Paniculide C | plant | *Andrographis paniculata (Nilavembu)* |  | 101289824 |
|  | paniculide-a | plant | *Andrographis paniculata (Nilavembu)* |  | *11821485* |
|  | pantothenic-acid | Rhizome | *Zingiber officinale (Chukku)* |  | 6613 |
|  | Papaverinol | leaves, fruit | *carica papaya (Papaya)* |  | 275192 |
|  | patchouli-alcohol | Rhizome | *Zingiber officinale (Chukku)* |  | 10955174 |
|  | p-Coumaric acid | Leaves | *Pedalium murex ( Peru nerinji)* |  | 637542 |
|  |  | Leaf | *Cocos nucifera (Coconut Leaf Extract)* |  | [637542](https://pubchem.ncbi.nlm.nih.gov/compound/637542) |
|  |  | Leaf | *Ocimum basilicum (Thiruneetrupachai)* |  | 637542 |
|  | p-cymene | Leaf | *phyllanthus niruri ( Keelanelli)* |  | *7463* |
|  |  | Leaf | *Aegle marmelos (Vilvam)* |  | 7463 |
|  |  | Leaf | *Piper betle ( Vetrilai)* |  | *7463* |
|  |  | Rhizome | *Acorus calamus ( Vasambu)* |  | *7463* |
|  | P-cymol | Root | *Cyprus rotundus (Korai kilangu)* |  | 7463 |
|  | Pectin | Root | *Cyprus rotundus (Korai kilangu)* |  | 441476 |
|  |  | Fruit and pericarp | *Terminalia chebula (Kadukai)* |  | 441476 |
|  |  | Fruit,leaf | *Solanum torvum (sundakai)* |  | 441476 |
|  | Pedalitin | Leaves, Stem & Fruits | *Pedalium murex ( Peru nerinji)* |  | 31161 |
|  | peganine | plant | *Justicia adhatoda (Aada thoda)* |  | 72610 |
|  | Pellitorine | Flower, Root | *Piper longum (Thippili)* |  | 5318516 |
|  |  | Root | *Anacyclus pyrethrum (Akara)* |  | 5318516 |
|  | Pentadecane | Flower, Root | *Piper longum (Thippili)* |  | 12391 |
|  | pentadecanoic-acid | Rhizome | *Zingiber officinale (Chukku)* |  | 13849 |
|  | Pentadecene | Flower, Root | *Piper longum (Thippili)* |  | 25913 |
|  | pentan-2-ol | Rhizome | *Zingiber officinale (Chukku)* |  | 22386 |
|  | perillen | Rhizome | *Zingiber officinale (Chukku)* |  | 68316 |
|  | phellandrene | Seed essential oil | *Pepper nigrum (Pepper)* |  | *7460* |
|  |  | Leaf | *Aegle marmelos (Vilvam)* |  | 7460 |
|  | phenylalanine | Leaf | *Cocos nucifera (Coconut Leaf Extract)* |  | [6140](https://pubchem.ncbi.nlm.nih.gov/compound/6140) |
|  | phenylethyl-acetate | Flower | *Syzygium aromaticum (Lavangam)* |  | 62341 |
|  | phenylpropane | Rhizome | *Acorus calamus ( Vasambu)* |  | [7668](https://pubchem.ncbi.nlm.nih.gov/compound/7668) |
|  | p-hydroxy-benzoic-acid | Leaf | *Cocos nucifera (Coconut Leaf Extract)* |  | [135](https://pubchem.ncbi.nlm.nih.gov/compound/135) |
|  | phyllanthin | Leaf | *phyllanthus niruri ( Keelanelli)* |  | *358901* |
|  | Phyllemblin | Fruit | *Terminalia bellirica (Thanthrikai)* |  | 13250 |
|  | phyltetralin | Leaf | *phyllanthus niruri ( Keelanelli)* |  | *11223782* |
|  | phytosterol | Leaf | *Ocimum basilicum (Thiruneetrupachai)* |  | 222284 |
|  | phytosterols | Rhizome | *Zingiber officinale (Chukku)* |  | 12303662 |
|  |  | Root | *Curcuma longa (Turmeric)* |  | 12303662 |
|  | pipecolic-acid | Rhizome | *Zingiber officinale (Chukku)* |  | 849 |
|  | Piperanine | Flower, Root | *Piper longum (Thippili)* |  | 5320618 |
|  | Pipercallosine | Flower, Root | *Piper longum (Thippili)* |  | 5372201 |
|  | Piperchabamide B | Flower, Root | *Piper longum (Thippili)* |  | 44453655 |
|  | Piperchabamide C | Flower, Root | *Piper longum (Thippili)* |  | 44454018 |
|  | Piperchabamide D | Flower, Root | *Piper longum (Thippili)* |  | 16041827 |
|  | Piperdardine | Flower, Root | *Piper longum (Thippili)* |  | 10086948 |
|  | piperidine | seed | *Pepper nigrum (Pepper)* |  | *8082* |
|  | Piperine | Flower, Root | *Piper longum (Thippili)* |  | 638024 |
|  |  | seed | *Pepper nigrum (Pepper)* |  | 638024 |
|  | Piperitenone | Flower, Root | *Piper longum (Thippili)* |  | 381152 |
|  | Piperlongumine | Flower, Root | *Piper longum (Thippili)* |  | 637858 |
|  | Piperlonguminine | Flower, Root | *Piper longum (Thippili)* |  | 5320621 |
|  | Piperolactam A | Flower, Root | *Piper longum (Thippili)* |  | 3081016 |
|  | Piperonal | Flower, Root | *Piper longum (Thippili)* |  | 8438 |
|  | Piperonylic acid | Flower, Root | *Piper longum (Thippili)* |  | 7196 |
|  | Piperundecalidine | Flower, Root | *Piper longum (Thippili)* |  | 44453654 |
|  | Pisatin | Flower, Root | *Piper longum (Thippili)* |  | 101689 |
|  | Pisol | Flower, Root | *Piper longum (Thippili)* |  | 8193 |
|  | p-mentha-1,5-dien-7-ol | Rhizome | *Zingiber officinale (Chukku)* |  | 519721 |
|  | p-mentha-1-8-dien-7-ol | Rhizome | *Zingiber officinale (Chukku)* |  | 11788398 |
|  | p-mentha-2,8-dien-1-ol | Rhizome | *Zingiber officinale (Chukku)* |  | 155626 |
|  | p-Ocimene | Flower, Root | *Piper longum (Thippili)* |  | 5281553 |
|  | Polyprenol | leaves | *Azadirachta indica (Neem)* |  | 13453453 |
|  | preisocalamendiol | Rhizome | *Acorus calamus ( Vasambu)* |  | [12305705](https://pubchem.ncbi.nlm.nih.gov/compound/12305705) |
|  | proline | Fruit and pericarp | *Terminalia chebula (Kadukai)* |  | 614 |
|  |  | Leaf | *Cocos nucifera (Coconut Leaf Extract)* |  | 614 |
|  | propionaldehyde | Rhizome | *Zingiber officinale (Chukku)* |  | 527 |
|  | Protocatechuic acid | Leaves | *Pedalium murex ( Peru nerinji)* |  | 72 |
|  | pyran-4-carboxylic acid | Leaves | *Vitex negundo (Nochi)* |  | 20039212 |
|  | pyrethrine | Root | *Anacyclus pyrethrum (Akara)* |  | *5281045* |
|  | pyrocatechin | Leaf | *Piper betle ( Vetrilai)* |  | *289* |
|  | pyrrolidine | seed | *Pepper nigrum (Pepper)* |  | *31268* |
|  | pyrroperine | seed | *Pepper nigrum (Pepper)* |  | *636537* |
|  | quercetin | Fruit and pericarp | *Terminalia chebula (Kadukai)* |  | 5280343 |
|  |  | Leaf | *phyllanthus niruri ( Keelanelli)* |  | 5280343 |
|  |  | Leaf | *Azadirachta indica (Neem)* |  | [5280343](https://pubchem.ncbi.nlm.nih.gov/compound/5280343) |
|  | Quercetin 3-gentiobioside | Plant | *Solanum nigrum ( Manathakkali)* |  | *5320834* |
|  | Quercitrin | Leaf | *Azadirachta indica (Neem)* |  | [5280459](https://pubchem.ncbi.nlm.nih.gov/compound/5280459) |
|  | quinic acid | Fruit and pericarp | *Terminalia chebula (Kadukai)* |  | 6508 |
|  | raa | plant | *Justicia adhatoda (Aada thoda)* |  | 439710 |
|  | Resin | Root | *Abutilon indicum (Thuthi)* |  | 11290200 |
|  | Retrofractamide A | Flower, Root | *Piper longum (Thippili)* |  | 11012859 |
|  | Retrofractamide B | Flower, Root | *Piper longum (Thippili)* |  | 5372162 |
|  | riboflavin | Leaf | *Ocimum basilicum (Thiruneetrupachai)* |  | 493570 |
|  |  | Leaf | *Plectranthus amboinicus (Karppuravalli)* |  | 493570 |
|  |  | Fruit | *Solanum torvum (sundakai)* |  | 493570 |
|  | rosefuran | Rhizome | *Zingiber officinale (Chukku)* |  | 84825 |
|  | rosmarinic-acid | Leaf | *Ocimum tenuiflorum (Tulsi)* |  | *5281792* |
|  | rotundene | Root | *Cyprus rotundus (Korai kilangu)* |  | 25203405 |
|  | rotundone | Root | *Cyprus rotundus (Korai kilangu)* |  | *5321003* |
|  | Rubusic acid | Fruits | *Pedalium murex ( Peru nerinji)* |  | 101297651 |
|  | Rutin | Leaf | *Azadirachta indica (Neem)* |  | [5280805](https://pubchem.ncbi.nlm.nih.gov/compound/5280805) |
|  |  | Leaf | *Ocimum basilicum (Thiruneetrupachai)* |  | 5280805 |
|  |  | Fruit and pericarp | *Terminalia chebula (Kadukai)* |  | 5280805 |
|  | sabinene | Rhizome | *Acorus calamus ( Vasambu)* |  | [18818](https://pubchem.ncbi.nlm.nih.gov/compound/18818) |
|  | Safficinolide | leaf | *Salvia officinalis ( Sage)* |  | 85152699 |
|  | Sageone | leaf | *Salvia officinalis( Sage)* |  | 6481824 |
|  | salicylic-acid-2-beta-d-glucoside | Leaf | *Ocimum basilicum (Thiruneetrupachai)* |  | 7099939 |
|  | scopoletin | Plant | *Solanum nigrum ( Manathakkali)* |  | *5280460* |
|  | scutellarein | plant | *Clerodendrum serratum (Kanduparangi)* |  | 5281697 |
|  | Scyllitol | Leaf | *Cocos nucifera (Coconut Leaf Extract)* |  | 892 |
|  | sekishone | Rhizome | *Acorus calamus ( Vasambu)* |  | [636750](https://pubchem.ncbi.nlm.nih.gov/compound/636750) |
|  | selina-3,7(11)-diene | Rhizome | *Zingiber officinale (Chukku)* |  | 522296 |
|  | sennoside a | Fruit and pericarp | *Terminalia chebula (Kadukai)* |  | 73111 |
|  | Sesamin | Flower, Root | *Piper longum (Thippili)* |  | 72307 |
|  | Sesamol | Flower, Root | *Piper longum (Thippili)* |  | 68289 |
|  | sesquithujene | Leaf | *Ocimum basilicum (Thiruneetrupachai)* |  | 53359349 |
|  | [shikimic-acid](https://phytochem.nal.usda.gov/phytochem/chemicals/show/16263) | Fruit and pericarp | *Terminalia chebula (Kadukai)* |  | 8742 |
|  | shyobunone | Rhizome | *Acorus calamus ( Vasambu)* |  | [5321293](https://pubchem.ncbi.nlm.nih.gov/compound/5321293) |
|  | sinapic-acid | Leaf | *Ocimum tenuiflorum (Tulsi)* |  | *637775* |
|  | sitosterol | Rhizome | *Acorus calamus ( Vasambu)* |  | [222284](https://pubchem.ncbi.nlm.nih.gov/compound/222284) |
|  |  | Root | *Abutilon indicum (Thuthi)* |  | 222284 |
|  |  | Leaf | *Solanum trilobatum (Thoothuvalai)* |  | 222284 |
|  |  | Fruit | *Terminalia bellirica (Thanthrikai)* |  | 222284 |
|  |  | Root | *Cyprus rotundus (Korai kilangu)* |  | 222284 |
|  | sitosterol-d-glucoside | Fruit | *Solanum torvum (sundakai)* |  | *5742590* |
|  | skimmianine | Leaf | *Aegle marmelos (Vilvam)* |  | 6760 |
|  | Solanocapsine | Plant | *Solanum nigrum ( Manathakkali)* |  | *73419* |
|  | solasodine | Fruit | *Solanum torvum (sundakai)* |  | *442985* |
|  |  | Fruit | *Solanum nigrum ( Manathakkali)* |  | *442985* |
|  |  | Leaf | *Solanum nigrum ( Manathakkali)* |  | *442985* |
|  | sorbitol | Fruit and pericarp | *Terminalia chebula (Kadukai)* |  | 5780 |
|  | Spirostan-3-ol | Plant | *Solanum nigrum ( Manathakkali)* |  | *3035446* |
|  | squalene | Leaves | *Vitex negundo (Nochi)* |  | 638072 |
|  |  | leaves | *Azadirachta indica (Neem)* |  | 638072 |
|  | stigmasterol | Plant extract | *Ipomea carnea (Neiveli Kaatamanakku)* |  | 5280794 |
|  |  | Leaf | *Ocimum basilicum (Thiruneetrupachai)* |  | 5280794 |
|  |  | Leaf | *Solanum trilobatum (Thoothuvalai)* |  | 5280794 |
|  |  | Root | *Curcuma longa (Turmeric)* |  | 5280794 |
|  |  | leaves | *Azadirachta indica (Neem)* |  | 5280794 |
|  | syringaldehyde | Leaf | *Cocos nucifera (Coconut Leaf Extract)* |  | [8655](https://pubchem.ncbi.nlm.nih.gov/compound/8655) |
|  | Syringaresinol | Plant | *Solanum nigrum ( Manathakkali)* |  | *100067* |
|  | syringic-acid | Leaf | *Cocos nucifera (Coconut Leaf Extract)* |  | [10742](https://pubchem.ncbi.nlm.nih.gov/compound/10742) |
|  | Tannin | Root, Leaves, stem | *Abutilon indicum (Thuthi)* |  | 7115 |
|  | taraxerol | Stem | *Cissus_quadrangularis_L (Perandai)* |  | *92097* |
|  | taraxeryl acetate | Stem | *Cissus_quadrangularis_L (Perandai)* |  | *94225* |
|  | [termilignan](https://phytochem.nal.usda.gov/phytochem/chemicals/show/27029) | Pericarp | *Terminalia bellirica (Thanthrikai)* |  | 466076 |
|  | terpinen-4-ol | Rhizome | *Acorus calamus ( Vasambu)* |  | [11230](https://pubchem.ncbi.nlm.nih.gov/compound/11230) |
|  | terpinene | Leaf | *Ocimum basilicum (Thiruneetrupachai)* |  | 7461 |
|  | terpinolene | Rhizome | *Acorus calamus ( Vasambu)* |  | 11463 |
|  |  | Leaf | *Ocimum tenuiflorum (Tulsi)* |  | *11463* |
|  | terpinyl acetate | Leaves | *Vitex negundo (Nochi)* |  | 111037 |
|  | tetradecadiene-1,13 | Flower, Root | *Piper longum (Thippili)* |  | 30875 |
|  | Tetradecahydro-1-methylphenanthrene | Flower, Root | *Piper longum (Thippili)* |  | 609802 |
|  | thannilignan | Pericarp | *Terminalia bellirica (Thanthrikai)* |  | 466077 |
|  | thiamin | Leaf | *Ocimum basilicum (Thiruneetrupachai)* |  | 1130 |
|  |  | Leaf | *Plectranthus amboinicus (Karppuravalli)* |  | 1130 |
|  | thymol | Leaf | *Ocimum basilicum (Thiruneetrupachai)* |  | 6989 |
|  | trans-anethole | Flower | *Syzygium aromaticum (Lavangam)* |  | 637563 |
|  | trans-isoelemicine | Rhizome | *Acorus calamus ( Vasambu)* |  | 5318557 |
|  | trans-geranic-acid | Rhizome | *Zingiber officinale (Chukku)* |  | 5275520 |
|  | trans-linalol-oxide | Rhizome | *Zingiber officinale (Chukku)* |  | 6427788 |
|  | trans-octen-2-al | Rhizome | *Zingiber officinale (Chukku)* |  | 16900 |
|  | trans-rose-oxide | Rhizome | *Zingiber officinale (Chukku)* |  | 1712086 |
|  | tricyclene | Rhizome | *Zingiber officinale (Chukku)* |  | 79035 |
|  |  | Flower, Root | *Piper longum (Thippili)* |  | 45934475 |
|  | Tridecane | Flower, Root | *Piper longum (Thippili)* |  | 12388 |
|  | Tridecylene | Flower, Root | *Piper longum (Thippili)* |  | 17095 |
|  | Triterpenoid | Leaves | *Abutilon indicum (Thuthi)* |  | 451674 |
|  | turmerone | Flower, Root | *Piper longum (Thippili)* |  | 558221 |
|  | tyrosine | Leaf | *Cocos nucifera (Coconut Leaf Extract)* |  | [6057](https://pubchem.ncbi.nlm.nih.gov/compound/6057) |
|  | undecan-2-ol | Rhizome | *Zingiber officinale (Chukku)* |  | 15448 |
|  | Undulatone | Flower, Root | *Piper longum (Thippili)* |  | 5281311 |
|  | uridine | Rhizome | *Zingiber officinale (Chukku)* |  | 6029 |
|  | Ursolic acid | Fruits | *Pedalium murex ( Peru nerinji)* |  | 64945 |
|  |  | Fruit | *Malus domestica (Apple)* |  | 64945 |
|  |  | Leaf | *Ocimum basilicum (Thiruneetrupachai)* |  | 64945 |
|  |  | Leaf | *Ocimum tenuiflorum (Tulsi)* |  | 64945 |
|  | Valencene | Flower, Root | *Piper longum (Thippili)* |  | 9855795 |
|  | valine | Leaf | *Cocos nucifera (Coconut Leaf Extract)* |  | [6287](https://pubchem.ncbi.nlm.nih.gov/compound/6287) |
|  | vanillic acid | Leaves | *Pedalium murex ( Peru nerinji)* |  | 8468 |
|  |  | Leaf | *Cocos nucifera (Coconut Leaf Extract)* |  | [8468](https://pubchem.ncbi.nlm.nih.gov/compound/8468) |
|  | vanillic-acid-4-beta-d-glucoside | Leaf | *Ocimum basilicum (Thiruneetrupachai)* |  | 14132337 |
|  | vanillin | Leaf | *Cocos nucifera (Coconut Leaf Extract)* |  | [1183](https://pubchem.ncbi.nlm.nih.gov/compound/1183) |
|  |  | Fruits | *Pedalium murex ( Peru nerinji)* |  | 1183 |
|  | vasicine | Leaf | *Justicia adhatoda (Aada thoda)* |  | 667496 |
|  |  | plant | *Justicia adhatoda (Aada thoda)* |  | [667496](https://pubchem.ncbi.nlm.nih.gov/compound/667496) |
|  | vasicinol | plant | *Justicia adhatoda (Aada thoda)* |  | 442934 |
|  | vasicinone | plant | *Justicia adhatoda (Aada thoda)* |  | 442935 |
|  | vasicol | plant | *Justicia adhatoda (Aada thoda)* |  | 92470596 |
|  | vasicoline | plant | *Justicia adhatoda (Aada thoda)* |  | 626005 |
|  | vasicolinone | plant | *Justicia adhatoda (Aada thoda)* |  | 627712 |
|  | vicenin-2 | Leaf | *Ocimum basilicum (Thiruneetrupachai)* |  | 442664 |
|  | viridiflorol | Leaves | *Vitex negundo (Nochi)* |  | 11996452 |
|  | Xanthoangelol | leaves, fruit | *carica papaya (Papaya)* |  | 11302670 |
|  | xanthomicrol | Leaf | *Ocimum basilicum (Thiruneetrupachai)* |  | 73207 |
|  | xanthorrhizol | Rhizome | *Zingiber officinale (Chukku)* |  | 93135 |
|  | zerumbodienone | Rhizome | *Zingiber officinale (Chukku)* |  | 5463722 |
|  | zingiberene | Rhizome | *Zingiber officinale (Chukku)* |  | 92776 |
|  |  | Root | *Curcuma longa (Turmeric)* |  | 92776 |
|  | zingiberenol | Rhizome | *Zingiber officinale (Chukku)* |  | 13213649 |
|  | zingiberol | Rhizome | *Zingiber officinale (Chukku)* |  | 5317270 |
|  | zonarene | Flower | *Syzygium aromaticum (Lavangam)* |  | 6428488 |
|  | α-copaene | Leaves | *Vitex negundo (Nochi)* |  | 19725 |
|  | α-thujene | Leaves | *Vitex negundo (Nochi)* |  | 17868 |
|  | β- farnesene | Leaves | *Vitex negundo (Nochi)* |  | 5281517 |
|  | β‑amyrin | Stem | *Cissus_quadrangularis_L (Perandai)* |  | *92156* |
|  | β-bisabolol | Leaves | *Vitex negundo (Nochi)* |  | 1549992 |
|  | β-caryophyllene | Leaves | *Vitex negundo (Nochi)* |  | 5281515 |
|  | β-eudesmol | Leaves | *Vitex negundo (Nochi)* |  | 91457 |
|  | β-sitosterol | plant | *Clerodendrum serratum (Kanduparangi)* |  | 222284 |
|  |  | Leaf | *Azadirachta indica (Neem)* |  | [222284](https://pubchem.ncbi.nlm.nih.gov/compound/222284) |
|  |  | Stem | *Cissus_quadrangularis_L (Perandai)* |  | [222284](https://pubchem.ncbi.nlm.nih.gov/compound/222284) |
|  |  | Root | *Curcuma longa (Turmeric)* |  | [222284](https://pubchem.ncbi.nlm.nih.gov/compound/222284) |
|  |  | Plant extract | *Ipomea carnea (Neiveli Kaatamanakku)* |  | 222284 |
|  |  | Fruits | *Pedalium murex ( Peru nerinji)* |  | 222284 |
|  |  | Fruit and pericarp | *Terminalia chebula (Kadukai)* |  | 222284 |

| **Table S1: b) LIST OF COMPOUNDS RETRIEVED FOR COVID 19 FROM DRUG BANK** | | |
| --- | --- | --- |
| **S.NO** | **PUCHEM ID** | **COMPOUND NAME** |
|  | 145998218 | 1,2-Ethanediol |
|  | 124547413 | Acetate |
|  | 94171767 | Acetic acid |
|  | 83683266 | Benzyl alcohol |
|  | 81371985 | Formic acid |
|  | 79838750 | Citric acid |
|  | 72107716 | Chloride ion |
|  | 62755740 | Dimethyl sulfoxide |
|  | 60655566 | Glycine |
|  | 60645778 | Glycerol |
|  | 60635968 | Sulfite |
|  | 60634762 | Sulfate |
|  | 52361231 | Ambroxol |
|  | 52113406 | Chloroquine |
|  | 40505715 | Hydroxychloroquine |
|  | 40476772 | Sulfapyridine |
|  | 40004840 | Thalidomide |
|  | 25126798 | Leucine |
|  | 24701445 | DI(Hydroxyethyl)ether |
|  | 23559815 | Tetraethylene glycol |
|  | 20786326 | 2-Hydroxypyridine |
|  | 20754800 | N-Acetyl-beta-D-glucosamine |
|  | 19325375 | beta-D-Fructopyranose |
|  | 19325187 | Ribavirin |
|  | 17754054 | 2-[N-Cyclohexylamino]ethane sulfonic acid |
|  | 9234227 | 4-Bromobenzenesulfonamide |
|  | 8405578 | N-((4-(Aminosulphonyl)phenyl)methyl)acetamide |
|  | 7510236 | 5-Methylisoxazole-3-carboxylic acid |
|  | 7063878 | 2-(N-Morpholino)-ethanesulfonic acid |
|  | 6952762 | Methyl 4-sulfamoylbenzoate |
|  | 6346752 | Piperazine, 1,4-diacetyl |
|  | 4379552 | Methanesulfonamide |
|  | 4338791 | 3-Hydroxythietane 1,1-dioxide |
|  | 3803220 | N-(1,3-Benzodioxol-5-ylmethyl)acetamide |
|  | 3784209 | Pyrimidin-5-amine |
|  | 3759658 | beta-D-Mannose |
|  | 3695997 | Azepan-1-yl(1,3-benzodioxol-5-yl)methanone |
|  | 3511405 | 1-Phenyl-3-pyridin-3-ylurea |
|  | 3282410 | N-[2-(5-Fluoro-1H-indol-3-yl)ethyl]acetamide |
|  | 2815672 | 1-Cyclohexyl-3-(2-pyridin-4-ylethyl)urea |
|  | 2806372 | 4-[2-(Phenylsulfanyl)ethyl]morpholine |
|  | 2566008 | 1-[4-[(5-Bromothiophen-2-yl)methyl]piperazin-1-yl]ethanone |
|  | 2140084 | N~1~-Phenyl-1,4-piperidinedicarboxamide |
|  | 1487531 | (3-Fluoro-benzyl)-furan-2-ylmethyl-amine |
|  | 1353764 | 1-[4-(Naphthalen-1-ylmethyl)piperazin-1-yl]ethanone |
|  | 1224835 | 4-(3-Chlorobenzyl)morpholinef |
|  | 1086839 | 2-[(1H-Benzimidazol-2-ylamino)methyl]phenol |
|  | 1072430 | N-[3-(2-Oxopyrrolidin-1-yl)phenyl]acetamide |
|  | 852723 | Methyl 2-methyl-4-phenyl-1,3-thiazol-5-ylcarbamate |
|  | 831828 | 1-[4-(4-Methoxyphenyl)piperazin-1-yl]ethan-1-one |
|  | 811874 | Acetamide, N-[(1R)-1-(1-naphthalenyl)ethyl]- |
|  | 790557 | 2-Cyclohexyl-N-(3-pyridyl)acetamide |
|  | 787400 | (2S)-N-(4-Carbamoylphenyl)oxolane-2-carboxamide |
|  | 782662 | 1-Methyl-N-[[(2S)-oxolan-2-yl]methyl]pyrazole-3-carboxamide |
|  | 782539 | N-[(3S)-1,1-Dioxo-2,3-dihydrothiophen-3-yl]-N-phenylacetamide |
|  | 777700 | N-[4-(Pyrimidin-2-yloxy)phenyl]acetamide |
|  | 769265 | N-[(3R)-1,1-Dioxo-2,3-dihydrothiophen-3-yl]-N-(4-methylphenyl)acetamide |
|  | 743462 | 1-[4-(5-Chlorothiophen-2-yl)sulfonylpiperazin-1-yl]ethanone |
|  | 739875 | Methyl 3-(Methylsulfonylamino)benzoate |
|  | 712045 | N-[(4-Cyanophenyl)methyl]morpholine-4-carboxamide |
|  | 699127 | 1-[4-(2-Fluorophenyl)sulfonylpiperazin-1-yl]ethanone |
|  | 674807 | N-(4-Methoxy-1,3-benzothiazol-2-yl)acetamide |
|  | 673374 | 1-[4-(3-Fluorobenzenesulfonyl)piperazin-1-yl]ethan-1-one |
|  | 439680 | 1-(4-Thiophen-2-ylsulfonylpiperazin-1-yl)ethanone |
|  | 344373 | 1-[4-(2,6-Difluorophenyl)sulfonylpiperazin-1-yl]ethanone |
|  | 290549 | 2-(4-Acetylpiperazin-1-yl)sulfonylbenzonitrile |
|  | 265635 | N-(2-Chloropyridin-3-yl)acetamide |
|  | 118569 | 1-(4-(2-Nitrophenyl)piperazin-1-yl)ethanone |
|  | 95851 | 1-Azanylpropylideneazanium |
|  | 6952762 | 2-(4-methylphenoxy)-1-(4-methylpiperazin-4-ium-1-yl)ethanone |
|  | 78165 | 2-[(Methylsulfonyl)methyl]-1H-benzimidazole |
|  | 76947 | 1-Acetyl-4-[(2,5-dimethylphenyl)sulfonyl]piperazine |
|  | 74833 | 1-Acetyl-4-[(4-chlorophenyl)sulfonyl]piperazine |
|  | 69696 | 1-[(3R)-3-(1,3-Benzothiazol-2-yl)piperidin-1-yl]ethanone |
|  | 66898 | Lysine Nz-Carboxylic Acid |
|  | 37542 | 1-[4-[(3-Chlorophenyl)methyl]piperazin-1-yl]ethanone |
|  | 24310 | 1-[4-[(3-Methylphenyl)methyl]piperazin-1-yl]ethanone |
|  | 24139 | N-(1-Acetyl-4-piperidinyl)benzamide |
|  | 8871 | N-(4-Methylpyridin-3-yl)acetamide |
|  | 8200 | (E,4S)-4-Azanyl-5-[(3S)-2-oxidanylidenepyrrolidin-3-yl]pent-2-enoic acid |
|  | 8117 | 6-(Ethylamino)nicotinonitrile |
|  | 6106 | Ruxolitinib |
|  | 5426 | 1-[4-(Thiophen-3-ylmethyl)piperazin-1-yl]ethanone |
|  | 5336 | 1-(Thiophen-3-ylmethyl)piperidin-4-ol |
|  | 3652 | N-[(3R)-1,1-Dioxo-2,3-dihydrothiophen-3-yl]-N-(4-fluorophenyl)acetamide |
|  | 2719 | 1-[(2R)-2-(4-Fluorophenyl)morpholin-4-yl]ethanone |
|  | 2132 | 1-[4-[(2-Methylphenyl)methyl]-1,4-diazepan-1-yl]ethanone |
|  | 1117 | 1-[4-(Piperidine-1-carbonyl)piperidin-1-yl]ethanone |
|  | 1099 | 1-[4-(Thiophene-2-carbonyl)piperazin-1-yl]ethan-1-0ne |
|  | 753 | 1-{4-[(5-Chlorothiophen-2-yl)methyl]piperazin-1-yl}ethan-1-one |
|  | 750 | 1-[4-(Thiophen-2-ylmethyl)piperazin-1-yl]ethanone |
|  | 679 | Ethyl[(1-methyl-1H-1,2,3-triazol-4-yl)methyl]amine |
|  | 312 | 1-Methyl-1,2,3,4-tetrahydroquinoline-7-sulfonamide |
|  | 311 | 5-(1,4-Oxazepan-4-YL)pyridine-2-carbonitrile |
|  | [284](https://pubchem.ncbi.nlm.nih.gov/compound/81371985) | N-[(1S)-1-(3-Chlorophenyl)ethyl]acetamide |
|  | 244 | (1-Pyridin-2-yl-cyclopentyl)-methanol |
|  | 94171767 | 1-[(2S)-2-methylmorpholin-4-yl]-2-pyrazol-1-ylethanone |
|  | 175 | (2R,3R)-1-Benzyl-2-methylpiperidin-3-ol |
|  | 145998218 | 1-(4-methylpiperazin-1-yl)-2-(1H-pyrrolo[2,3-b]pyridin-3-yl)ethanone |

| **Table S1: c) ANTIVIRAL COMPOUNDS RETRIEVED FROM PUBCHEM** | | |
| --- | --- | --- |
| **S.NO** | **PUCHEM ID** | **COMPOUND NAME** |
|  | [72276](https://pubchem.ncbi.nlm.nih.gov/compound/72276) | (-)-epicatechin |
|  | 107905 | (-)-epicatechin-3-o-gallate |
|  | 471393 | (+)-catechin-7-o-gallate |
|  | 157057 | 2,7-dihydroxycadalene |
|  | 5316900 | 3,3'-dimethylquercetin |
|  | 581654 | 3-methylquercetin |
|  | 44584640 | 3-o-trans-caffeoyltormentic-acid |
|  | 676296 | 4 hydroxy 3' methoxyflavone |
|  | 5486942 | 4',7-dihydroxy-3-methoxy-5,6-dimethylflavone |
|  | 5281677 | 5,4'-dihydroxy-3,7,3'-trimethoxyflavone |
|  | 44559503 | 6-hydroxycrinamine |
|  | 6440422 | 8-methoxy-psoralen |
|  | 97283 | 10-methoxycamptothecin |
|  | 10414856 | 13',ii8-biapigenin |
|  | 5281599 | agathisflavone |
|  | 5386591 | ajoene |
|  | 6850754 | alginic-acid |
|  | 65036 | allicin |
|  | 7858 | allyl-alcohol |
|  | 1007 | aloe-emodin |
|  | 1305761 | aloin |
|  | 10340689 | alpha-apopicropodophyllotoxin |
|  | 9129 | alpha-peltatin |
|  | 6654 | alpha-pinene |
|  | 5281600 | amentoflavone |
|  | 44938 | anagyrine |
|  | 10658 | angelicin |
|  | 97722 | anhydropodophyllol |
|  | 145858 | anthocyanin |
|  | 5280443 | apigenin |
|  | 454878 | apogossypol |
|  | 5459045 | arbortristoside |
|  | 64981 | arctigenin |
|  | 92139 | ar-curcumene |
|  | 2236 | aristolochic-acid |
|  | 68827 | artemisinin |
|  | 54670067 | ascorbic-acid |
|  | 10219 | emetin |
|  | 3034034 | quinine |
|  | 445154 | Resveratrol |
