# Supplementary material for: Exploring Phytochemicals of Traditional Medicinal Plants Exhibiting Inhibitory Activity Against Main Protease, Spike Glycoprotein, RNA-dependent RNA Polymerase and Non-Structural Proteins of SARS-CoV-2 Through Virtual Screening
Source: Front Pharmacol. 2021 Jul 8;12:667704. doi: 10.3389/fphar.2021.667704 (PMC8295902; doi:10.3389/fphar.2021.667704)
Supplement: Supplementary file 3 [file Table3.docx]

**Table S3** 2D interaction of Agasthisflavone and Amentoflavone with SARS CoV M^Pro,^ MERS CoV2 M^Pro^ and seven proteins of SARS CoV2.

| Protein | Agathisflavone (2D protein-ligand interaction/Binding affinity) | Amentoflavone (2D protein-ligand interaction/Binding affinity) |  |
| --- | --- | --- | --- |
| SARS CoV2 M^Pro^ | 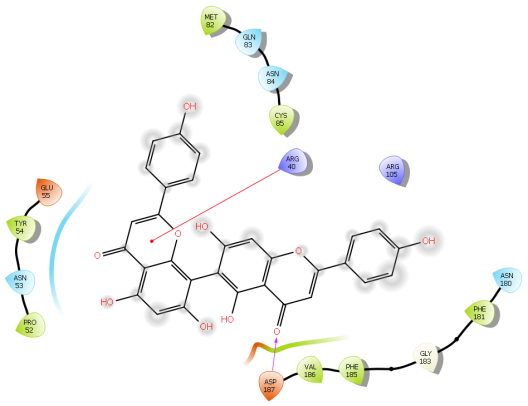  -8.2kcal/mol | 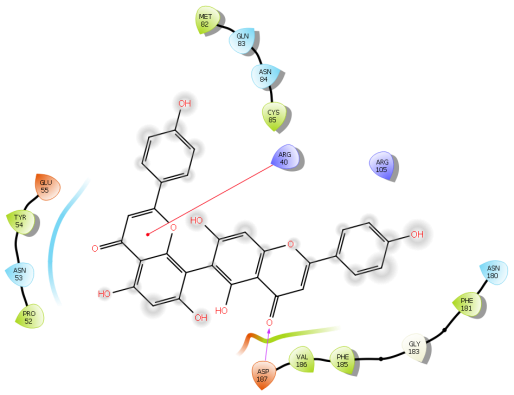-7.5kcal/mol |  |
| RdRp | 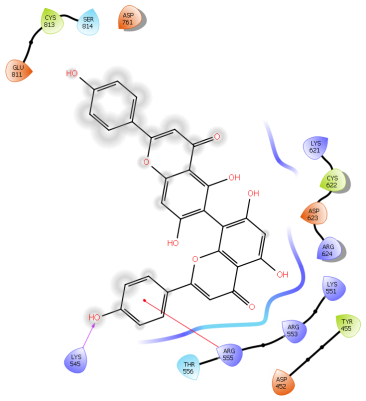  -8.9 kcal/mol | 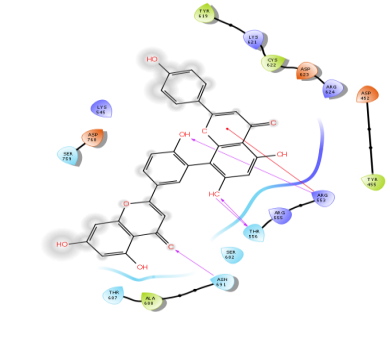  -9.3 kcal/mol |  |
| NSP3 | 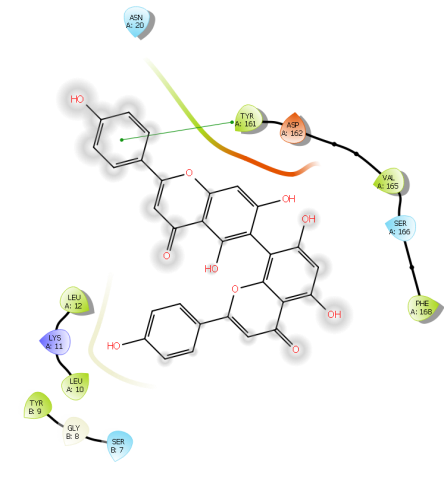  -6.6 kcal/mol | 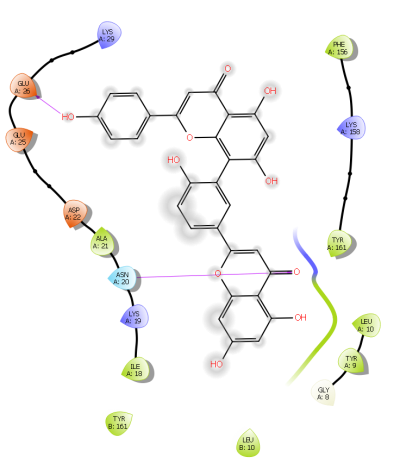  -7.4 kcal/mol |  |
| NSP9 | 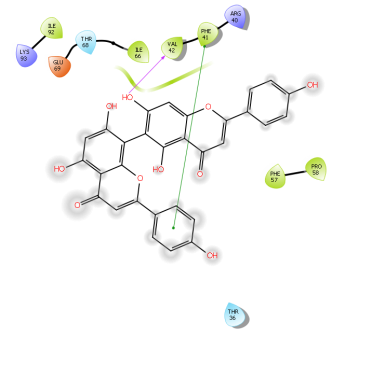  -8.1 kcal/mol | 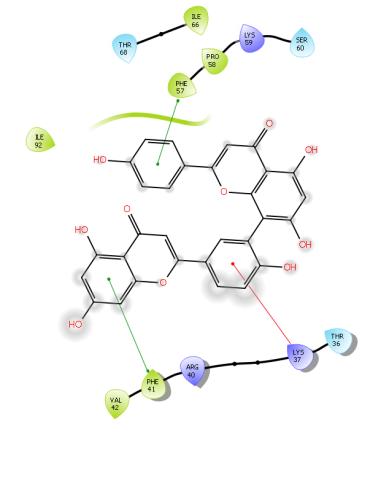  -8.3 kcal/mol |  |
| NSP16 - NSP10 | 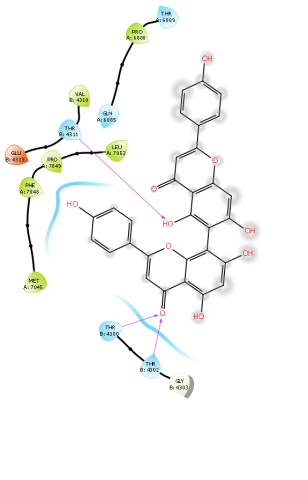  -7.3 kcal/mol | 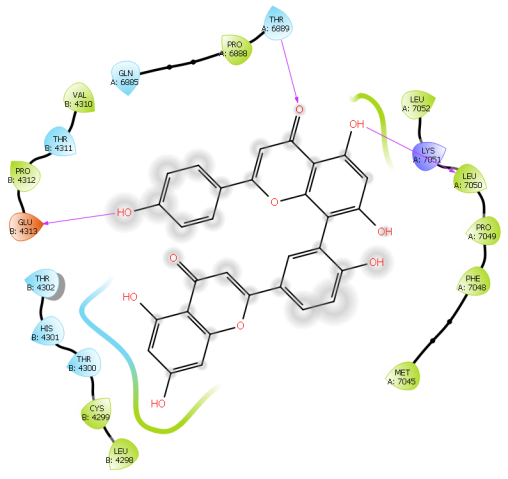  -8.5 kcal/mol |  |
| NSP15 | 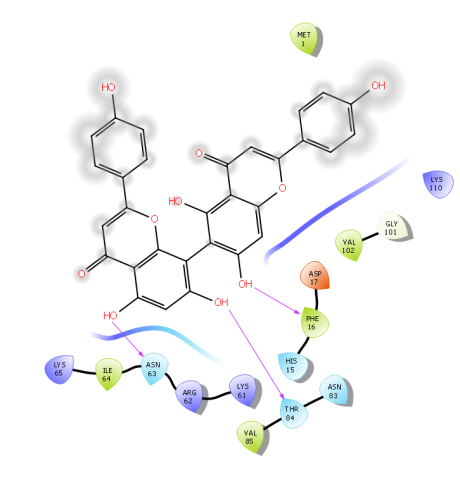  -8.2kcal/mol | 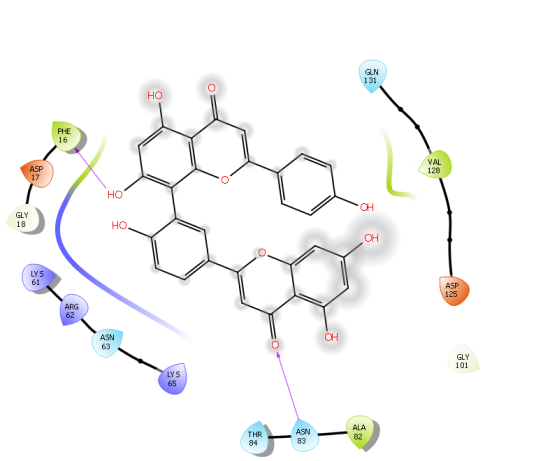  -8.4kcal/mol |  |
| Spike glycoprotein | 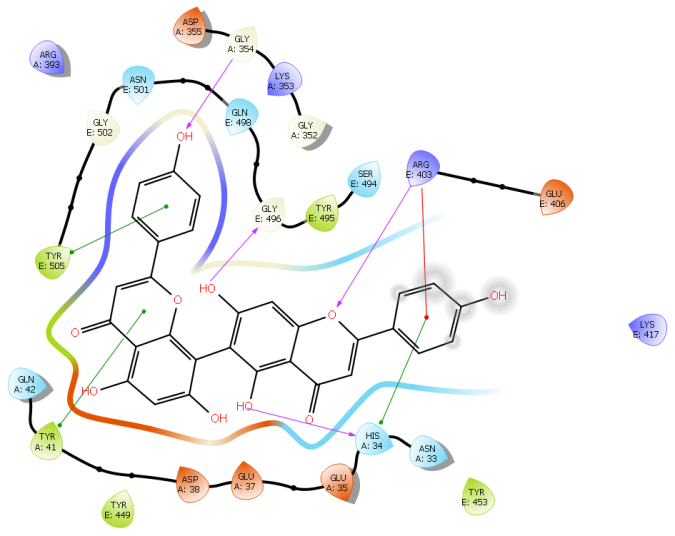  -8.2kcal/mol | 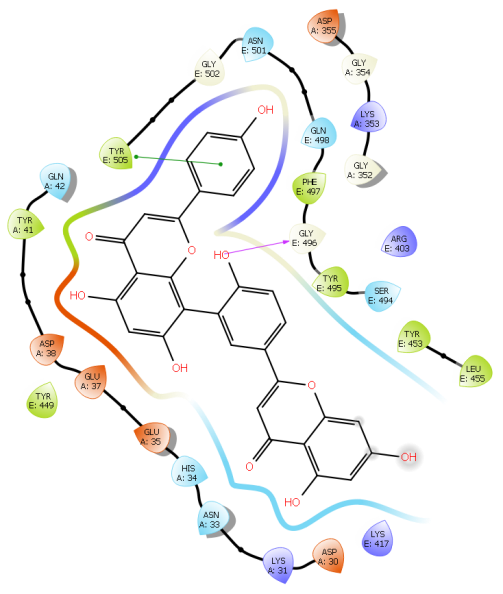  -8.2 kcal/mol |  |

**Table S4** 2D interaction of Hydroxychloroquine, Chloroquine and Ivermectin against seven target proteins of SARS CoV2

| Protein | **Hydroxycholoroquine (**(2D protein-ligand interaction/Binding affinity) | Chloroquine (2D protein-ligand interaction/Binding affinity) | Ivermectin (2D protein-ligand interaction/Binding affinity) |
| --- | --- | --- | --- |
| SARS CoV2 M^Pro^ | 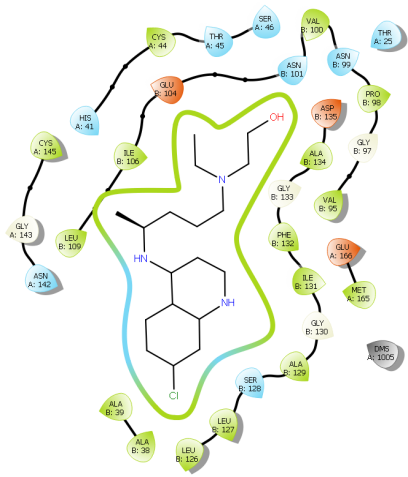 | 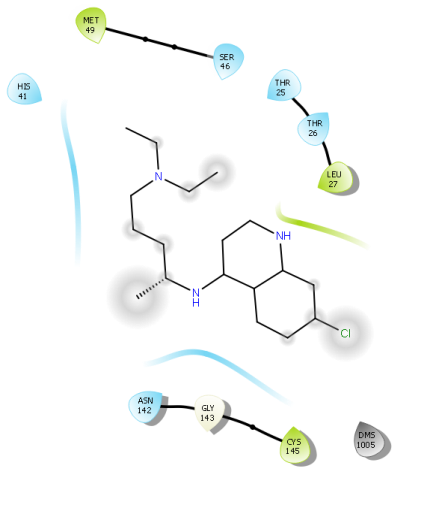 | 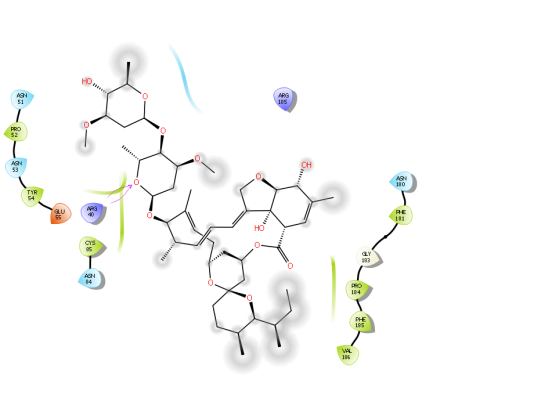 |
|  | -5.5kcal/mol | -4.9kcal/mol | -7.3kcal/mol |
| RdRp | 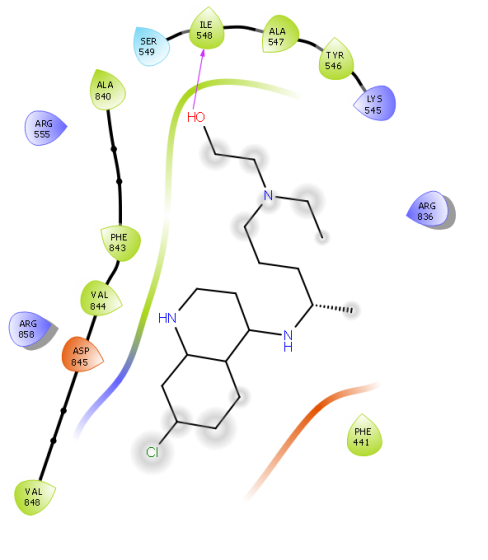 | 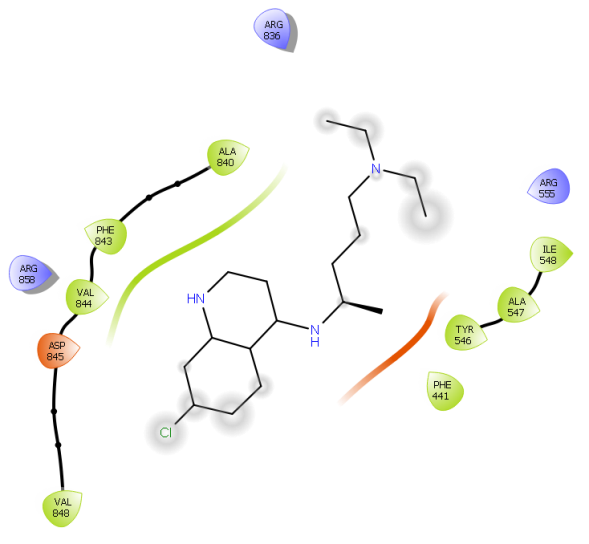 | 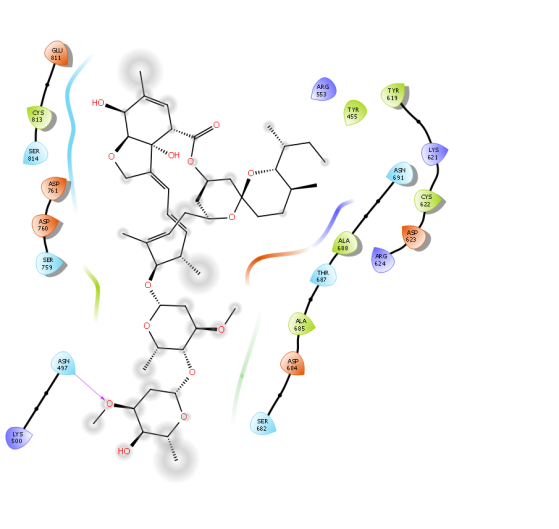 |
|  | -5.6kcal/mol | -5.4kcal/mol | -9.4kcal/mol |
| NSP3 | 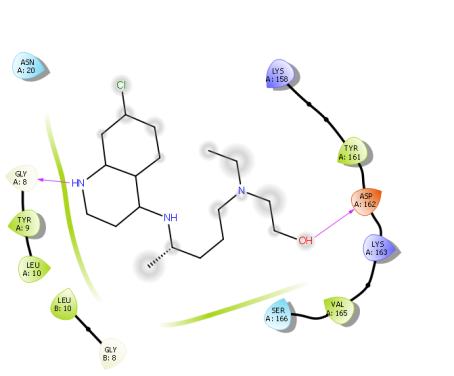 | 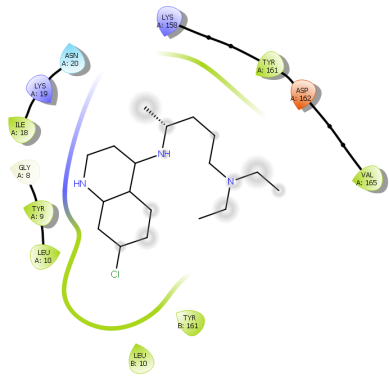 | 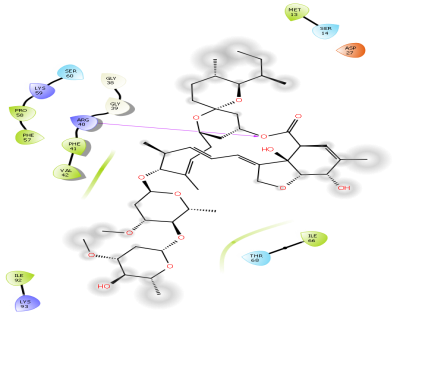 |
|  | -4.5kcal/mol | -4.2kcal/mol | -6.7kcal/mol |
| NSP9 | 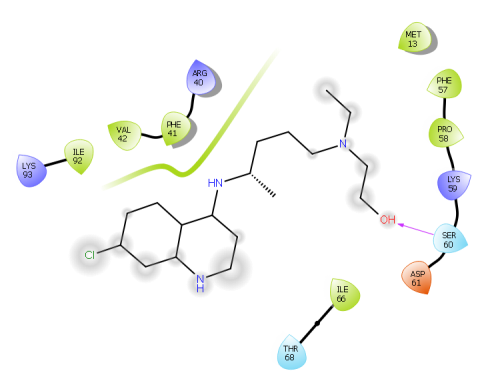 | **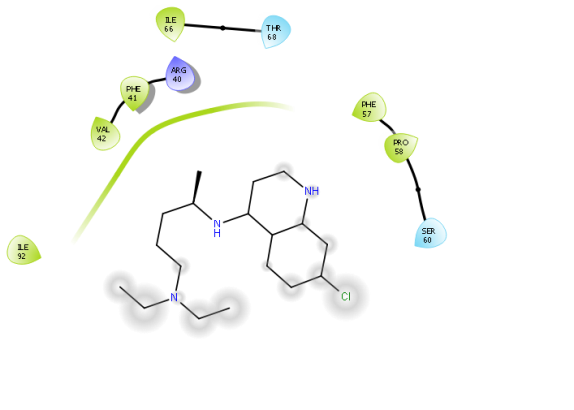** | **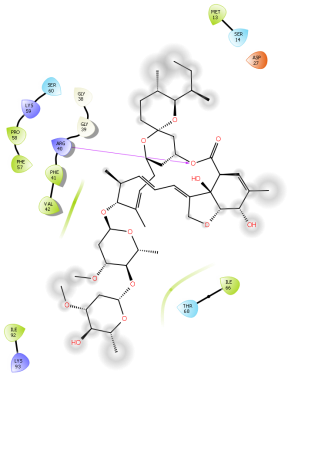** |
|  | -5.7kcal/mol | -5.2kcal/mol | -7.5 kcal/mol |
| NSP16-NSP10 | 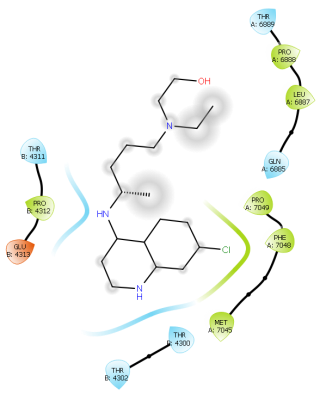 | 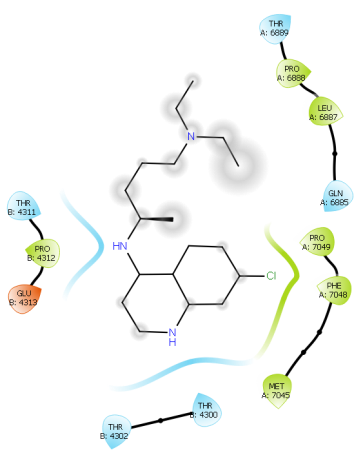 | 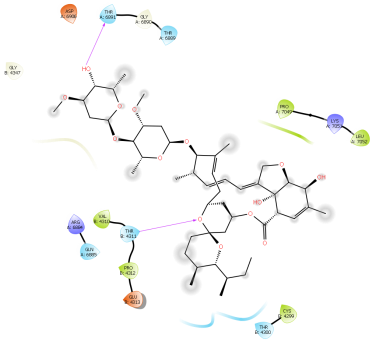 |
|  | -5.7kcal/mol | -5.3kcal/mol | -1.9kcal/mol |
| NSP15 | 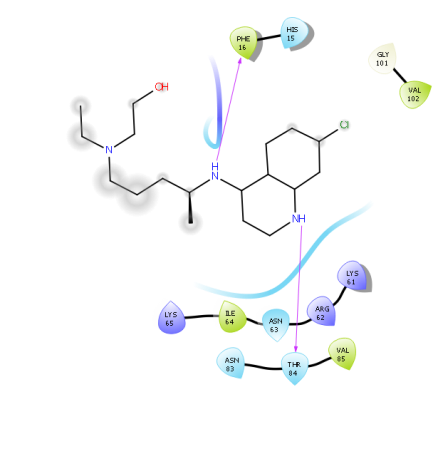 | 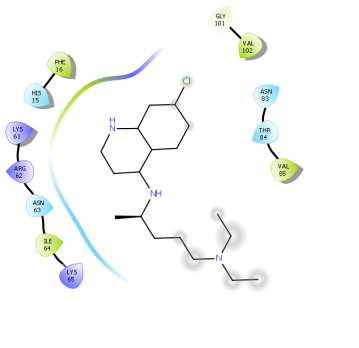 | 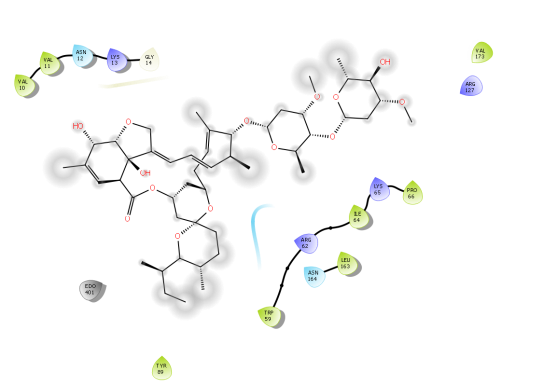 |
|  | -5.5 kcal/mol | -5.4kcal/mol | -6.4 kcal/mol |
| Spike glycoprotein | 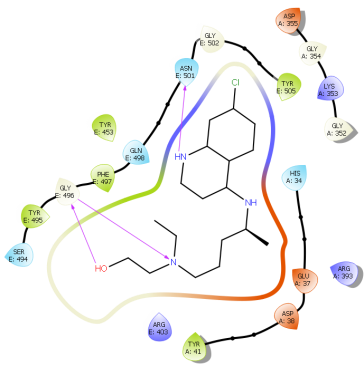 | 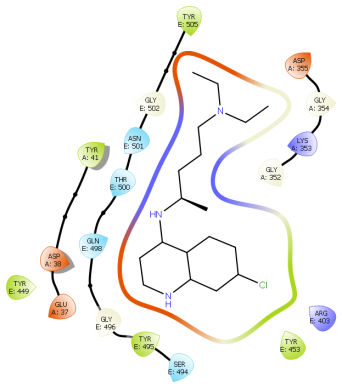 | 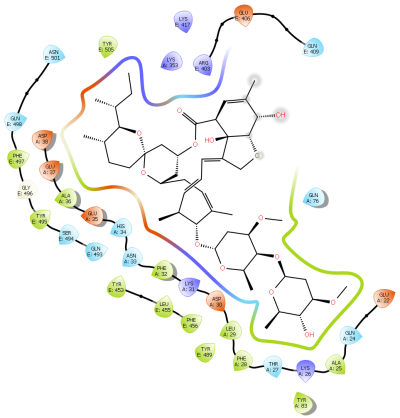 |
|  | -5.3kcal/mol | -5.2kcal/mol | -8.2kcal/mol |

**Table S5** 2D interaction of cyanin with SARS CoV M^Pro,^ MERS CoV2 M^Pro^ and seven proteins of SARS CoV2

| Protein | Protein-ligand interaction | Residues exhibiting H Bond interactions |
| --- | --- | --- |
| **SARS COV Main Protease** | 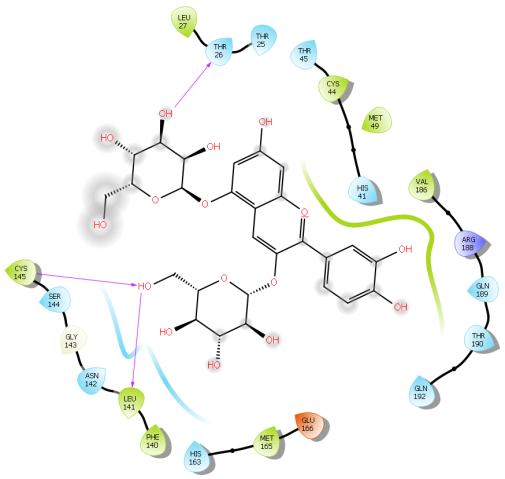  -8.2 kcal/mol | T26, L141,C145 |
| **SARS COV2 Main Protease** | 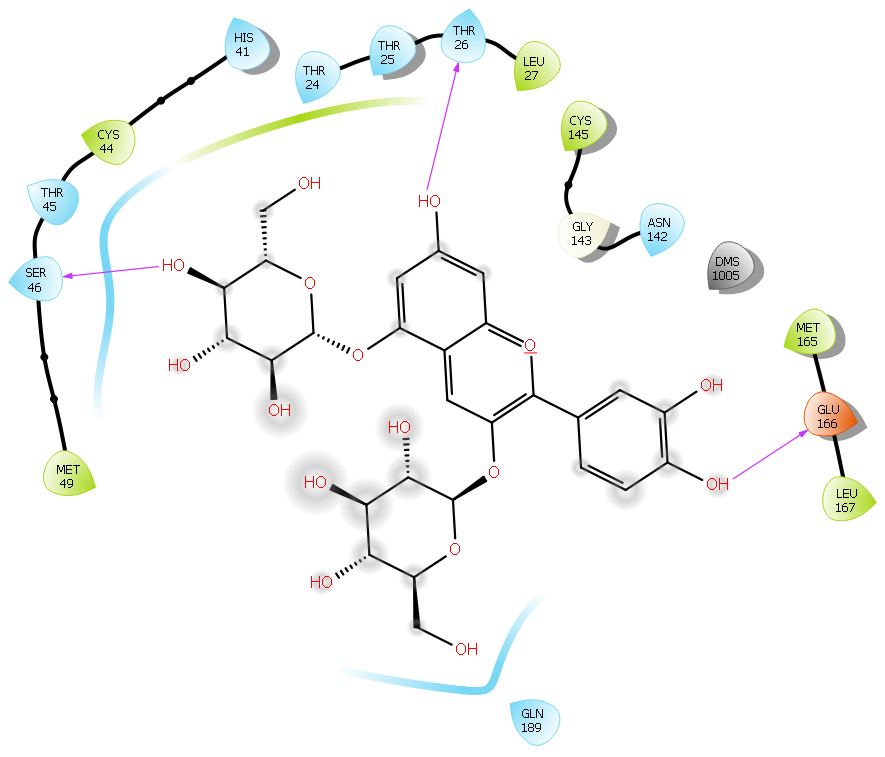-8.3 kcal/mol | T26, S45, D166 |
| **MERS COV Main Protease** | 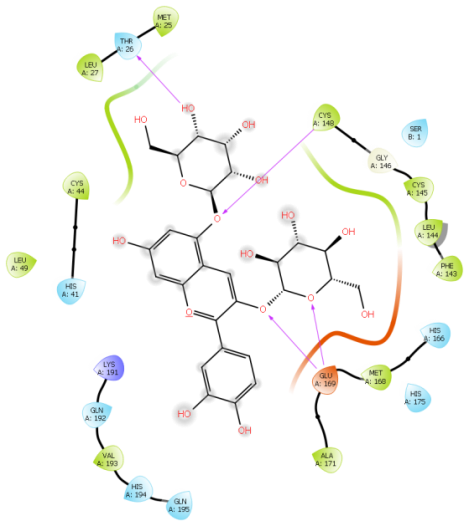  -7.7 kcal/mol | T26, C149, D169 |
